# Effects of Early Diet on the Prevalence of Allergic Disease in Children: A Systematic Review and Meta-Analysis

**DOI:** 10.1016/j.advnut.2023.10.001

**Published:** 2023-10-10

**Authors:** Shumin Wang, Pingping Yin, Leilei Yu, Fengwei Tian, Wei Chen, Qixiao Zhai

**Affiliations:** 1 State Key Laboratory of Food Science and Resources, Jiangnan University, Wuxi, Jiangsu, China; 2 School of Food Science and Technology, Jiangnan University, Wuxi, Jiangsu, China; 3 National Engineering Research Center for Functional Food, Jiangnan University, Wuxi, Jiangsu, China

**Keywords:** Early diet, complementary foods, infant, allergic disease, food allergies, asthma, atopic dermatitis, systematic review, meta-analysis

## Abstract

Recent evidence suggests that the timing of introduction, types, and amounts of complementary foods/allergenic foods may influence the risk of allergic disease. However, the evidence has not been updated and comprehensively synthesized. The Cochrane Library, EMBASE, Web of Science, and PubMed databases were searched from the inception of each database up to 31 May 2023 (articles prior to 2000 were excluded manually). Statistical analyses were performed using RevMan 5. The GRADE approach was followed to rate the certainty of evidence. Compared with >6 mo, early introduction of eggs (≤6 mo of age) might reduce the risk of food allergies in preschoolers aged <6 y (odds ratio [OR], 0.65; 95% confidence interval [CI], 0.53, 0.81), but had no effect on asthma or atopic dermatitis (AD). Consumption of fish at 6–12 mo might reduce the risk of asthma in children (aged 5–17 y) compared with late introduction after 12 mo (OR, 0.61; 95% CI: 0.52, 0.72). Introduction of allergenic foods for ≤6 mo of age, compared with >6 mos, was a protective factor for the future risk (children aged ≤10 y) of AD (OR, 0.93; 95% CI: 0.89, 0.97). Probiotic intervention for infants at high risk of allergic disease significantly reduced the risk of food allergy at ages 0–3 y (OR, 0.72; 95% CI: 0.56, 0.94), asthma at 6–12 y (OR, 0.61; 95% CI: 0.41, 0.90), and AD at aged <6 y (3–6 y: OR, 0.70; 95% CI: 0.52, 0.94; 0–3 y: OR, 0.73; 95% CI: 0.59, 0.91). Early introduction of complementary foods or the high-dose vitamin D supplementation in infancy was not associated with the risk of developing food allergies, asthma, or AD during childhood. Early introduction to potential allergen foods for normal infants or probiotics for infants at high risk of allergies may protect against development of allergic disease. This study was registered at PROSPERO as CRD42022379264.

**Statement of Significance**

The findings of this work support the hypothesis that early life nutrition is a key factor in the prevention of allergic diseases. Approaches, such as early introduction to potential allergen foods or probiotics, do represent some responsible strategies toward the prevention of allergic diseases, with new implications for the effective control and early prevention of the later development of childhood allergies.

## Introduction

Allergic diseases, such as asthma, allergic rhinitis, and food allergy, are caused by an inappropriate initiation of type 2 immune responses to innocuous environmental antigens [1]. The incidence of allergic diseases has greatly increased worldwide over the past 3 decades, posing a significant public health issue in most developed countries [2]. These diseases affect approximately 433 million people worldwide, according to estimates from the WHO asthma fact sheet [3]. Risk factors include genetics, urbanization, overweight or obesity, exposure to environmental allergens and irritants, and events in early life, including low birth weight, prematurity, infection, and lack of exposure to microbial products [4].

The immune system develops sequentially through a series of coordinated and timed events that begin in fetal life and continue through the early postnatal period [5]. The early diet also influences susceptibility to allergic diseases due to the contribution of dietary components in modulating the immune system. Many specific dietary components, such as eggs, milk, fish, and peanuts, are common allergens. The consumption of these components in infants may elicit a strong immune response. In addition, dietary compounds from solid foods and their derivatives, such as omega-3 PUFA, tryptophan, acetic acid, vitamin A, vitamin D, and dietary fiber, can regulate immune homeostasis through interaction with various receptors, including G protein-coupled receptors and nuclear receptor [1]. Recent evidence indicates that early introduction of allergenic foods may favor natural tolerance to these innocuous antigens, thereby preventing the development of allergies later in life [[6], [7], [8], [9]]. In some cohort studies, fish intake during infancy was found to be negatively correlated with the incidence of allergic diseases in a dose-dependent manner [[10], [11], [12]]. However, contradicting results have been reported in other cohort studies [[13], [14], [15]]. Results from the cohort studies on this topic are often difficult to compare because of differences in the time points of complementary food introduction, the age of outcome evaluation, and the types and consumption doses of complementary food. To date, no comprehensive analyses of the effects of an early diet on the prevalence of allergic diseases later in life are available. Although there are systematic reviews of complementary feeding and allergic diseases, the studies included in the analysis were published ≥5 y ago.

The aim of this paper was to provide a review of the current knowledge on the role of early-life diet factors in the development of allergic diseases based on epidemiologic data and intervention trial data. The diet factors included in this review are limited to those occurring during the complementary food period, which is when an infant is fed transition from exclusive breastfeeding to family foods (usually from 6–23 mo of age) [16]. A systematic literature review approach was applied to identify and collect relevant studies that were published from after year 2000 through May 2023, followed by a meta-analysis to assess evidence that food diversity, introduction of solid and allergenic foods, vitamin D supplementation, probiotic intervention, and fish consumption during the complementary food period influence the risk of allergic disease development.

## Methods

Methods are described in the **Supplemental material**. This systematic review was performed following the PRISMA guidance [17]. The following electronic databases and trial registers were searched from their inception up to 31 May 2023 (articles prior to 2000 were manually reviewed), without restrictions on the language of publication: the Cochrane Library, EMBASE, Web of Science (comprising Web of Science Core Collection, Chinese Science Citation Database, FSTA-the food science resource, KCI-Korean Journal Database, MEDLINE, and SciELO Citation Index), and PubMed. Intervention trials and observational studies involving dietary factors (e.g., dietary patterns, food diversity, introduction of solid and allergenic foods, and consumption of probiotics/fish/vitamin D) during the complementary food period and allergic diseases (food allergy, asthma, and atopic dermatitis [AD]) at any age were included. Details for all search strategies, including search terms, are available in the Supplemental material. Allergic diseases in this review included food allergy [18], asthma/wheezing [19], and AD [20]. Rare manifestations of diseases, such as eosinophilic esophagitis, were specifically excluded because their prevalence is <1‰. The analyses were stratified by timepoint for dietary exposure/intervention onset and outcome assessment, duration of dietary exposure/intervention, type of allergenic foods, and specific foods. For each outcome measure, there was more than one possible method of assessment.

Title and abstract screening were undertaken in duplicate by a team of 4 review authors (SW, PY, LY, and FT). Each 2 reviewers were assigned to a panel, and each panel independently assessed studies. Publication bias was assessed using funnel plots and Egger's test for those meta-analyses with ≥10 studies included [20]. The risk of bias in randomized controlled trial (RCT) studies was assessed using the Cochrane Collaboration Risk of Bias tool [21], and the risk of bias in non-RCT trials was assessed using the Risk of Bias in Nonrandomized Studies of Interventions tool [22]. For all study reports, the summary Tables (Supplemental Tables 1 and 2) [[8], [9], [10], [11], [13], [14], [15], [25], [26], [27], [28], [29], [30], [31], [32], [33], [34], [35], [36], [37], [38], [39], [40], [41], [42], [43], [44], [45], [46], [47], [48], [49], [50], [51], [52], [53], [54], [55], [56], [57], [58], [59], [60], [61], [62], [63], [64], [65], [66], [67], [68], [69], [70], [71], [72], [73], [74], [75], [76], [77], [78], [79], [80], [81], [82], [83], [84], [85], [86], [87], [88], [89], [90], [91]] of study characteristics with key study features and the summary Figures (Supplemental Figures 1–7) showing the risk of bias for all included studies were created. Heterogeneity was quantified using the Higgins inconsistency test (I^2^) [23]. Pooled results for binary outcomes were presented as odds ratio (OR) using the Mantel-Haenszel method (with continuity correction of 0.5 in studies with zero cell frequencies) for pooled OR. Pooled results for continuous outcomes measured using similar scales are presented as mean differences with 95% confidence intervals (CIs). The OR and relative risk (RR) were combined in meta-analysis and plotted as pooled OR, as the majority of cohorts, case-control studies, and cross-sectional studies reported this effect measure using the generic inverse variance method [19]. Forest plots were used to visually assess pooled estimates and corresponding 95% CIs.

Statistical analyses were performed using RevMan 5 (version 5.4.1). The GRADE approach was followed to rate the certainty of evidence (Supplemental Table 3) [24]. This study is registered in PROSPERO with CRD42022379264.

## Results

**FIGURE 1:**
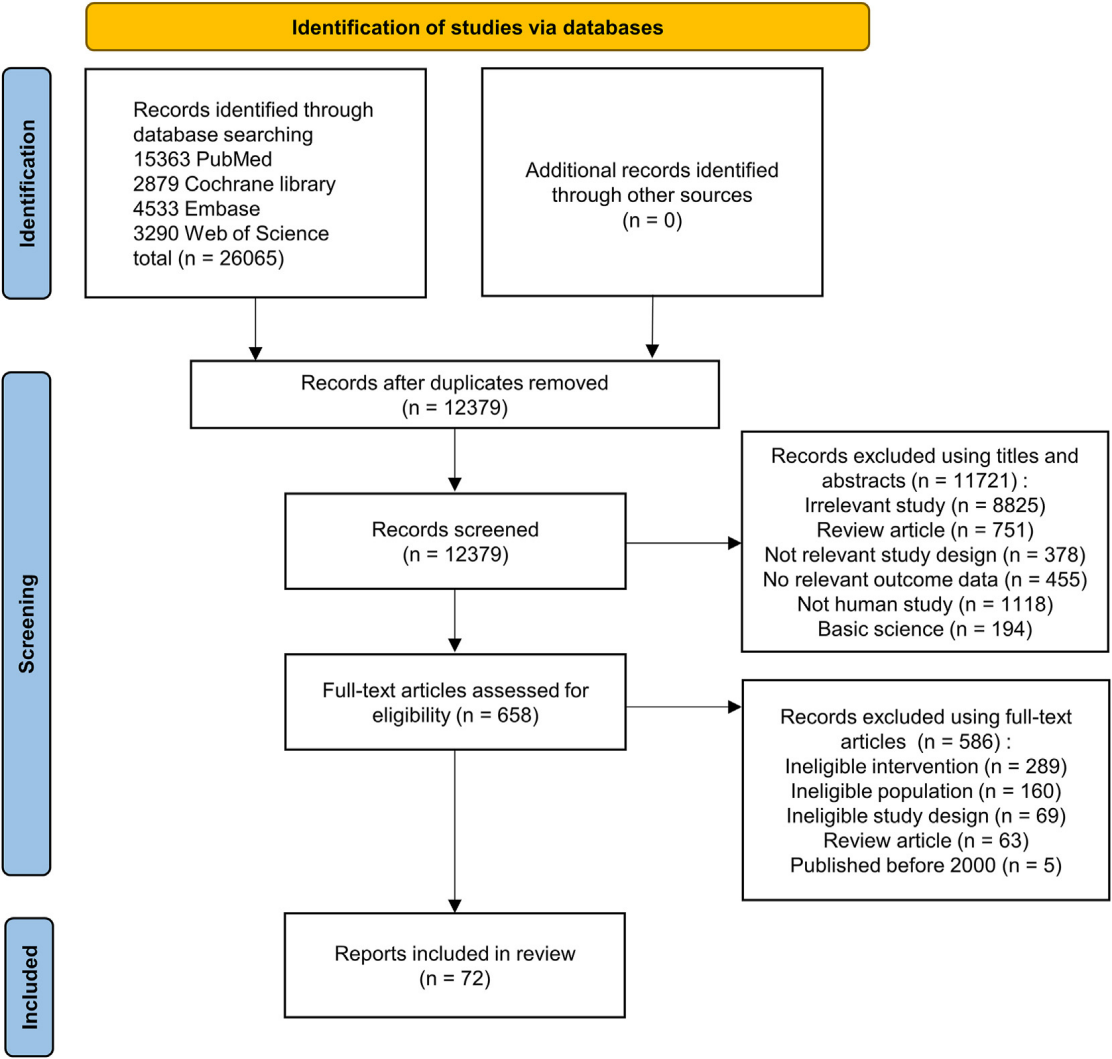
Flowchart of the search and selection processes of records included in this review.

Figure 1 PRISMA flow diagram shows the details of the search protocol used for the selection and exclusion of records. A total of 26,065 records were retrieved from 4 databases (PubMed, Cochrane Library, Embase, and Web of Science). After removing duplicates, 12,379 records were screened based on titles and abstracts. Following the title and abstract review, 11,721 records were excluded. Six hundred fifty-eight full-text articles were evaluated to determine whether they were eligible. In particular, 5 articles were published before the year 2000 and were excluded manually. A total of 72 records met the inclusion criteria.

Finally, 54 eligible studies (72 separate titles) were identified. Overall, 10 intervention trials (17 titles) evaluated allergic outcomes in 6208 participants, and 44 observational studies (55 titles) reported allergic outcomes in 167,795 participants. The intervention studies included cluster-randomized studies (*n* = 2) and RCT studies (*n* = 15). Observational studies included 47 prospective cohort studies, 3 nested case-control studies, 3 case-control studies, 1 cross-sectional study, and 1 retrospective cohort study. Characteristics of included studies are summarized in Supplemental Tables 1 and 2.

Risk of bias was low in 8 (48%) of 17 intervention studies and 25 (45%) of 55 observational studies (Supplemental Figures 4 and 6). The main issues identified were selection bias in intervention studies and missing data in observational studies. The GRADE evidence assessment was summarized in Supplemental Table 3.

## Risk of food allergy

### Early introduction of complementary foods

**FIGURE 2:**
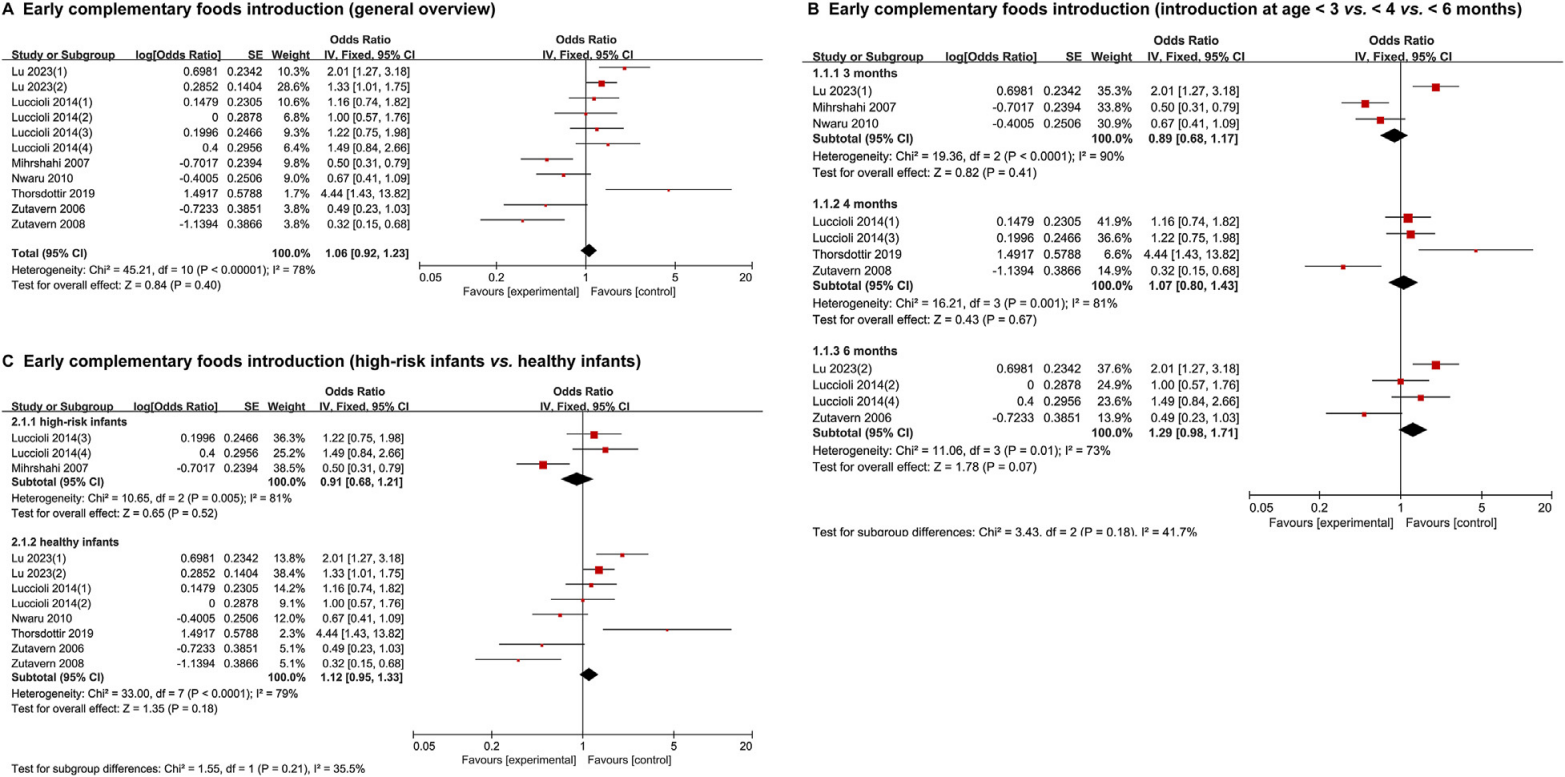
Effect of early compared with late intake of complementary foods on risk of food allergy. Data are from prospective cohorts. Effects on all participants (A) [[15], [43], [52], [59], [68], [81], [83]]. Effect of specific timing of intake (B) [[15], [43], [52], [59], [68], [81], [83]] and Effects on infants at high/normal risk of allergy (C) [[15], [43], [52], [59], [68], [81], [83]].

Meta-analysis of 7 studies (*n* = 10,479 participants) revealed no significant association between the timing of complementary foods introduction and food allergy (OR, 1.06; 95% CI: 0.92, 1.23; *P* = 0.40; high heterogeneity [I^2^ = 78%], Figure 2A) [15,43,52,59,68,81,83]. Subgroup analyses of the time points (<3 compared with <4 compared with <6 mo) (Figure 2B) [[15], [43], [52], [59], [68], [81], [83]] of complementary food introduction showed that early introduction of complementary foods did not reduce the risk of food allergy in the future. A similar observation was noted when high-risk food allergy and general population cohorts (1589 participants compared with 8890 participants) were analyzed separately (Figure 2C) [[15], [43], [52], [59], [68], [81], [83]].

### Early introduction of allergenic foods

**FIGURE 3:**
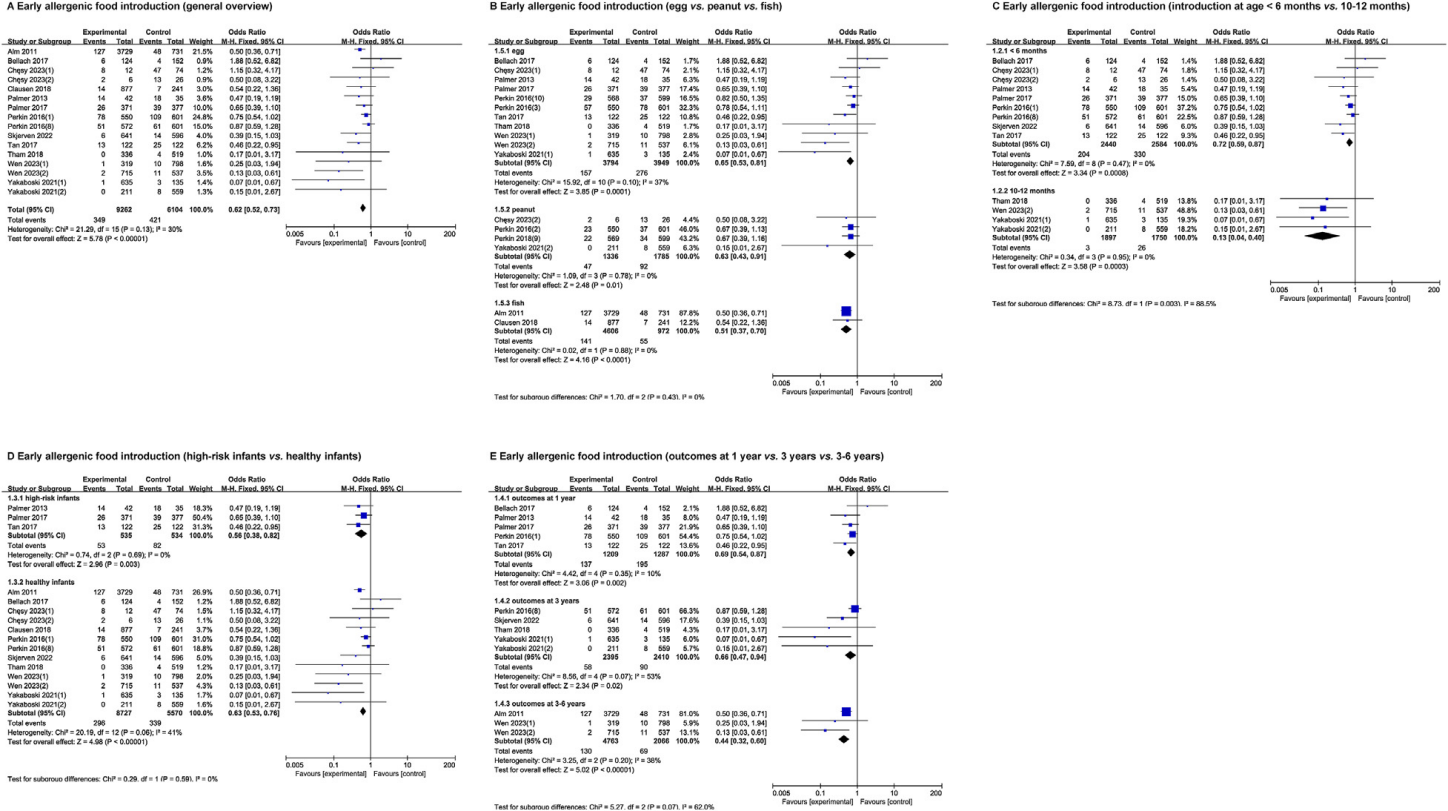
Effect of early compared with late intake of allergenic foods on risk of food allergy. Data are from 6 intervention trials and 6 observational cohorts. Effects on all participants (A) [[8], [9], [10], [13], [14], [25], [35], [36], [41], [44], [47], [72]]. Effects of specific allergenic food (B) [[8], [9], [10], [13], [14], [25], [35], [36], [41], [44], [47], [72]] and specific timing of intake (C) [[8], [9], [13], [14], [25], [35], [36], [41], [44], [47]]. Effects on infants at high/normal risk of allergy (D) [[8], [9], [10], [13], [14], [25], [35], [36], [41], [44], [47], [72]] and outcome assessment (E) [[8], [9], [13], [14], [25], [35], [36], [44], [47], [72]].

Meta-analysis of 6 intervention studies and 6 observational studies (*n* = 14,439 participants) showed a lower risk of food allergy with early introduction of allergenic foods compared with later introduction (OR, 0.62; 95% CI: 0.52, 0.73; *P* < 0.00001; moderate heterogeneity [I^2^ = 30%], Figure 3A) [8,9,10,13,14,25,35,36,41,44,47,72].

Eight major food allergens, also known as the “Big 8,” are milk, eggs, fish, crustacean shellfish, tree nuts, peanuts, wheat, and soybean (the US Food Allergen Labeling and Consumer Protection Act of 2004). Subgroup analyses of common food antigens showed that early introduction of eggs, peanuts, or fish was associated with a significantly lower prevalence of food allergy (Figure 3B) [[8], [9], [10], [13], [14], [25], [35], [36], [41], [44], [47], [72]]. Perkin et al. (2016) [9] demonstrated that both the prevalence of overall food allergy and that of allergy to peanuts or eggs in 1303 exclusively breast-fed, 3-mo-old infants were negatively correlated with allergenic foods intake in a dose-dependent manner.

The addition of allergenic foods in the diet of infants <6 mo was a protective factor against future food allergy risk compared with infants >6 mo (OR, 0.72; 95% CI: 0.59, 0.87; *P* = 0.0008; low heterogeneity [I^2^ = 0%], Figure 3C) [[8], [9], [13], [14], [25], [35], [36], [41], [44], [47]]. Allergenic foods introduction at age 10–12 mo was associated with a lower risk of food allergy than introduction at age >12 mo (OR, 0.13; 95% CI: 0.04, 0.40; *P* = 0.0003; low heterogeneity [I^2^ = 0%], Figure 3C). The early introduction of allergenic foods significantly reduced the incidence of food allergy in both allergy-risk and normal-risk infants (Figure 3D) [[8], [9], [10], [13], [14], [25], [35], [36], [41], [44], [47], [72]]. Similar results were obtained in the meta-analysis of trials with an outcome end point of 1, 3, or 3–6 y of age (Figure 3E) [[8], [9], [13], [14], [25], [35], [36], [44], [47], [72]].

### Probiotic supplementation during the complementary food period

**FIGURE 4:**
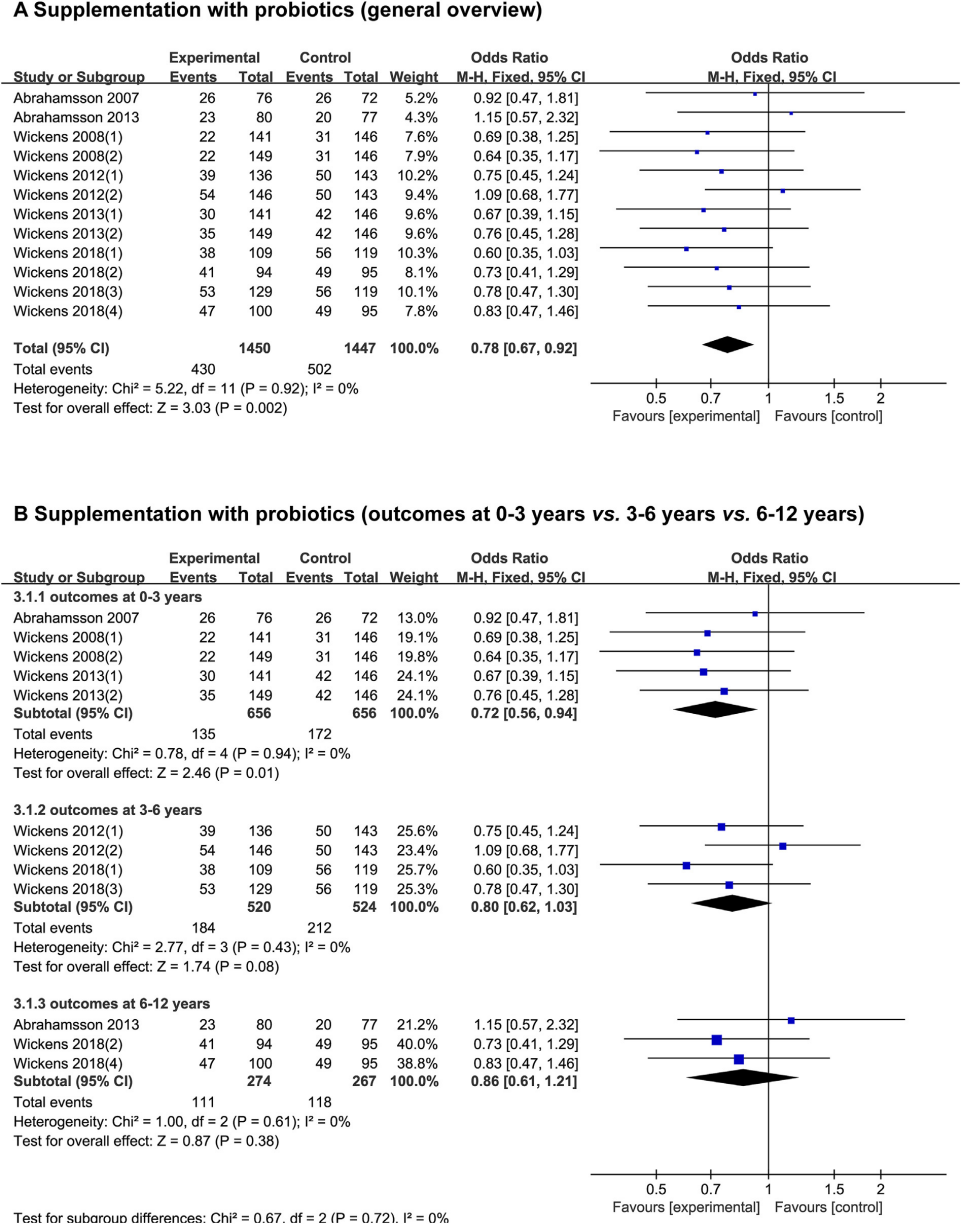
Effect of probiotic supplementation during the complementary food period on risk of food allergy. Effects on all participants (A) [[31], [32], [33], [34], [39], [40]] and time for outcome assessment (B) [[31], [32], [33], [34], [39], [40]].

Six randomized, double-blinded, and placebo-controlled studies reported the effect of probiotic supplementation on food allergy in 2897 participants at high risk of food allergy. Of these studies, 4 used *Bifidobacterium lactis* HN019 [[31], [32], [33], [34]], 4 used *Lacticaseibacillus rhamnosus* HN001 [[31], [32], [33], [34]], and 2 used *Limosilactobacillus reuteri* ATCC5573 [39,40]. Overall, a meta-analysis revealed that probiotic supplementation during the complementary food period had a significant beneficial effect on the future risk of food allergy (OR, 0.78; 95% CI: 0.67, 0.92; *P* = 0.002; low heterogeneity [I^2^ = 0%], Figure 4A) [[31], [32], [33], [34], [39], [40]].

Figure 4B [[31], [32], [33], [34], [39], [40]] shows the pooled ORs for probiotic supplementation in the prevention of food allergies in predefined subgroups. The results were not consistent among the subgroups. In particular, probiotic intervention within the first 2 y of life significantly reduced food allergies in children aged 0–3 y (OR, 0.72; 95% CI: 0.56, 0.94; *P* = 0.01; low heterogeneity [I^2^ = 0%]). However, no significant protective effect was observed for preschoolers aged 3–6 y (*P* = 0.08) and school-aged (6–12 y) children (*P* = 0.38).

For healthy infants, a systematic review of 1 randomized, double-blinded, and placebo-controlled study and one prospective cohort study (total of 8578 participants) demonstrated no significant association between probiotic supplementation during complementary food period and the development of food allergies in childhood [46,37]. The investigated probiotics included *Bifidobacterium breve* BC50, *Bifidobacterium lactis* BB12, *Limosilactobacillus fermentum* CECT5716, *Limosilactobacillus reuteri* DSM 17938, and *Lacticaseibacillus paracasei* F19.

### Fish consumption

**FIGURE 5:**
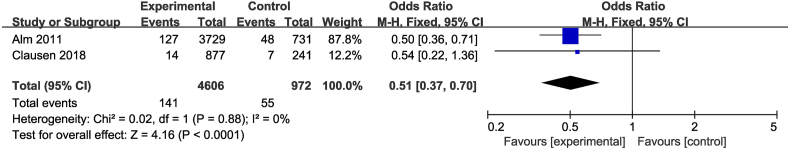
Effect of fish consumption on risk of food allergy [[10], [72]].

A meta-analysis of 2 prospective cohort trials (5578 participants) showed that fish introduction <9 mo of age reduced the risk of food allergy in preschool children (OR, 0.51; 95% CI: 0.37, 0.70; *P* < 0.0001; low heterogeneity [I^2^ = 0%]), as shown in Figure 5 [10,72]. Clausen et al. (2018) [10] also showed that as fish intake increased, the indicators of allergy severity decreased (*P* = 0.013). An association between regular fish consumption and a decreased risk of food allergy at age 4 y was observed using a cohort of 4089 newborns [85].

### Vitamin D supplementation

**FIGURE 6:**
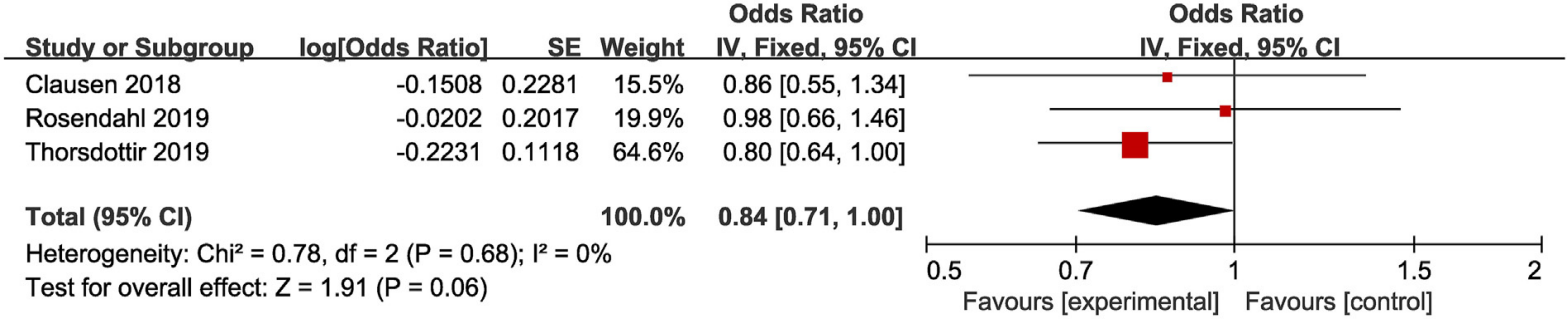
Effect of high-dose vitamin D supplementation on risk of food allergy [[10], [30], [52]].

A meta-analysis of 1 intervention study and 2 observational studies (total of 2423 participants) revealed that there was no association between high-dose vitamin D supplementation and risk of food allergy at the age of 12 mo (OR, 0.84; 95% CI: 0.71, 1.00; *P*  = 0.06; low heterogeneity [I^2^ = 0%]), as shown in Figure 6 [10,30,52].

### Diversity in diet or dietary patterns

A systematic review of 4 prospective cohort studies (total of 5692 participants) showed that increased dietary diversity in infancy reduced the likelihood of developing food allergies in childhood [41,51,58,66]. In addition, a nested-control study (123 participants) investigated the link between infant dietary patterns in the first year of life and the development of food allergy by 2 y of age, which demonstrated that control infants scored significantly higher on a “healthy dietary pattern” characterized by a higher intake of fruits, vegetables, and home-prepared foods compared with children with food allergies [69].

## Risk of asthma

### Early introduction of complementary foods

A meta-analysis of 9 studies (55,302 participants) showed no significant association between the timing of complementary foods introduction and asthma (OR, 0.98; 95% CI: 0.92, 1.04; *P* = 0.52; moderate heterogeneity [I^2^ = 31%], Supplemental Figure 8A) [45,48,55,65,79,[81], [82], [83],^89^]. Subgroup analyses of the time points (<3 mo compared with <4 mo compared with <6 mo) (Supplemental Figure 8B) [[45], [48], [55], [65], [79], [81], [82], [83], [89]] of complementary food introduction or the end points (0–3 compared with 3–6 compared with 6–12 y) of the outcome assessment (Supplemental Figure 8C) [[48], [55], [65], [79], [81], [82], [83], [89]] showed that early introduction of complementary foods was not a protective factor for reduced risk of asthma in the future.

### Early introduction of allergenic foods

A meta-analysis of six prospective cohort studies (total of 28101 participants) showed a lower risk of asthma with early introduction of allergenic foods compared to later introduction (OR, 0.89; 95%CI, 0.84–0.95; *P* = 0.0004; high heterogeneity [I^2^ = 60%], Supplemental Figure 9A) [53,58,60,71,75,89].

Subgroup analyses of common food antigens showed that early introduction of fish reduced the risk of asthma in the future (OR, 0.68; 95% CI: 0.59, 0.78; *P* < 0.00001; high heterogeneity [I^2^ = 55%]), but no such protective effect was observed for eggs or milk (Supplemental Figure 9B) [[53], [58], [60], [71], [75], [89]]. In addition, 3 studies suggested that the timing of the introduction of fruits, vegetables, cereals, and meat did not appear to have a substantial effect on the long-term risk of developing asthma in children [53,58,89].

The results shown in Supplemental Figure 9C [[53], [58], [60], [71], [75], [89]] do not support the early introduction of allergenic foods (eggs, milk, fish, and peanuts) <6 mo of age to prevent asthma. However, during 6 and 12 mo of age, the early introduction of allergenic foods reduced the risk of childhood asthma compared with the delayed introduction (6–8 mo compared with >8 mo, OR, 0.73; 95% CI: 0.62, 0.86; *P* = 0.0001; high heterogeneity [I^2^ = 60%]; 8–12 mo compared with >12 mo, OR, 0.69; 95% CI: 0.61, 0.79; *P* < 0.00001; high heterogeneity [I^2^ = 51%]). Subgroup analyses of the timing of outcome assessment showed that early introduction of allergenic foods reduced the risk of asthma in children aged ≥3 y; however, no significant protective effect was observed in children aged 0–3 y (Supplemental Figure 9D) [[11], [53], [58], [60], [71], [75], [85], [89], [90]].

### Probiotic supplementation during the complementary food period

Five randomized, double-blinded, placebo-controlled studies reported asthma in 2596 participants with a high risk of asthma. Of these studies, 3 trials used *B. lactis* HN019 [[31], [32], [33]], 4 used *L. rhamnosus* HN001 [[31], [32], [33]], and 2 used *L. reuteri* ATCC5573 [39,40]. Overall, a meta-analysis showed that probiotic supplementation during the complementary food period had a significant beneficial effect on the risk of asthma (OR, 0.82; 95% CI: 0.68, 0.99; *P* = 0.04; low heterogeneity [I^2^ = 0%], Supplemental Figure 10A) [[31], [32], [33], [39], [40]]. In addition, Wickens et al. (2012; 2013; 2018) [[31], [32], [33]] showed that *L. rhamnosus* HN001 supplementation in infants from birth until 2 y reduced cumulative prevalence of wheezing at ages 4, 6, and 11 y, whereas *B. animalis* subsp. *lactis* HN019 had no significant effect at any time.

Supplemental Figure 10B [[31], [32], [33], [39], [40]] shows the pooled ORs for probiotic supplementation for asthma prevention in predefined subgroups. The results were not consistent among the subgroups. More particular, probiotic intervention within the first 2 y of life significantly reduced asthma in children aged 6–12 y (OR, 0.61; 95% CI: 0.41, 0.90; *P* = 0.01; low heterogeneity [I^2^ = 0%]); however, no significant protective effect was observed in children aged 0–6 y (0–3 y: *P* = 0.33, 3–6 y: *P* = 0.41).

For healthy infants, 2 randomized, double-blinded, and placebo-controlled studies and 1 prospective cohort study (total 8858 participants) reported the effect of probiotic supplementation during complementary food period on asthma in childhood [29,37,46]. These 2 intervention studies using *L. paracasei* F19, *L. rhamnosus* (LGG), or *B. animalis* subsp *lactis* (BB12) demonstrated no beneficial effect of probiotic supplementation on asthma [29,37]. The controversial results were shown in that prospective cohort study (8389 participants): The consumption of *B. breve* was associated with a lower risk of asthma, whereas the consumption of *Streptococcus thermophilus* was associated with a higher risk of asthma [46].

### Fish consumption

A meta-analysis of 5 prospective cohort trials (17,521 participants) revealed that the frequency of fish intake in infancy was associated with the risk of asthma in the future (OR, 0.65; 95% CI: 0.59, 0.73; *P* < 0.00001; moderate heterogeneity [I^2^ = 32%], Supplemental Figure 11A) [11,49,71,85,90]. Supplemental Figure 11B [[11], [71], [85], [90]] shows the pooled ORs for fish consumption in asthma prevention in the predefined subgroups. Fish consumption (≥1 time/mo) during the first year of life appeared to be associated with a reduced risk of asthma compared with no fish consumption (OR, 0.63; 95% CI: 0.56, 0.71; *P* < 0.00001; moderate heterogeneity [I^2^ = 28%]). Fish consumption was classified as irregular (≤1 time/mo) or regular (≥2–3 times/mo), and children who consumed fish regularly during the first year of life had an overall reduced risk of asthma (OR, 0.75; 95% CI: 0.62, 0.91; *P* = 0.003; low heterogeneity [I^2^ = 17%]). Magnusson et al. (2013) [11] indicated that children who consumed fish at 1 y of age had an overall reduced risk of prevalent asthma by the age of 12 y, and reduced risks were dose-dependent for the outcomes (*P* < 0.001).

Supplemental Figure 11C [[53], [58], [60], [71], [89]] shows that early fish introduction in infancy may reduce the risk of asthma in children compared with later introduction (OR, 0.68; 95% CI: 0.59, 0.78; *P* < 0.00001; high heterogeneity [I^2^ = 55%]). In particular, children who started consuming fish at 6–12 mo had a significantly reduced risk of asthma in the future than children who started consuming fish at ≥ 12 mo (OR, 0.61; 95% CI: 0.52, 0.72; *P* < 0.00001; low heterogeneity [I^2^ = 24%], Supplemental Figure 11D) [[53], [58], [60], [71], [89]].

In addition, after stratification by the age of outcome assessment (3–6 y, 6–10 y, and >10 y), no significant differences were found between the strata, which indicated a long-term effect of fish consumption in infancy on the subsequent development of asthma (Supplemental Figure 11E) [[11], [53], [58], [60], [71], [85], [89], [90]].

### Vitamin D supplementation

In Supplemental Figure 12 [[30], [56], [80], [88]], a meta-analysis of 1 intervention study and 3 observational studies (total of 7526 participants) showed no evidence that high-dose vitamin D supplementation in the first year of life was protective against asthma in the future (OR, 1.17; 95% CI: 0.95, 1.46; *P* = 0.14; low heterogeneity [I^2^ = 17%]) [30,56,80,88].

### Diversity of diet or dietary patterns

Four prospective cohort studies (total of 13,815 participants) were found that examined the association between dietary diversity in infancy and the development of asthma in childhood [57,58,66,76]. Of these studies, 3 prospective cohort studies have shown that increased dietary diversity in the first year of life reduced the likelihood of developing asthma [57,58,66]. However, another prospective cohort study did not support the early intake of highly diverse solid foods [76].

A systematic review of 3 prospective cohort studies (total of 13,261 participants) showed the relationship between dietary patterns during infancy and childhood asthma [50,54,74]. Excessive unbalanced meat consumption characterized by daily meat consumption and rare consumption of milk and yogurt [50] or high adherence to the “Western” dietary pattern [74] increased the risk of asthma/wheezing in childhood, whereas the dietary pattern rich in noodles and seafood was associated with reduced odds of wheezing and use of nebulizer/inhaler in childhood [54].

## Risk of AD

### Early introduction of complementary foods

Eleven prospective cohort studies (41,637 participants) [15,42,48,77,[81], [82], [83], [84],^87^,^89^,^91^], 2 case-control studies (1142 participants) [62,86], 1 nested case-control study (1128 participants) [78], 1 cross-sectional study (760 participants) [65], and 1 cluster-randomized study (1239 participants) [26] reported on AD. Overall, in both intervention and observational studies, early complementary food introduction did not appear to be associated with the risk of AD (OR, 0.94; 95% CI: 0.88, 1.02; *P* = 0.13; moderate heterogeneity [I^2^ = 47%]), shown in Supplemental Figure 13A. [[15], [26], [42], [48], [62], [65], [77], [78], [81], [82], [83], [84], [86], [87], [89], [91]] A similar result was observed in the subgroup after stratification according to the timing of complementary food introduction (Supplemental Figure 13B) [[15], [26], [42], [48], [62], [65], [77], [78], [81], [82], [83], [84], [86], [87], [89], [91]]. In addition, the results showed that the early introduction of complementary foods had no protective effect at any age after stratification (Supplemental Figure 13C) [[15], [26], [42], [48], [62], [65], [77], [78], [81], [82], [83], [84], [86], [87], [89], [91]]. Meta-analysis of cohorts of high-risk AD and the general population also confirmed that early complementary food introduction and risk of AD are not associated (Supplemental Figure 13D) [[15], [26], [42], [48], [62], [65], [77], [78], [81], [82], [83], [84], [86], [87], [89], [91]].

### Early introduction of allergenic foods

Meta-analysis of 3 intervention studies and 12 observational studies (total of 37,824 participants) revealed that early allergenic foods introduction was associated with a lower risk of AD (OR, 0.88; 95% CI: 0.85, 0.92; *P* < 0.00001; moderate heterogeneity [I^2^ = 34%]), shown in Supplemental Figure 14A [8,14,15,25,42,64,67,70,61,73,75,78,84,87,89].

Subgroup analyses of common food antigens indicated that the early introduction of milk or fish was correlated with a significantly lower prevalence of AD, whereas the association between early introduction of eggs or peanuts and reduced AD was not statistically significant (Supplemental Figure 14B) [[8], [14], [15], [42], [61], [64], [67], [70], [73], [75], [78], [84], [87], [89]]. Five studies also showed that the timing of the introduction of fruits, vegetables, cereals, and meat does not influence the risk of AD in children [15,70,78,84,89].

Introduction of allergenic foods in infants <6 mo of age was a protective factor for risk of AD than that beyond 6 mo of age (OR, 0.93; 95% CI: 0.89, 0.97; *P* = 0.002; low heterogeneity [I^2^ = 0%]), as shown in Supplemental Figure 14C [[8], [14], [15], [25], [42], [61], [64], [67], [70], [73], [75], [78], [84], [87], [89]]. Compared with >12 mo of age, the risk of AD was also reduced by allergenic foods introduction in infants aged 8–12 mo (OR, 0.76; 95% CI: 0.67, 0.86; *P* < 0.0001; extreme heterogeneity [I^2^ = 78%]). The addition of allergenic foods at 6–8 mo (compared with >8 mo) did not exhibit such protective effects (OR, 0.99; 95% CI: 0.69, 1.42; *P* = 0.96; low heterogeneity [I^2^ = 0%]). Furthermore, the early addition of allergenic foods in infancy significantly reduced the incidence of AD in children under 3 y of age (OR, 0.85; 95% CI: 0.81, 0.90; *P* < 0.00001; moderate heterogeneity [I^2^ = 36%]), as shown in Supplemental Figure 14D. [[8], [14], [15], [25], [42], [61], [64], [67], [70], [73], [75], [78], [84], [87], [89]] The association between early allergenic foods introduction and a reduced risk of AD in children aged 3–6 y or ≥10 y was not statistically significant.

### Probiotic supplementation during the complementary food period

Three randomized, double-blinded, placebo-controlled trials (7 reports) reported AD in 808 participants at a high risk of AD. Of these, 4 used *B. lactis* HN019 [[31], [32], [33], [34]], 4 used *L. rhamnosus* HN001 [[31], [32], [33], [34]], 2 used *L. reuteri* ATCC5573 [39,40], and 1 report used *L. paracasei* F19 [38]. Overall, the meta-analysis showed the significant beneficial effect of probiotic supplementation for 1 or 2 y during the complementary food period on the risk of AD in the future (OR, 0.72; 95% CI: 0.62, 0.85; *P* < 0.0001; low heterogeneity [I^2^ = 17%]), shown in Supplemental Figure 15A [[31], [32], [33], [34], [38], [39], [40]]. Wickens et al. (2008; 2013; 2018) [31,32,34] showed that *L. rhamnosus* HN001 supplementation in infancy reduced the cumulative prevalence of AD at ages 2, 6, and 11 y, whereas *B. animalis* subsp. *lactis* HN019 had no significant effect at any time.

Supplemental Figure 15B [[31], [32], [33], [34], [38], [39], [40]] shows the pooled ORs for probiotic supplementation in the prevention of AD in predefined subgroups. The results were not consistent among the subgroups. More specifically, probiotic intervention significantly reduced the risk of AD in children aged <6 y (0–3 y: OR, 0.70; 95% CI: 0.52, 0.94; *P* = 0.02; high heterogeneity [I^2^ = 55%]; 3–6 y: OR, 0.73; 95% CI: 0.59, 0.91; *P* = 0.005; low heterogeneity [I^2^ = 0%]); however, no significant protective effect was observed in children aged 6–12 y (OR, 0.74; 95% CI: 0.50, 1.11; *P* = 0.14; high heterogeneity [I^2^ = 56%]).

For healthy infants, 2 randomized, double-blinded, and placebo-controlled studies and 1 prospective cohort study (total 8868 participants) were found that examined the effect of probiotic supplementation during complementary food period on eczema in childhood [29,37,46]. The prospective cohort study investigating *B. breve* BC50, *B. lactis* BB12, *L. fermentum* SM, *L. reuteri* DSM 17938, or *S. thermophilus* [46] and the intervention study using *L. paracasei* F19 [37] confirmed that probiotics were not significantly associated with the risk of itchy rash/eczema. However, the intervention study using LGG and BB12 showed that probiotics significantly reduced the incidence of eczema in children (4.2% compared with 11.5%; *P* = 0.036) [29].

### Fish consumption

Eleven prospective cohort trials [11,15,42,49,67,70,73,84,85,87,89] and one nested case-control study [78] (total of 27,399 participants) reported the association between fish consumption in the first year of life and risk of AD. The results of the meta-analysis showed that infants who consumed fish ≤ 1 y of age had a lower risk of AD in childhood than those who did not (OR, 0.56; 95% CI: 0.53, 0.60; *P* < 0.00001; extreme heterogeneity [I^2^ = 81%]), shown in Supplemental Figure 16A [[11], [42], [49], [73], [85], [87]]. Furthermore, Magnusson et al. (2013) [11] demonstrated that children who consumed fish at 1 y of age had an overall reduced risk of eczema by age 12 y, and reduced risks were dose-dependent for all outcomes (1 time/mo: OR [95% CI] = 0.59 [0.46, 0.76]; 2–3 times/mo: OR [95% CI] = 0.58 [0.47, 0.72]; 1 time/wk: OR [95% CI] = 0.44 [0.36, 0.54]; >1 time/wk: OR [95% CI] = 0.43 [0.35, 0.54], never as reference, *P* < 0.001) (11). Similarly, Kull et al. (2006) [85] also confirmed that children who consumed fish during the first year of life had a reduced risk of eczema at age 4, and a dose-dependent reduced risk was observed for the outcome (1 time/mo: OR [95% CI] = 0.72 [0.51, 1.00]; 2–3 times/mo: OR [95% CI] = 0.71 [0.53, 0.95]; 1 time/wk: OR [95% CI] = 0.54 [0.41, 0.70]; ≥1 time/wk: OR [95% CI] = 0.57 [0.43, 0.76], never as a reference, *P* < 0.001). Alm et al. (2009) [73] showed that children who consumed fish regularly (1–3 times/mo) had a lower overall risk of eczema prevalence than those who consumed fish irregularly (a few times a year; 57/213 compared with 247/1285) [73].

Supplemental Figure 16B [[15], [42], [67], [70], [73], [78], [84], [89]] shows that early introduction of fish in infancy may reduce the risk of AD in children compared with the later fish introduction (OR, 0.77; 95% CI: 0.70, 0.84; *P* < 0.00001; moderate heterogeneity [I^2^ = 46%]). In particular, children receiving fish <9 mo of age had a significantly reduced risk of AD than children introduced to fish >9 mo (OR, 0.63; 95% CI: 0.53, 0.74; *P* < 0.00001; low heterogeneity [I^2^ = 0%]), as shown in Supplemental Figure 16C. [[15], [42], [67], [70], [73], [78], [84], [89]] However, fish consumption in infants <4 or 6 mo did not reduce the incidence of AD in their childhood (<4 mo: OR, 1.54; 95% CI: 0.73, 3.23; *P* = 0.26; low heterogeneity [I^2^ = 0%]; <6 mo: OR, 1.02; 95% CI: 0.90, 1.15; *P* = 0.78; low heterogeneity [I^2^ = 0%]), as shown in Supplemental Figure 16C.

In addition, no significant differences were found between strata by stratification assessment of the age of asthma patients (0–3 y, 3–6 y, and 6–12 y), suggesting a long-term beneficial effect of fish consumption within the first year of life on the subsequent development of AD (Supplemental Figure 16D) [[11], [42], [49], [73], [85], [87]]. The beneficial effect of fish supplementation <9 mo only reduced the incidence of AD in children before the age of 3 y (Supplemental Figure 16E) [[15], [42], [67], [70], [73], [78], [84], [89]].

### Vitamin D supplementation

As shown in Supplemental Figure 17, [[30], [80]] a meta-analysis of 1 intervention trial and 1 observational trial (a total of 1098 participants) showed that high-dose vitamin D supplementation in the first year of life did not protect against AD (OR, 0.94; 95% CI: 0.66, 1.33; *P* = 0.71; extreme heterogeneity [I^2^ = 90%]) [30,80]. The extreme heterogeneity was due to differences in the period and method of outcome assessment, as well as the criteria for high and low vitamin D doses.

### Diversity of diet or dietary patterns

A systematic review of 4 prospective cohort studies (total of 15,604 participants) showed the relationship between solid food diversity in infants and AD in children [76,84,89,91]. Overall, 4 studies suggested that early intake of a large amount of solid food was not beneficial for AD/eczema prevention. Even the introduction of a high diversity of solid ≤ 4 mo of age might increase the risk of eczema/AD [76,89]. In addition, a prospective cohort study (1152 participants) examined the infant dietary patterns in the first year of life on eczema in childhood and showed the “easy to prepare food” dietary pattern which consists of infant cereals, juices, cake, and biscuits (at 9 mo of age) or “predominantly breastmilk” dietary pattern (at 12 mo of age) was associated with increased odds of developing eczema by the age 18 mo [54].

## Discussion

This systematic review found that the introduction and diversity of complementary foods, particularly specific foods (e.g., allergenic foods, fish, and probiotics), were related to the risk of allergic disease in the future. Low-quality evidence suggests that the early introduction of allergenic foods may reduce the risk of food allergy, asthma, and AD. High-quality evidence suggests that probiotic supplementation in infants with a high risk of developing allergic diseases may reduce food allergies (3 y or younger), asthma (6–12 y), and AD (6 y or younger) in the future. In addition, low to moderate-quality evidence revealed that regular fish supplementation in the first year of life was significantly effective in preventing food allergies (moderate), asthma (low), and AD (low), and indicators of severity decreased with increased fish consumption. Fish introduction before 9 mo of age reduced food allergies, and AD in childhood, and the introduction of fish at 6–12 mo was associated with a reduction in asthma. Increased diversity of complementary foods was negatively associated with food allergies (low-quality evidence) and asthma (moderate-quality evidence) but may have no effect on the incidence of AD (low-quality evidence).

Ierodiakonou et al. (2016) [19] assessed the association between the timing of allergenic foods introduction and the risk of allergic disease based on intervention and observational studies. The results showed that the addition of eggs at 4–6 mo was associated with reduced egg allergy; the addition of peanuts at 4–11 mo was associated with reduced peanut allergy; and the early addition of fish was associated with reduced allergic sensitization and rhinitis. Obbagy et al. (2019) [92] confirmed that the timing at which complementary feeding begins is not related to the risk of developing food allergy, AD/eczema, or childhood asthma.

The gastrointestinal tract is the largest immune organ in the body. Within the mucosa-associated lymphoid tissue, unique populations of dendritic cells interact with dietary antigens and determine the fate of the resulting adaptive response (immunity compared with tolerance). When food allergens are initially encountered through the gut, a robust T cell-mediated suppression develops called oral tolerance [93]. In a word, the suppression involves signaling by an array of nonprofessional antigen-presenting cells, dendritic cells, and regulatory T cells, as well as lymphocyte anergy or deletion [94].

Overall, this review found that the timing of the first introduction of solid foods was not associated with the risk of developing food allergies, asthma, or AD during childhood. Although 2 studies [81,83] showed that the early introduction of complementary foods reduced the risk of developing food allergies, the accuracy was limited by the fact that these studies were not RCTs that had some potential confounding factors and were conducted 2 decades ago. In addition, the preponderance of evidence has indicated that the timing at which complementary foods were consumed is not associated with food allergies, asthma, or AD risk. Diet diversity is defined as the number of foods or food groups consumed over a given period. Interest has been increasingly focused on the effects of infant dietary diversity in preventing allergic diseases. A task force report from the European Academy of Asthma, Allergy, and Immunology suggested that increased dietary diversity may be associated with reduced risk of developing allergic disease via the effect on the microbiome, increased intake of nutrients related to allergy prevention, and increased exposure to allergens [95]. Few children receive nutritionally adequate and safe complementary foods. The WHO reported (2021) that in many countries, less than a fourth of infants aged 6 through 23 mo meet the criteria of dietary diversity and feeding frequency that are appropriate for their age.

Regular intake of fish during infancy is associated with a reduced risk of developing food allergies, asthma, or AD. The timing of fish supplementation seems to be important, as some studies have shown that early intake of fish oil provides greater protection. A plausible biological mechanism is that fish contain omega-3 PUFAs (n–3 PUFA), which exhibit anti-inflammatory properties and may help prevent allergic sensitization and other inflammatory conditions [96]. The anti-inflammatory effects of n–3 PUFAs are caused by their influence on cell membrane structure, cell signaling, and antigen presentation of antigens [10].

The hygiene hypothesis suggests that reduced exposure to microbial stimuli early in life leads to an increased prevalence of atopic diseases [97]. In this respect, a possible mechanism of the potential role of probiotics in the development of atopic disease is the modulation of the immune system, including improving the balance of infant Th1/Th2 immune responses. Probiotics intake may skew immune responses toward immunoregulation by inducing Treg cells rather than eliciting a proinflammatory immune response [98]. The relative abundance of the beneficial genera (*Bifidobacterium* and *Lactobacillus*) was lower in the gut during infancy of allergic children than in nonallergic children, supporting the effects of probiotic role may be a strategy for preventing infants from atopic disease in childhood [99]. Interestingly, for different allergic diseases, it was found that probiotic supplementation during infancy had different protective effects in children of various ages. Probiotic supplementation reduced the prevalence of food allergy in children aged ≤ 3 y, that of asthma was 6–12 y, and of AD was 0–6 y. This may be because food allergies and AD easily develop in early childhood; however, asthma usually does not develop until later in childhood.

Vitamin D plays a role in calcium and phosphate homeostasis. However, little is known about the effect of vitamin D on the development of allergic diseases. Although studies have shown that serum vitamin D levels are negatively associated with the risk of food allergy [100], asthma [101], and AD [102]. In this research, high-dose vitamin D intake was not observed to prevent the development of allergic diseases. However, studies analyzing the association between high-dose vitamin D and allergic diseases are scarce; therefore, further clinical trials are needed to provide conclusive evidence and to determine whether vitamin D affects the development of allergic diseases.

WHO guidelines recommend that: “Around the age of 6 mo, an infant’s need for energy and nutrients starts to exceed what is provided by breast milk, and complementary foods are necessary to meet those needs. An infant of this age is also developmentally ready for other foods.” The study suggests that complementary foods or fortified complementary foods and specific supplements may be an effective strategy during the complementary feeding window for preventing the risk of developing allergic diseases in the future.

This analysis has several advantages. First, a broad search strategy was used in electronic databases and trial registries without limiting the date or language, although articles published before 2000 were excluded manually. Second, the interventional and observational studies were included to obtain comprehensive and relevant studies, which were unlikely to miss published trials, except for unpublished or ongoing trials that were not registered in the clinical trial registry. In addition, to minimize bias, at least 2 review authors performed independently on trial selection, data extraction, risk of bias, and GRADE assessment. Although this review used a comprehensive approach, it was difficult to exclude clinically important effects due to the small number of studies on some topics and subheadings. Finally, the certainty of the evidence was downgraded due to the imprecision, indirectness, and variation of the interventions and population studies used.

The aims of this review are to promote and support appropriate complementary feeding strategies for infants. Early introduction to potential allergen foods or probiotics and increased diversity in complementary foods may protect against development of allergic disease. The findings of this work should not be applied to all infants as new recommendations for complementary feeding. A careful assessment of the safety and acceptability of complementary feeding recommendations for diverse populations is necessary (e.g., low birth weight or preterm infants, malnourished infants, and infants with HIV infection), as well as consideration of imprecise effect estimates and issues regarding indirectness.

In conclusion, this systematic review, it was found that early introduction of allergenic foods, as well as early and regular consumption of fish, probiotic supplementation, increased diversity of complementary foods, and specific complementary food patterns may reduce the risk of allergic diseases (food allergy, asthma, and AD) in the future. These findings must be considered in the context of the limitations of preliminary studies.

### Author contributions

The authors’ responsibilities were as follows – SW, WC, QZ: designed research; SW, PY, LY, FT: conducted research; SW, PY, QZ: analyzed data; SW, QZ: wrote the paper; QZ: had primary responsibility for the final content; and all authors: read and approved the final manuscript. All data relevant to the study are included in the article or as supplementary information.

### Conflict of interest

The authors declare that there are no conflicts of interest.

### Funding

This work was supported by the National Natural Science Foundation of China [No. 32122067], the Natural Science Foundation of Jiangsu Province [BK20200084]; the National Natural Science Foundation of China [No. 32021005] and supported by the Fundamental Research Funds for the Central Universities JUSRP622013.
