# Supplementary material for: Effects of Early Diet on the Prevalence of Allergic Disease in Children: A Systematic Review and Meta-Analysis
Source: Adv Nutr. 2023 Oct 10;15(1):100128. doi: 10.1016/j.advnut.2023.10.001 (PMC10831899; doi:10.1016/j.advnut.2023.10.001)
Supplement: Multimedia component 1 [file mmc1.docx]

**Effects of early diet on the prevalence of** **allergic disease in children: A systematic review and meta-analysis**

Shumin Wang

**Supplementary**

**Supplemental Methods**

**Supplemental Table 1.** Characteristics of studies early diet and future risk of allergic disease (10 intervention studies)

**Supplemental Table 2.** Characteristics of studies early diet and future risk of allergic disease (44 observational studies)

**Supplemental Table 3.** GRADE Assessment

**Supplemental Figure 1.** Funnel plot for publication bias: Food allergy

**Supplemental Figure 2.** Funnel plot for publication bias: Asthma

**Supplemental Figure 3.** Funnel plot for publication bias: Atopic dermatitis

**Supplemental Figure 4.** Risk of bias graph: Intervention trials

**Supplemental Figure 5.** Risk of bias summary: Intervention trials

**Supplemental Figure 6.** Risk of bias graph: Observational studies

**Supplemental Figure 7.** Risk of bias summary: Observational studies

**Supplemental Figure 8. Effect of early *vs.* late intake of complementary foods on risk of asthma.** Effects on all participants (A), specific period of intake (B), and period of outcome assessment (C).

**Supplemental Figure 9. Effect of early *vs.* late intake of allergenic food on risk of asthma.** Effects on all participants (A), effect of specific allergenic food (B), effect of specific timing of introduction (C), and effect of time for outcome assessment (D).

**Supplemental Figure 10. Effect of probiotic supplementation during the complementary food period on risk of asthma.** Effects on all participants (A) and effect of time for outcome assessment (B).

**Supplemental Figure 11. Effect of** **fish consumption on risk of asthma.** Effects of early fish intake frequency (A), specific frequency of fish consumption (B), period of early fish intake (C), specific timing of fish introduction (D), and time for outcome assessment (E).

**Supplemental Figure 12. Effect of high-dose vitamin D supplementation on risk of asthma.**

**Supplemental Figure 13. Effect of early *vs.* late intake of complementary foods on risk of atopic dermatitis.** Effects on all participants (A). Effect of specific timing of intake (B) and time for outcome assessment (C). Effects on infants at high/normal risk of allergy (D).

**Supplemental Figure 14. Effect of early *vs.* late intake of allergenic food on risk of atopic dermatitis.** Effects on all participants (A). Effect of specific allergenic food (B), specific timing of introduction (C), and time for outcome assessment (D).

**Supplemental Figure 15. Effect of probiotic supplementation during the complementary food period on risk of atopic dermatitis.** Effects on all participants (A) and time for outcome assessment (B).

**Supplemental Figure 16. Effect of** **fish consumption on risk of atopic dermatitis.** Effects of early fish intake (A), early fish intake time (B), specific timing of fish consumption (C), time for outcome assessment of early fish consumption (D), and time for outcome assessment of early fish introduction (E).

**Supplemental Figure 17. Effect of high-dose vitamin D supplementation on risk of atopic dermatitis.**

**Supplemental References**

**Supplemental Appendix 1. Search Strategies**

1. **Methods**

**2.1 Search strategy**

This systematic review is performed according to Preferred Reporting Items for Systematic reviews and Meta-Analyses (PRISMA) guidance^17^. We searched the following electronic databases and trial registers from the inception of each database up to May 31, 2023 (articles prior to 2000 were manually reviewed), without restrictions on the language of publication: the Cochrane Library, EMBASE, Web of Science (comprising Web of Science Core Collection, Chinese Science Citation Database℠, FSTA® - the food science resource, KCI-Korean Journal Database, MEDLINE® and SciELO Citation Index), and PubMed. Intervention trials and observational studies evaluating any dietary actors (e.g., the dietary patterns, the food diversity, the timing of solid food/allergenic food/fishes introduction, and the consumption of probiotics/fishes/vitamin D) during the complementary-food period and allergic diseases (food allergy, asthma, and atopic dermatitis) at any age were included. Details for all search strategies, including search terms, are available in the appendix (pp. 42-50). We also hand-searched the reference lists of identified studies for possible additional publications, regardless of the language. Where necessary we contacted the authors of eligible or potentially eligible studies to request original data or further details. The citations identified in searches were imported into Endnote libraries for de-duplication and title screening.

**2.2 Study inclusion criteria**

We included research studies published at any time prior to the search date. Original studies eligible for inclusion were randomized controlled trials (RCT), quasi RCT (RCT where the allocation sequence was predictable), prospective cohort trials or longitudinal studies, retrospective cohort studies, nested case-control studies, other case control studies and cross-sectional surveys. We took a hierarchical approach to study design, such that where data were absent or limited from intervention trials, we included observational study data. Where a large number of intervention trials were identified, we narratively discussed results of observational studies that assessed the same intervention/exposure. We did not include non-comparative studies, or non-human studies.

**2.3 Participants/population**

Inclusion criteria: Infants between birth and the end of their 24^th^ post-partum month. If infants were characterized as high risk for atopic disease based on family history or genotype, this information was recorded so that it could be used for the planned subgroup analysis by disease risk. The eligible intervention was consumption of complementary products, allergenic food, food supplements (e.g., vitamin D, fish oil and probiotics).

Exclusion criteria: We excluded studies in which participants were defined by a disease state (e.g., infants born prematurely). We also excluded studies in which specific dietary exposures or interventions only within the first 6 months of life. We did not exclude studies on the basis of including specific ethnic groups or studies of high-risk infants.

**2.4 Study Outcomes**

We included allergic diseases included food allergy, asthma, and atopic dermatitis. We specifically excluded rare manifestations of food allergy such as eosinophilic esophagitis because its prevalence is less than 1‰. We stratified the analysis by timepoint for outcome assessment, timepoint for dietary exposure/intervention onset, duration of dietary exposure/intervention, type of allergenic food, and specific dietary intake. For each outcome measure in this review, there is more than one possible method of assessment.

*Food allergy*^18^ - defined by double blind placebo-controlled food challenge, by open food challenge, by medical diagnosis or by self/parent report. We included reports of any food allergy, and specific food allergies to cow’s milk, egg or peanut. For the analysis of food allergy in relation to timing of allergenic food introduction we also discussed the timing of introduction of cereals, meat, fruits and vegetables. We did not include reports of food intolerance that we judged were unlikely to meet current definitions of food allergy.

*Asthma*/*wheeze*^19^ - defined as either asthma, infantile wheeze or similar, using parent/self-report, doctor diagnosis, a validated questionnaire, scoring system or objective measure such as bronchial hyper-reactivity, forced vital capacity, peak expiratory flow rate or reversible airways obstruction using forced expiratory volume in 1 second. We included data for atopic asthma/wheeze which associated with allergic sensitization (unless the study did not disclose the type of asthma). We did not include different wheezing entities based on the timing of onset/resolution of the disease such as early transient wheeze or persistent wheeze due to heterogeneity in definition between studies. We did not include outcomes such as bronchitis or bronchiolitis.

*Atopic* *dermatitis/Eczema*^19^ – defined using parent/self-report, doctor diagnosis, a validated questionnaire, scoring system or objective measure. We included data for atopic dermatitis/eczema which associated with allergic sensitization. We did not include reports of rashes which were likely to have included other cutaneous problems, such as nappy rash, contact dermatitis, rash, skin problem.

- 1. **Study selection and data extraction**

Title and abstract screening was undertaken in duplicate by a team of 4 review authors (Shumin Wang, Pingping Yin, Leilei Yu, and Fengwei Tian). Full texts of all potentially relevant records were screened for eligibility. Any disagreements were resolved through consensus or by recourse to a third author.

Data were extensively cleaned and coded for analysis with further data checks to identify publications related to the same parent study, and to identify the most appropriate output for inclusion in meta-analysis from studies reporting multiple assessments of closely related exposures/outcomes. Data cleaning was undertaken by Shumin Wang, Pingping Yin and Qixiao Zhai.

All data were extracted from included studies. If more than one exposure group was compared with the control group (exposed group with dietary exposure earlier than or at a dose greater than or longer than the control group) in relation to the same outcome at the same age, we performed separate meta-analyses. For example, a study reporting the relationship between timing of introduction of allergenic food and wheeze at age 2 years, with data for ≤4, 5-7 and >7 months duration, we would include the comparison ≤4 *vs.* >4, ≤4 *vs* >7 and ≤7 *vs.* >7 months. We used the following exposure cut-offs for timing of introduction of solid/allergenic/specific food, which were selected based on the distribution of the data presented in published reports so as to maximize our ability to undertake meta-analysis: ≤ 3 *vs.* > 3; ≤ 4 *vs.* > 4; ≤ 6 *vs.* > 6; 6-8 *vs.* > 8; ≤ 9 *vs.* > 9; 8-12 *vs.* > 12 months duration. For outcomes we grouped studies reporting outcome at ages 1, 3, 0-3, 3-6, and 6-12 (or 6-10 and 10+) years. We selected data that reported the number of participants who had an incident or the odds ratio of occurrence for analysis. Where appropriate we also considered the outcomes reported at other conditions which were not included in meta-analysis, in our interpretation of the data. Where different methods of outcome assessment were used within a study, we prioritized validated outcomes – for instance, we prioritized clinical diagnosis of asthma over asthma-associated parent/self-report. Data that could not be included in any meta-analysis, for example medians, or means without a standard deviation or standard error were reported narratively. The outcomes of both meta-analyzed and narratively reported studies were considered together when interpreting data and making conclusions.

- 1. **Risk of bias (quality) assessment**

Publication bias was assessed using funnel plots and Egger's test, for those meta-analyses with ≥10 studies included^20^. The risk of bias in included RCT studies was assessed using the Cochrane Collaboration Risk of Bias tool (RoB1)^21^, and the risk of bias in included Non-RCT trials was assessed using the Risk Of Bias In Non-randomized Studies - of Interventions tool (ROBINS-I)^22^. For all study reports, we created a summary Table of study characteristics with key study features, and a separate summary risk of bias Table showing the risk of bias for all included studies whether included in meta-analyses or reported in the narrative table.

- 1. **Strategy for data synthesis**

Meta-analysis was undertaken where ≥2 studies reported the same outcome for a given exposure. Where meta-analysis was deemed inappropriate due to differences in population, exposure/intervention or outcome; or where meta-analysis was not possible due to the nature of the data reported -individual study results were showed narratively at the end of each report. Separate analyses were undertaken for each disease outcome, for each group of similar outcome assessment methods for any given disease, and for each intervention/exposure group. In general, our approach to meta-analysis was inclusive, with data pooled for maximum statistical power, but explored for important sources of statistical or clinical heterogeneity.

- 1. **Data extraction**

Data were extracted either using raw frequencies, crude estimates of effect (including Odds ratios (OR), risk ratios (RR), incidence rate ratios, hazard ratios) or as adjusted estimates of effect. Adjusted estimates of effect were used in preference for primary analyses of observational study data. Random effect meta-analyses were performed to allow for heterogeneity between studies.

- 1. **Heterogeneity**

Heterogeneity was quantified using Higgins inconsistency test (I^2^) ^23^. We explored reasons for heterogeneity using subgroup analyses based on study level factors. We classified heterogeneity as low (I^2^<25%), moderate (I^2^ 25-50%), high (I^2^ 50-75%) or extreme (I^2^>75%).

- 1. **Data analysis**

Pooled results for binary outcomes are presented as OR using the Mantel-Haenszel method (with continuity correction of 0.5 in studies with zero cell frequencies) for pooled OR. Pooled results for continuous outcomes measured using similar scales are presented as mean differences with 95% confidence intervals (CIs). We combined OR and RR in meta-analysis and plotted as pooled OR-as the majority of cohorts, case control studies and cross-sectional studies reported this effect measure-using the generic inverse variance method^19^. Forest plots were used to visually assess pooled estimates and corresponding 95% CIs. A fixed-effects model was used to calculate the pooled effect size.

We planned to do subgroup analyses for the following characteristics: e.g., the dietary patterns, the food diversity, the timing of intervention (complementary food/allergenic food/probiotics/fishes/vitamin D) introduction, different types of allergenic food, duration of intervention, the consumption of food supplement intervention (probiotics/fishes/vitamin D) and age at outcome assess.

We did statistical analyses using RevMan 5 (version 5.4.1). We followed the GRADE approach to rate the certainty of evidence^24^. The methodology and the results are reported according to PRISMA reporting guideline. This study is registered with PROSPERO, CRD42022379264.

**Supplemental Table 1. Characteristics of studies early diet and future risk of allergic disease (10 intervention studies)**

| **Study** | **Design** | **No. in**  **Intervention**  **Group** | **No. in**  **Control**  **Group** | **Intervention** | **Population** | **Country** | **^a^Disease**  **risk** | **^b^Age**  **(yrs)** | **Outcomes**  **reported** |
| --- | --- | --- | --- | --- | --- | --- | --- | --- | --- |
| Skjerven, 2022^25^;  Skjerven, 2020^26^ | CRT | 642 | 597 | Early complementary feeding of food allergens (peanut, cow's milk, wheat, and egg) from age 3 months. | **PreventADALL trial:** Healthy newborn babies with a minimum gestational age of 35 weeks. | Norway | Normal | 1, 3 | FA (SPT-Any; SPT-Egg; Peanut; CM; Wheat);  AD (UK Working Party criteria^27^ and Hanifin and Rajka diagnostic criteria^28^) |
| Schmidt, 2019^29^ | RCT | 144 | 146 | Receive a daily mixture of LGG and BB-12 for 6 months (mean age at intervention start is 10.1 months, starting prior to attending day care) | **ProbiComp Study:** infants with birthweight >2500 g, gestational age >36 weeks, being single‐born, and and ex‐pected to start in day care at age 8‐14 months. | Denmark | Normal | 1.3 (mean age) | AD (UK Working Party criteria, Schultz-Larsen, DARC, Hanifin and Rajka diagnostic criteria and DD);  Asthma (DD) |
| Rosendahl,  2019^30^ | RCT | 486 | 489 | Receive daily VD supplementation of 10 μg (400 IU, control group) or 30 μg (1200 IU, intervention group) from 2 weeks to 24 months of age. | Mothers were of northern European ethnicity without regular medication and with a singleton pregnancy. Infants included in the study were born at term (37 to 42 weeks of gestation) with a birth weight appropriate for gestational age (birth weight SDS between −2.0 and +2.0). | Finland | Normal | 1 | FA (SPT-CM; Egg white; Wheat; Cod; Peanut; Soy, DD);  AD (DD);  Asthma (DD) |
| Wickens,  2018^31^;  Wickens,  2013^32^;  Wickens,  2012^33^;  Wickens,  2008^34^ | RCT | *L. rhamnosus* HN001: 170  *B. animalis* subsp *lactis* HN019: 171 | 171 | Receive the strain HN001 (6 × 10^9^ CFU/d) or HN019 (9 × 10^9^ CFU/d) treatment from birth to 2 years. | The infant's mother or father had a history of treated asthma, eczema, or hay fever. | New Zealand | High | 2, 4, 6, 11 | Eczema (UK Working Party criteria; SCORAD);  FA (SPT-CM; Egg white, Peanut);  Asthma (ISAAC) |
| **Study** | **Design** | **No. in**  **Intervention**  **Group** | **No. in**  **Control**  **Group** | **Intervention** | **Population** | **Country** | **^a^Disease**  **risk** | **^b^Age**  **(yrs)** | **Outcomes**  **reported** |
| Tan, 2017^8^ | RCT | 165 | 154 | Egg introduction from 4 months to 8 months of age. | **BEAT trial:** Infants with at least 1 first=degree relative with a history of any atopic disease (FA, asthma, atopic eczema, or allergic rhinitis). | Australia | High | 0.6, 1 | FA (SPT=Egg white; OFC-Egg; sIgE-Egg);  Eczema (SCORAD) |
| Palmer, 2017^14^;  Palmer, 2013^35^ | RCT | 407;  49 | 413;  37 | Egg introduction from 4 months to 10 months of age;  Egg introduction from 4 months to 8 months of age. | Singleton infants with atopic mothers (history of a medically diagnosed allergic disease with sensitization to at least 1 common aeroallergen) were recruited before age 6.5 months;  Singleton term infants with symptoms of moderate-to-severe eczema (determined by using a standardized SCORAD20 score of ≥15) were recruited at 4 months. | Australia | High | 1 | FA (SPT-Egg; OFC-Egg; sIgE-Egg);  Eczema (medical diagnosis) |
| Bellach, 2017^36^ | RCT | 184 | 199 | Egg introduction from 4 months to 12 months of age. | **HEAP study:** Infants with a gestational age of 34 weeks or greater and a birth weight of 2.5 kg or greater in 8 maternity wards in Berlin. | Germany | Normal | 1 | FA (sIgE-Egg; DBPCFC) |
| Perkin,  2016^9^ | RCT | 652 | 651 | Sequential introduction of six allergenic foods=cow’s milk, peanut, egg, wheat, sesame and fish from age 3 months, versus avoidance to ≥6 months. | **EAT study:** Children exclusively breastfed at 3 months and gestation over 37 weeks. | UK | Normal | 1, 3 | FA (OFC) |
| West, 2013^37^;  West, 2009^38^ | RCT | 89 | 90 | Receive the cereals with *L. paracasei* LF19 (1 × 10^8^ CFU/d) from 4 to13 months of age. | Healthy, term infants with no priorallergic manifestations**.** | Sweden | Normal | 1.1, 8–9 | Eczema (DD, SCORAD);  FA (sIgE);  Asthma (DD) |
| **Study** | **Design** | **No. in**  **Intervention**  **Group** | **No. in**  **Control**  **Group** | **Intervention** | **Population** | **Country** | **^a^Disease**  **risk** | **^b^Age**  **(yrs)** | **Outcomes**  **reported** |
| Abrahamsson, 2013^39^;  Abrahamsson, 2007^40^ | RCT | 95 | 93 | Receive *Limosilactobacillus reuteri* ATCC 55730 (1 × 10^8^ CFU/d) through the first year of life. | Between January 2001 and April 2003, 232 families with allergic disease (i.e., one or more family members with eczema, asthma, gastrointestinal allergy, allergic urticaria or allergic rhinoconjunctivitis) were recruited at antenatal clinics. | Sweden | High | 2, 7 | Eczema (SCORAD; Hanifin and Rajka diagnostic criteria);  FA (SPT-Egg white, CM; Peanut; sIgE-Egg white, CM; Cod, Wheat; Peanut; Soybean);  Asthma (DD and use of medications) |

CRT: cluster randomized trial; RCT: randomized controlled trial; FA: food allergy; SPT: skin prick test; SPT-Any: SPT to any food; CM: cow’s milk; AD: atopic dermatitis; LGG: *Lacticaseibacillus rhamnosus* GG; BB-12: *Bifidobacterium animalis* subsp *lactis* BB-12; DARC: Danish Allergy Research Centre; DD: doctor diagnosis; CFU: colony-forming units; SCORAD: SCORing atopic dermatitis; ISAAC: International study of asthma and allergy in children; OFC: oral food challenge; DBPCFC: double-blind placebo-controlled food challenge. ^a^Disease risk is high where the population studied were at high inherited risk of allergic disease. ^b^Age at outcome-for some studies outcome data used.

in the systematic review were derived from evaluations at multiple ages.

**Supplemental Table. 2 Characteristics of studies early diet and future risk of allergic disease (41 observational studies)**

| **Study** | **Design** | **N** | **Country** | **Population** | **Exposure(s)** | **Measure** | **^a^Age**  **(yrs)** | **Outcomes**  **reported** |
| --- | --- | --- | --- | --- | --- | --- | --- | --- |
| Chęsy, 2023^41^ | PC | 86 | Poland | All participants were patients of the Department of Paediatrics, Allergology and Gastrenterology, Collegium Medicum NCU, Allergy Clinic, and Communal Nursery in Łochowo between September 2016 and December 2019. | Timing of introduction of allergenic food: Egg; white; milk; peanut; wheat; soybean; fish; tree nuts; and shellfish. | Questionnaire | < 3 | FA (medical diagnosis and sIgE) |
| Hua, 2023^42^ | PC | 501 | China  (Taiwan) | Children who were delivered at Chang Gung Memorial Hospital, Keelung, Taiwan, between March 2012 and April 2017. | Timing of introduction of complementary food: Egg white; egg yolk; fruit; fish; shellfish; peanut. | Questionnaire and interview | 2 | AD (DD) |
| **Study** | **Design** | **N** | **Country** | **Population** | **Exposure(s)** | **Measure** | **^a^Age**  **(yrs)** | **Outcomes**  **reported** |
| Lu, 2023^43^ | RC | 2598 | China | Preschoolers (3-6 years old) in 36 kindergartens located in 6 administrative districts of Changsha, Hunan Province. | Timing of introduction of complementary foods (< 3 months; ≤ 6 months). | Questionnaire | 3-6 | FA (DD) |
| Wen, 2023^44^;  Luccioli, 2014^45^ | PC | 1252;  1542 | USA | **IFPS II (Y6FU)**: A panel survey, representative of pregnant women in USA in 2005, and who were present in the year 6 of follow-up. | Timing of introduction of allergenic food: egg (< 10 months; < 12 months).  Timing of introduction of complementary foods (4 months, 6 months). | Questionnaire | 6 | FA (parent report; DD) |
| Obaid, 2022^46^ | CC | 151 | Yemen | Children attending Childhood and Maternity Public Hospital, Ibb, Yemen Republic. | Solid food (before age 6 months). | Questionnaire | < 15 | Asthma (parent report) |
| Adjibade,  2022^47^ | PC | 8389 | France | **ELFE Cohort:** A multidisciplinary nationwide birth cohort including children born in 2011 in 320 participating maternity units among a random sample of 349 maternity units in mainland France. Born after 33 weeks of gestation, from mothers aged 18 years or older. | Probiotics:  *B. breve* BC50; *B. lactis* BB12; *L. reuteri* DSM 17938; *L. fermentum* CECT5716; *Streptococcus thermophilus* (consumed from the age of 2–10 months). | Questionnaire and interview | < 5.5 | Asthma (medical diagnosis);  Itchy rash (parent report);  FA (physician assessment) |
| Yakaboski, 2021^48^ | PC | 770 | USA | A multicenter cohort of infants hospitalized with bronchiolitis between 2011–2014. | Food allergens of egg and peanut (introduce before the age of 12 months). | Interview and clinical data | 3 | FA (sIgE and ISAC) |
| Ekelund,  2021^49^;  Øien, 2010^50^ | PC | 6802;  3086 | Norway | **Substudy of PACT study:** All children provided data on breastfeeding duration or complementary food introduction and allergy-related disease at two or six years. | Solid food (before age 6 months);  Fish consumption in the first year of life. | Questionnaire | 2, 6 | Asthma (DD);  Eczema (parent report) |
| Hose,  2021^51^ | PC | 1361 | Germany | **PASTURE (N=1133):** 1133 children in 2002–2005 from rural areas in 5 European countries: Austria, Finland, France, Germany, and Switzerland; **LUKAS2 (N=228):** All pregnant women and their infants with scheduled delivery at Kuopio University Hospital between May 2004 and May 2005. | Feeding patterns: Unbalanced meat consumption was characterized daily meat consumption and rare consumption of milk and yoghurt (in the first year of life). | Questionnaire and diary | 6 | Asthma (DD) |
| **Study** | **Design** | **N** | **Country** | **Population** | **Exposure(s)** | **Measure** | **^a^Age**  **(yrs)** | **Outcomes**  **reported** |
| Venter,  2020^52^ | PC | 969 | UK | **FAIR birth cohort:** children born on the UK between 2001–2002 who were followed up prospectively, providing information on sociodemographic, environmental, and dietary exposures. | Dietary diversity (in the first year of life): FD; FVD; defined by WHO and FAD. | Questionnaire | 1, 2, 3, 10 | FA (SPT-milk; wheat; egg; cod; peanut and sesame, OFC and DBPCFC);  Eczema (ISAAC) |
| Thorsdottir, 2019^53^ | PC | 144 | Iceland | Icelandic (Caucasian) parents, singleton birth, 37–41 weeks gestational length, birth weight within 10th–90th percentiles, absence of birth defects or congenital long–term diseases and regular antenatal care of the mother. | VD (in the first year of life);  Solid food (before age 4 months). | Records, diary and questionnaire | 1, 6 | FA (SPT-milk, egg, cod, wheat, soy and peanut) |
| Klingberg, 2019^54^ | PC | 9727 | Sweden | **ABIS cohort:** Babies born in the counties of Östergötland, Småland, Blekinge, and Öland from 1 October 1997 to 1 October 1999. | Timing of introduction of complementary food: Milk; egg; fish; meat; cereals with gluten; cereals without gluten; Fruits and berries; vegetables and potatoes et al. | Diary and questionnaire | 15–17 | Atopic asthma (medical diagnosis) |
| Tham, 2018^13^;  Loo, 2017^55^ | PC | 1152 | Singapore | **GUSTO cohort:** Singleton, naturally  conceived pregnancies. | Timing of introduction of allergenic food: CM; egg yolk, egg white; peanut; and shellfish;  Dietary patterns from 6–12 months: Predominantly breastmilk; According to Guidelines-rice porridge, vegetables, fruits and low–fat fish and meat; Easy-to-prepare foods-infant cereals, juices, cake and biscuits; and Noodles (in soup) and seafood-rice and wheat noodles and common accompaniments such as fish and shellfish. | Questionnaire | 3;  1.5, 3, 5 | FA (SPT-milk; egg; peanut and shellfish);  FA (SPT-egg, peanut, CM, shrimp and crab);  Eczema (ISAAC);  Wheeze (ISAAC); |
| **Study** | **Design** | **N** | **Country** | **Population** | **Exposure(s)** | **Measure** | **^a^Age**  **(yrs)** | **Outcomes**  **reported** |
| Clausen, 2018^10^ | PC | 1304 | Iceland | **EuroPrevall birth** **cohort:** A multi-center birth cohort study, recruiting a total of over 12000 newborns in nine countries across Europe in 2005–2009. Exam prevalence patterns and influential factors of confirmed food allergies in European children from different regions. | Fish oil supplementation: Timing of regular fish oil intake and dose of fish oil consumption. | Interview and questionnaire | 2.5 | FA (SPT-milk; egg; peanut; fish; wheat and soy, sIgE, DBPCFC) |
| Lossius, 2018^56^ | PC | 31930 | Norway | **MoBa cohort:** A prospective population-based pregnancy cohort study conducted by the Norwegian Institute of Public Health. | Timing of introduction of complementary foods (< 4 months; 4–6 months; > 6 months). | Questionnaire and clinical data | 3, 7 | Asthma (medical diagnosis and use of medications) |
| Nwaru, 2017^57^;  Nwaru, 2014^58^;  Nwaru, 2013^59^;  Nwaru, 2010^60^;  Virtanen, 2010^61^ | NCC;  PC;  PC | 910;  3142;  3871;  994;  1302 | Finland | **DIPP:** A case-control analysis nested within the DIPP Nutrition cohort and comprising all incident asthma cases (n=182) and four randomly selected matched controls (n=728);  **DIPP:** Prospective birth cohort of  children at high risk of TIDM (HLA  genotype conferred susceptibility) born  between 1997 and 2004 in Oulu and  Tampere University Hospital Finland. | Vitamin D from foods and supplements and both combined (3, 6, and 12 months);  Timing and diversity of introduction of complementary at 3, 4, 6, and 12 months of age. | Records and questionnaire | 5 | Asthma (DD, use of medications and ISAAC);  FA (sIgE) |
| Elbert, 2017^62^ | PC | 5202 | Netherlands | This study was embedded in the Generation R Study, a population-based prospective cohort study from fetal life onwards. | Timing of introduction of allergenic food: CM, hen's egg, peanut, tree nuts, soy and gluten. | Questionnaire | 1, 2, 3, 4, 10 | Eczema (DD) |
| Turati, 2016^63^ | CC | 902 | Italy | Outpatient children aged 3–24 months whom a first diagnosis of AD in 10 Italian hospital centers in the northern and central Italy. | Timing of introduction of complementary foods (4–5 months). | Interview and questionnaire | < 2 | AD (DD) |
| **Study** | **Design** | **N** | **Country** | **Population** | **Exposure(s)** | **Measure** | **^a^Age**  **(yrs)** | **Outcomes**  **reported** |
| Gabet, 2016^64^ | PC | 1860 | UK | **PARIS:** full-term and healthy singletons. To assess the prevalence and patterns of allergic sensitization and related factors at 18 months of age. | Timing of introduction of meat (6 months). | Questionnaire | 1.5 | FA (sIgE) |
| Peters, 2015^65^ | PC | 5276 | Australia | **HealthNuts study:** A prospective, population-based cohort of 12-month-old infants. | Timing of introduction of egg and peanut. | Questionnaire | 1 | Eczema (DD) |
| Morales-Romero, 2015^66^ | CS | 760 | Mexico | Analytical, cross–sectional population-based study conducted in 6–12-year-old children attending primary school and selected through a multistage sampling technique. | Timing of introduction of complementary foods (4 months). | Questionnaire | 6–12 | AD (ISAAC) |
| Roduit, 2014^67^ | PC | 856 | Austria, Finland, France, Germany, and Switzerland | **PASTURE/EFRAIM:** A prospective birth cohort involving children from rural areas in 5 European countries designed to evaluate risk factors and preventive factors for atopic diseases. From families living in  a farm and from families not living on a  farm of the same area. | Food diversity introduced in the first year of life. | Diary and questionnaire | 1, 1.5, 2, 3, 4, 5, 6 | FA (DD and sIgE) |
| Niinivirta, 2014^68^ | PC | 256 | Finland | Mother over 18, pregnancy <17 weeks  and the child having increased risk for  allergy. | Timing of introduction of CM, egg, fish, cereal. | Diary | 4 | AD (Physician assessment) |
| Grimshaw, 2014^69^ | NCC | 123 | UK | **PIFA study:** A prospective birth cohort of 1140 babies recruited between 2006 and 2008 and comprised the UK cohort of the EuroPrevall project. This NCC study: 41 infants given a diagnosis of FA based on results of DBPCFC in the first 2 years of life and their 82 age-matched control subjects. | Dietary patterns in the first year of life. | Diary and questionnaire | 2 | FA (DBPCFC or a convincing history of anaphylaxis) |
| **Study** | **Design** | **N** | **Country** | **Population** | **Exposure(s)** | **Measure** | **^a^Age**  **(yrs)** | **Outcomes**  **reported** |
| Magnusson, 2013^11^ | PC | 3285 | Sweden | **BAMSE:** A population-based, prospective birth cohort. A total of 4089 newborns were recruited between February 1994 and November 1996 in a predefined area of Stockholm, Sweden. | Fish consumption at age 1 year. | Questionnaire | 8, 12 | Asthma (Parent report and use of medications);  Eczema (Parent report and use of medications) |
| Nwaru, 2013b^70^ | PC | 1924 | Scotland | **SEATON birth cohort:** Infant feeding practices (breastfeeding and introduction of complementary foods) of singleton children in the Study of Eczema and Asthma To Observe the influence of Nutrition birth cohort. | Timing of introduction of complementary foods (fruit juice, CM/milk products, rice/cereal, vegetables, fruits, biscuits/bread, meat, fish and eggs). | Questionnaire | 1, 2, 5, 10 | Asthma (ISAAC);  Eczema (ISAAC) |
| Goksör, 2013^71^;  Alm, 2011^72^;  Alm, 2009^73^ | PC | 5654;  4496;  4921 | Sweden | A prospective, longitudinal cohort study of children born in the region of western Sweden in 2003. | Introduction of fish before 9 months of age or Fish once a month or more at 1 year of age. | Questionnaire | 12;  4.5;  1 | Atopic asthma (DD);  FA (DD);  Eczema (DD) |
| Tromp, 2012^74^;  Tromp, 2011^75^ | PC | 2173;  6905 | Netherlands | **GENERATION R:** A population–based prospective cohort study. Population based  birth cohort with pregnant women recruited < 25 weeks gestation in Rotterdam. | Dietary patterns at 14 months of age;  Timing of introduction of the allergenic foods CM, hen's egg, peanuts, tree nuts, soy, and gluten before the age of 6 months. | Questionnaire | 2, 3, 4 | Asthma (ISAAC; DD);  Eczema (ISAAC; DD) |
| GINIplus and LISAplus Study Groups, 2011^76^ | PC | GINIplus: 5991;  LISAplus: 3097 | Germany | **GINIplus:** Between September 1995 and July 1998, a total of 5991 term newborn infants were recruited from 2 regions of Germany (Munich, Bavaria, and Wesel, North-Rhine-Westfalia);  **LISAplus:** 3097 newborns were recruited between November 1997 and January 1999 from 4 German cities: Munich, Leipzig, Wesel, and Bad Honnef. | Timing of introduction of complementary foods;  Dietary diversity at 4 months and 6 months. | Questionnaire | 2 | Eczema (DD);  FA (sIgE-Egg; CM, and Peanut) |
| **Study** | **Design** | **N** | **Country** | **Population** | **Exposure(s)** | **Measure** | **^a^Age**  **(yrs)** | **Outcomes**  **reported** |
| Chuang, 2011^77^ | PC | 18733 | China (Taiwan) | **TBCS:** A total of 369 towns in Taiwan were divided into 12 strata according to the administrative division (four strata), and the total fertility rate (three strata) was ranked, and 90 towns of the total were sampled. Newborns and their mothers were sampled randomly from these 90 towns, for a total of 24,200 pairs. | Timing of introduction of complementary foods. | Interview | 1.5 | AD (Physician diagnosis) |
| Sariachvili, 2010^78^ | NCC | 1128 | Belgium | **PIPO cohort:** cases and controls with  data regarding development of eczema  and timing of introduction of solid foods  were identified from this prospective  cohort: Belgium. | Timing of introduction of CM, cereal, egg, fish. | Questionnaire | 4 | Eczema (ISAAC) |
| Hetzner, 2009^79^ | PC | 7900 | USA | **ECLS–B:** A nationally representative sample of children born in the United States during 2001 who were followed from 9 months of age through kindergarten. | Introduced solid food before 6 months. | Interview and questionnaire | 2 | Asthma (Parent report) |
| Bäck, 2009^80^ | PC | 123 | Sweden | In 1998, the families of the first 206 babies, born consecuti-vely from January to March at the Department of Obstetrics at the University Hospital in Umeå. | Daily intake of VD in the first year of life (≤ 13.0 µg *vs.* > 13.1 µg). | Questionnaire | 6 | AD (Parent report) |
| Zutavern, 2008^81^;  Zutavern, 2006^15^ | PC | 2073;  2612 | Germany | **LISA:** Population based cohort study of newborns recruited between 1997 and1999 from 4 German cities: Munich, Leipzig, Wesel, and Bad Honnef. | Timing of introduction of complementary foods. | Questionnaire | 2, 6 | FA (sIgE-Egg, Milk, Cod, Rye, Wheat, Soy, and Peanut);  Eczema (DD) |
| Snijders, 2008^82^ | PC | 2558 | Netherlands | **KOALA:** Population based birth cohort with healthy pregnant women recruited in week 10–14 of their pregnancy from an ongoing prospective cohort study on pregnancy–related pelvic girdle pain and through posters in organic food shops, anthroposophical, physician offices, and midwives. | Timing of introduction of complementary foods. | Questionnaire | 2 | Eczema (UK Working Party  criteria);  Wheeze (DD) |
| **Study** | **Design** | **N** | **Country** | **Population** | **Exposure(s)** | **Measure** | **^a^Age**  **(yrs)** | **Outcomes**  **reported** |
| Mihrshahi, 2007^83^ | PC | 516 | Australia | **CAPS:** Pregnant women from antenatal clinics of 6 hospitals of Sydney between 1997 and 1999 with unborn children at high risk of asthma. | Timing of introduction of complementary foods CM, eggs, nuts or fish. | Interview | 5 | AD (UK Working Party  criteria);  Wheeze (parent reported);  FA (SPT-Egg, CM, Peanuts) |
| Filipiak, 2007^84^ | PC | 4753 | Germany | Between September 1995 and July 1998, a total of 5991 term newborn infants were recruited from 2 regions of Germany (urban Munich, Bavaria, and rural Wesel, North-Rhine-Westfalia). | Timing of introduction of complementary foods (past 4 months or past 6 months). | Questionnaire | 2 | Eczema (DD) |
| Kull, 2006^85^ | PC | 2965 | Sweden | **BAMSE:** Prospective birth cohort of newborns in a predefined area of Stockholm, Sweden between 1994 and 1997. | Fish consumption during the first year of life. | Questionnaire | 4 | AD (Parent reported);  Asthma (parent reported);  FA (sIgE-Milk; Egg; Fish; Soy; Peanut and Wheat) |
| Sahakyan, 2006^86^ | CC | 240 | Armenia | Cases were children aged 1–7 years, living in Yerevan, who have been diagnosed with AD by pediatricians at the Allergy Department of the Republican Children’s  Hospital in Yerevan and registered at the Registry of the Allergy Department, Armenia. Additional criterion for cases to be included in our study was onset of AD after 12 months post–term. Controls were children aged 1–7 years living in Yerevan who have never been diagnosed with AD. | Timing of introduction of complementary cereal porridge, vegetables, fruits, meat, eggs, cottage cheese, bread or biscuit, and yogurt. | Interview and questionnaire | 1–7 | AD (Physician diagnosis) |
| Dunlop, 2006^87^ | PC | 1326 | Slovakia | **Slovak birth cohor**t**:** The 1st 250 pregnant women delivering at maternity hospitals in the selected study sites were recruited between 1997 and 1999. | Timing of introduction of CM,  fish, egg, nuts. | Questionnaire | 1 | Atopic eczema (Physician assessment) |
| **Study** | **Design** | **N** | **Country** | **Population** | **Exposure(s)** | **Measure** | **^a^Age**  **(yrs)** | **Outcomes**  **reported** |
| Hyppönen, 2004^88^ | PC | 6007 | Finland | **NFBC 1966:** Consists of all births in the two most northern provinces of Finland,  which continued after the 24th week of pregnancy and where the expected date of delivery was in 1966. | Received VD supplementation regularly during the first year. | Questionnaire | 31 | Asthma (Asthma with wheezing during the past 12 months or by current use of asthma medication) |
| Zutavern, 2004^89^ | PC | 642 | UK | Population based birth cohort of newly pregnant women who presented at one of three general practices in Ashford, Kent UK between 1993 and 1995. | Timing of introduction of complementary foods CM, cereal, rice, fish, egg. | Questionnaire | 5–5.5 | Eczema (DD);  Wheeze (Parent report) |
| Nafstad, 2003^90^ | PC | 2531 | Norway | **The Environment and Childhood Asthma study in Oslo:** Population based birth cohort of newborn children included born in Oslo, Norway in 1992. | Fish consumption during the first year of life. | Questionnaire | 4 | Asthma (DD) |
| Schoetzau, 2002^91^ | PC | 1121 | Germany | **GINI cohort:** Between September 1995 and July 1998, a total of 2252 healthy term newborns with a family history of atopy were recruited in 16 maternity wards in the regions of Munich, Bavaria (n = 51165) and Wesel, North–Rhine–Westfalia (n = 51087). | Timing of introduction of complementary foods. | Diary and questionnaire | 1 | AD (Physician diagnosis) |

RC: retrospective cohort study; CC: case-control study; CS: cross-sectional study; PC: prospective cohort; NCC: nested case-control study; sIgE: specific IgE; ISAC: immune-solid-phase allergen chip; DD: doctor diagnosis; OFC: oral food challenges; DBPCFC: double-blind, placebo-controlled, food challenge; ISAAC: International study of asthma and allergy in children; FD: defined in terms of diversity of foods eaten; FVD: fruit and vegetable diversity; WHO: World Health Organization; FAD: food allergen diet diversity; CM: cow’s milk; VD: vitamin D; DBPCFC: double-blind placebo-controlled food challenge; AD: atopic dermatitis. ^a^Age at outcome-for some studies outcome data used.

**Supplemental Table. 3 GRADE Assessment**

| **Early dietary exposure and outcome** | **Study design** | **Risk of bias** | **Inconsistency** | **Indirectness** | **Imprecision** | **Evidence GRADE** |
| --- | --- | --- | --- | --- | --- | --- |
| **Food allergy** |  |  |  |  |  |  |
| Early introduction of complementary foods | 6 PCs  1 RC  n = 10479 | Not serious; 1 study at high risk of bias | Serious; extreme statistical heterogeneity in one analysis; study estimates vary from 0.32 to 4.44 for the studies at low risk of bias | Not serious; 5 studies were in representative birth cohorts; 2 in a birth cohort selected for high risk of asthma; findings were consistent | Not serious; 95% CIs were wide but > 10000 participants included | Very low |
| Early introduction of allergenic food | 5 RCTs  6 PCs  1 CRT  n = 14439 | Not serious; 3 studies at high risk of bias | Not serious; I^2^ = 0 to 53% for different analyses; with estimates ranging from 0.07 to 1.88 | Not serious; 5 studies were in representative birth cohorts; 2 in a birth cohort selected for high risk of atopic disease; findings were consistent | Not serious; ORs from 0.13 to 0.72; overall > 10000 participants included | Low |
| Supplementation with probiotics | 6 RCTs  n = 2897 | Not serious; none study at high risk of bias | Not serious; I^2^ = 0 for different analyses; with estimates ranging from 0.60 to 1.15 | Not serious; all studies recruited infants whose family members with atopic disease | Not serious; ORs from 0.72 to 0.86; overall > 2000 participants included | High |
| Fish consumption | 2 PCs  n = 5578 | Not serious; 2 studies at low risk of bias | Not serious; I^2^ = 0; study estimates vary from 0.50 to 0.54 | Not serious; 2 studies were in representative birth cohorts | Not serious; allergy severity decreased with increased fish consumption, dose dependent effect | Moderate |
| Vitamin D supplementation | 2 PCs  1 RCT  n = 2423 | Not serious; all studies at low risk of bias | Not serious; I^2^ = 0; study estimates vary from 0.80 to 0.98 | Not serious; all study recruited infants without high risk of atopic disease | Not serious; overall > 2000 participants included | Low |
| **Early dietary exposure and outcome** | **Study design** | **Risk of bias** | **Inconsistency** | **Indirectness** | **Imprecision** | **Evidence GRADE** |
| Diet diversity | 4 PCs  n = 5701 | Not serious; none study at high risk of bias | Not serious | Serious; The evaluation criteria for food diversity are not uniform | Not serious; overall > 5000 participants included and with a dose-response effect | Low |
| **Asthma** |  |  |  |  |  |  |
| Early introduction of complementary foods | 7 PCs  1 CC  1 CS  n = 55302 | Serious; 2 study at serious risk of bias, 2 study at high risk of bias | Not serious; I^2^ = 0 to 60% for different analyses due to studies with serious risk of bias; with estimates ranging from 0.57 to 1.35 for the studies at low risk of bias | Not serious; 1 study in a birth cohort selected for high risk of asthma | Not serious; overall > 50000 participants included | Very low |
| Early introduction of allergenic food | 6 PCs  n = 28101 | Not serious; all studies at low risk of bias | Not serious; I^2^ = 0 to 68% for different analyses, heterogeneity is likely to be explained by differences in assessment ages of outcome; ranging from 0.35 to 1.71 | Not serious; 6 studies were in representative birth cohorts | Not serious; overall > 20000 participants included | Low |
| Supplementation with probiotics | 5 RCTs  n = 2596 | Not serious; none study at high risk of bias | Not serious; I^2^ = 0 in different analyses; with estimates ranging from 0.51 to 1.08 | Not serious; 5 RCTs recruited infants with a high risk of atopic disease | Not serious; overall > 2000 participants included | High |
| **Early dietary exposure and outcome** | **Study design** | **Risk of bias** | **Inconsistency** | **Indirectness** | **Imprecision** | **Evidence GRADE** |
| Fish consumption | 9 PCs  n = 33063 | Not serious; 1 study at high risk of bias | Not serious; I^2^ = 0 to 55% in different analyses; with estimates ranging from 0.35 to 1.11 | Not serious; 9 studies were in representative birth cohorts; asthma/wheeze (parent report) in 3 studies is an indirect measure of atopic dermatitis | Not serious; overall > 30000 participants included | Low |
| Vitamin D supplementation | 2 PCs  1 RCT  1 NCC  n = 7526 | Serious; 1 study at critical risk of bias, 1 study at high risk of bias | Not serious; I^2^ = 17% (*P* = 0.31); with estimates ranging from 0.32 to 1.34 for studies at low risk of bias | Not serious; all study recruited infants without high risk of atopic disease | Not serious; overall > 7000 participants included | Very low |
| Diet diversity | 4 PCs  n = 16957 | Not serious; 1 study at high risk of bias | Not serious | Not serious | Not serious; overall > 10000 participants included and with a dose-response effect | Moderate |
| **Atopic dermatitis** |  |  |  |  |  |  |
| Early introduction of complementary foods | 11 PCs  2 CCs  1 NCC  1 CS  1 CRT  n = 45906 | Not serious; 1 study at serious risk of bias | Serious; I^2^ = 0-70% for different analyses; study estimates vary from 0.12 to 3.10 | Not serious; 1 study recruited newborns with a family history of atopy (n = 1121) | Not serious; 95% Cls were wide for some subgroup analyses but overall > 40000  participants were included | Very low |
| Early introduction of allergenic food | 11 PCs  2 RCTs  1 CRT  1 NCC  n = 37824 | Not serious; 2 studies at high risk of bias (n = 2454) | Not serious; I^2^ = 78% in a subgroup analysis due to 1 study with high risk of bias, I^2^ = 0-46% in other analyses; study estimates vary from 0.24 to 1.92 | Not serious; 2 RCTs recruited infants with a high risk of atopic disease (n = 1139), other studies recruited infants without a high risk of atopic disease | Not serious; overall > 30000  participants were included | Low |
| **Early dietary exposure and outcome** | **Study design** | **Risk of bias** | **Inconsistency** | **Indirectness** | **Imprecision** | **Evidence GRADE** |
| Supplementation with probiotics | 7 RCTs  n = 808 | Not serious; none study at high risk of bias | Not serious; I^2^ = 0 to 56% for different analyses; with estimates ranging from 0.34 to 1.16 | Not serious; 6 RCTs recruited infants with a high risk of atopic disease, 1 RCT recruited infants with no priorallergic manifestations | Not serious; overall > 400  participants were included | High |
| Fish consumption | 11 PCs  1 NCC  n = 27399 | Not serious; 2 studies at high risk of bias (n = 2454), 1 study at serious risk of bias (n = 3086) | Not serious; I^2^ = 0 to 91% for different analyses due to studies with high risk of bias; with estimates ranging from 0.32 to 1.56 | Not serious; eczema (parent report) in 2 studies is an indirect measure of atopic dermatitis, one of the studies with serious risk of bias | Not serious; overall > 20000  participants were included | Low |
| Vitamin D supplementation | 1 RCT  1 PC  n = 1098 | Not serious; 1 study at high risk of bias (n = 123) | Not serious; I^2^ = 90%; heterogeneity is likely to be explained by differences in assessment methods of outcome | Serious; atopic dermatitis which were parent reported in PC studies is an indirect measure | Not serious; overall > 400  participants were included | Very low |
| Diet diversity | 4 PCs  n = 15604 | Not serious; 1 study at high risk of bias | Not serious | Not serious | Not serious; overall > 10000 participants included | Low |


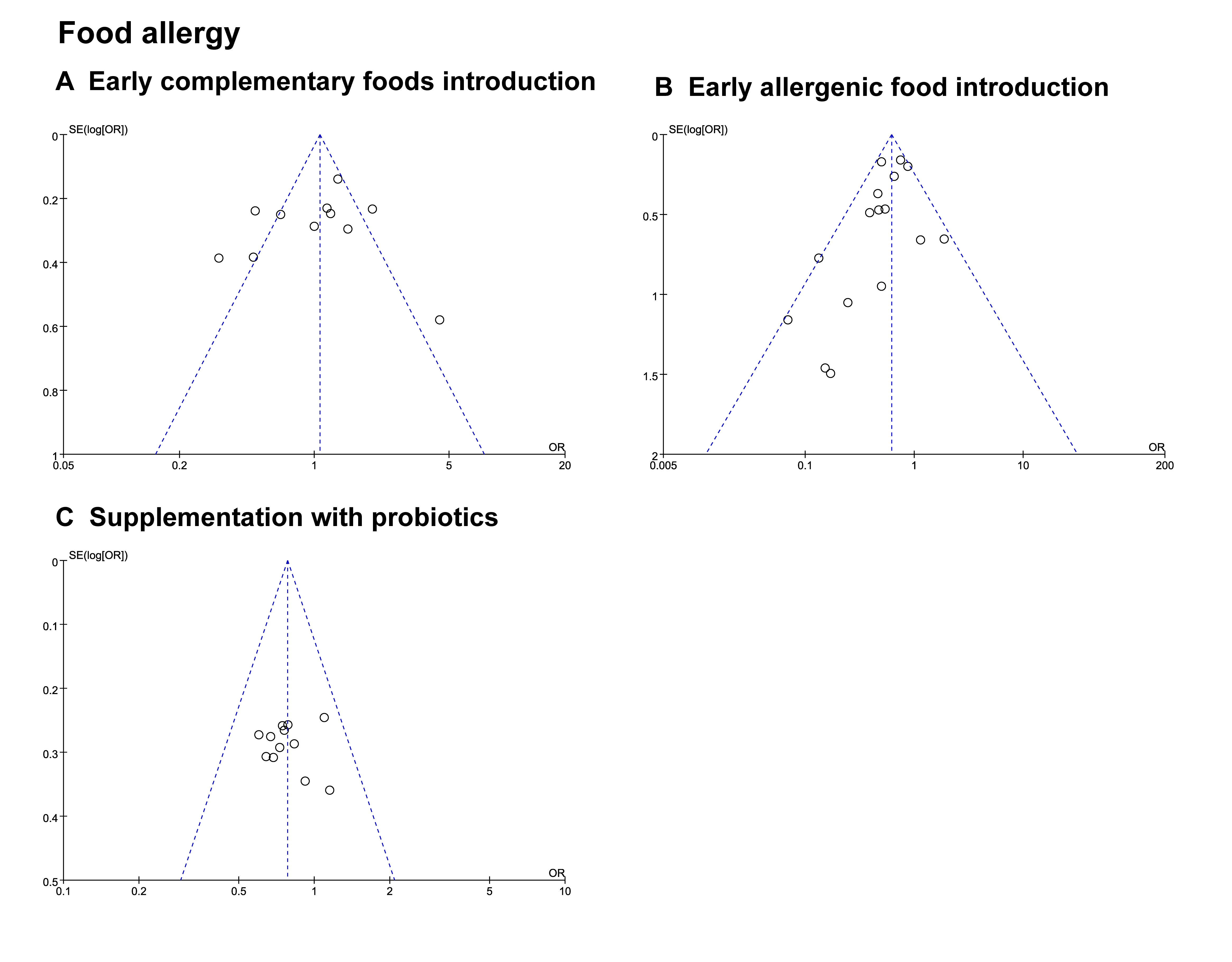


**Supplemental Figure 1.** **Funnel plot for publication bias: Food allergy**

Corresponding to Figure 2-4 in the manuscript.


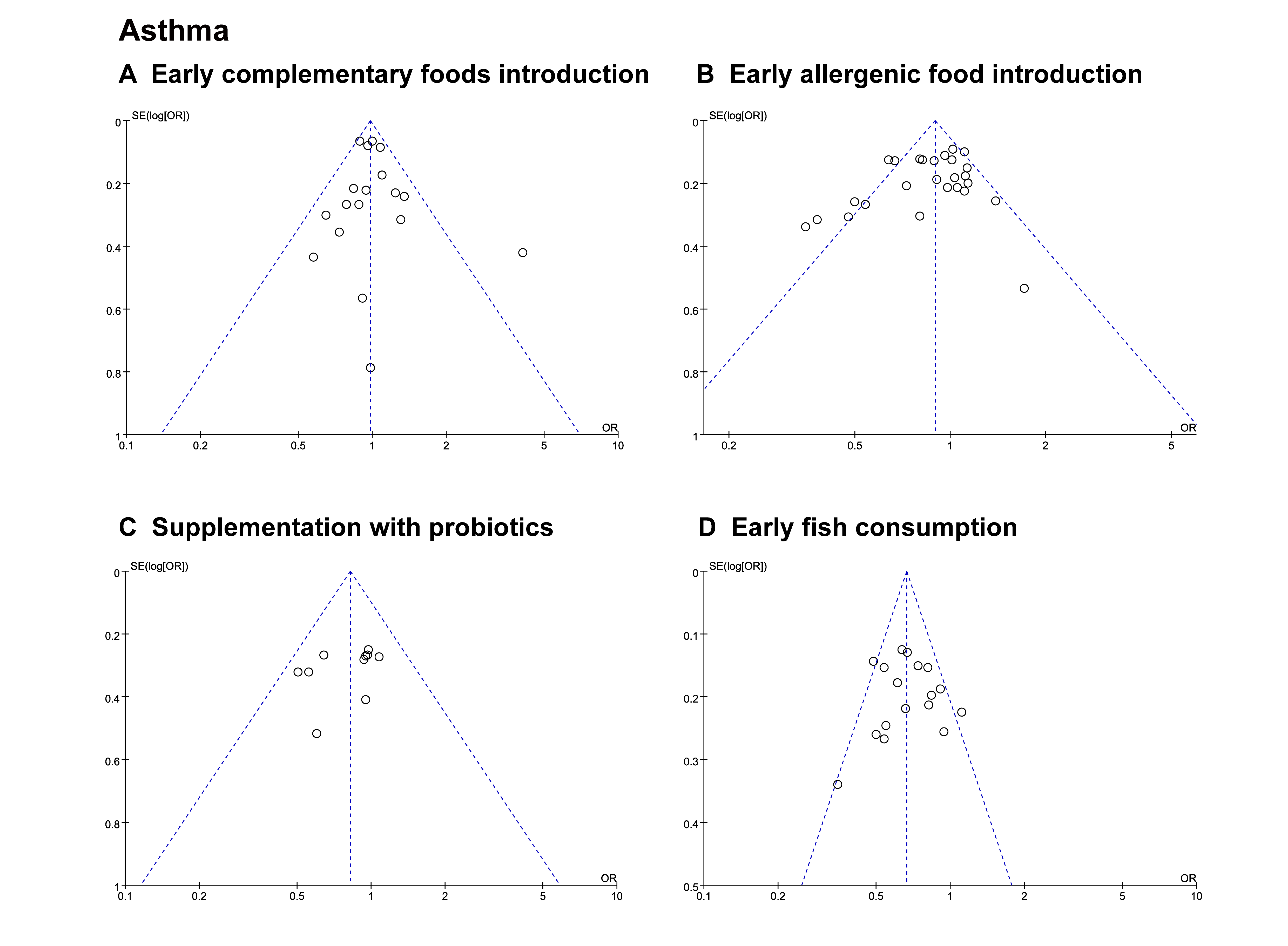


**Supplemental Figure 2.** **Funnel plot for publication bias: Asthma**

Corresponding to Supplemental Figure 8-11 in the manuscript.


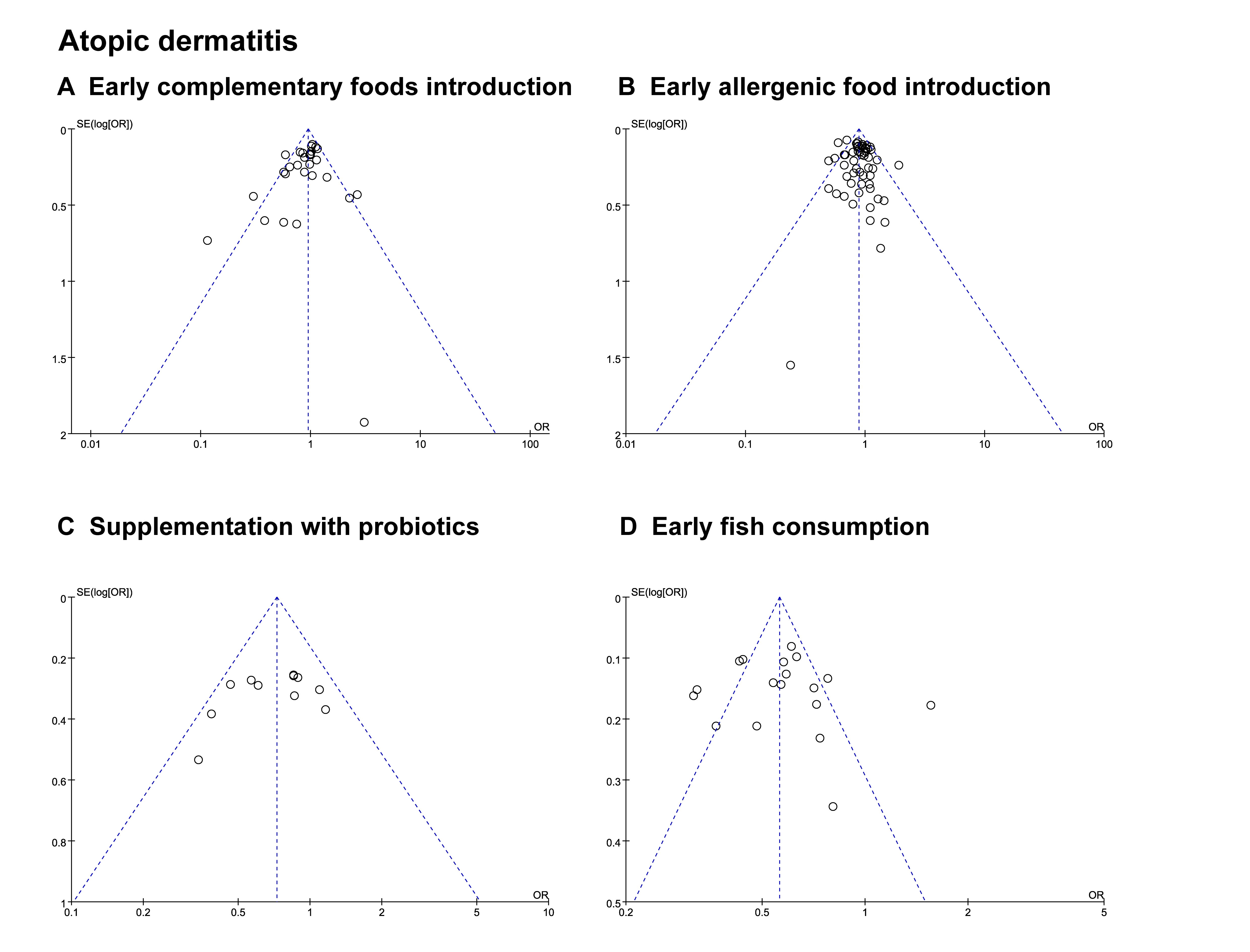


**Supplemental Figure 3.** **Funnel plot for publication bias: Atopic dermatitis**

Corresponding to Supplemental Figure 13-16 in the manuscript.


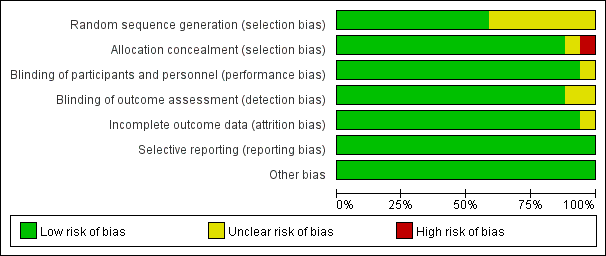


**Supplemental Figure 4. Risk of bias graph: Intervention trials**


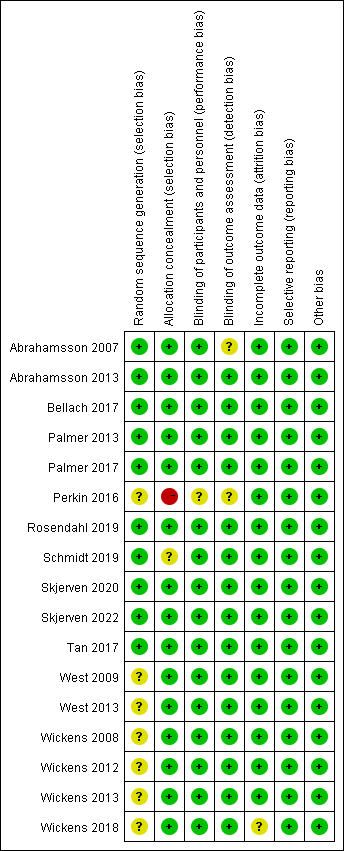


**Supplemental Figure 5. Risk of bias summary: Intervention trials**


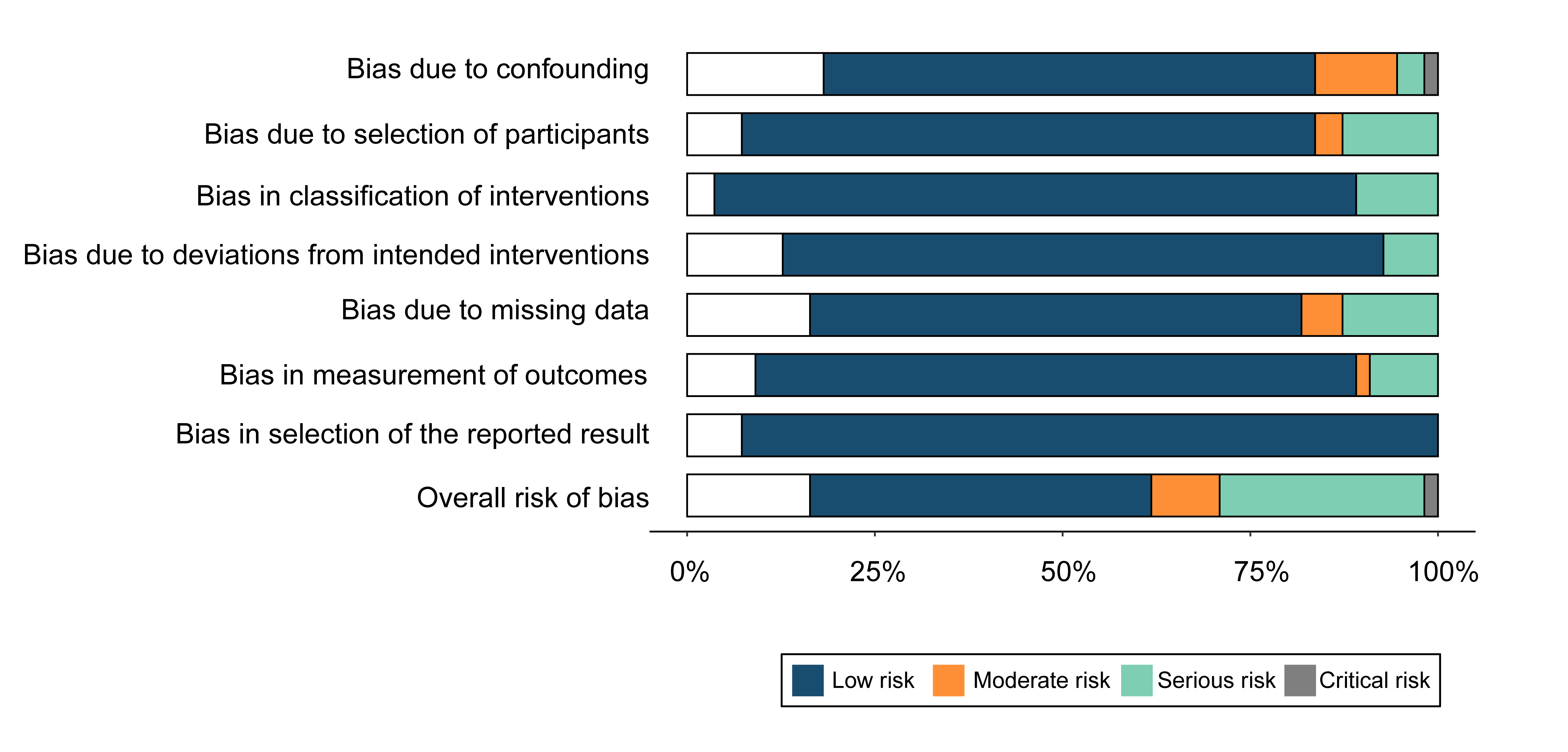


**Supplemental Figure 6. Risk of bias graph: Observational studies**


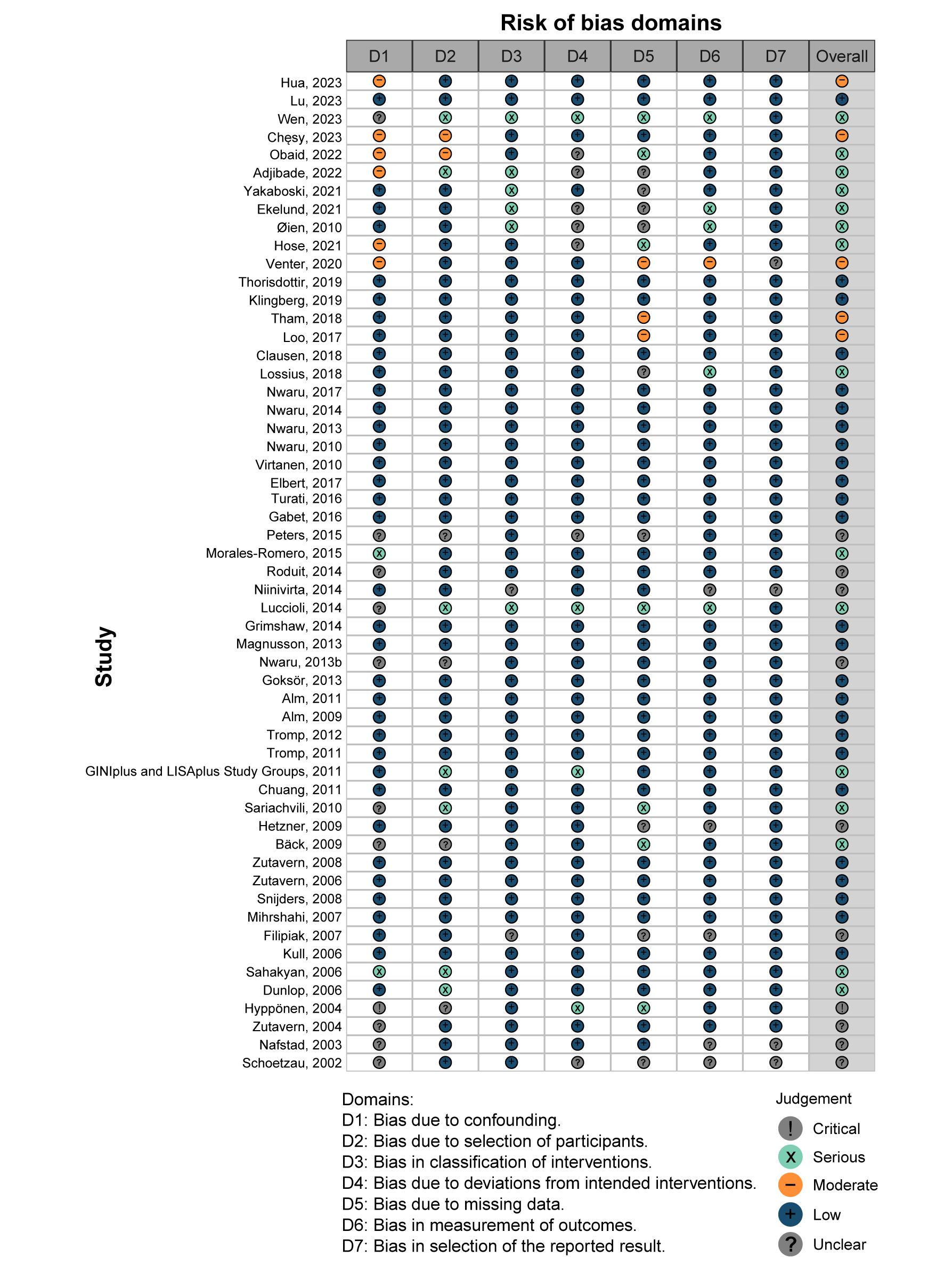


**Supplemental Figure 7.** **Risk of bias summary: Observational studies**


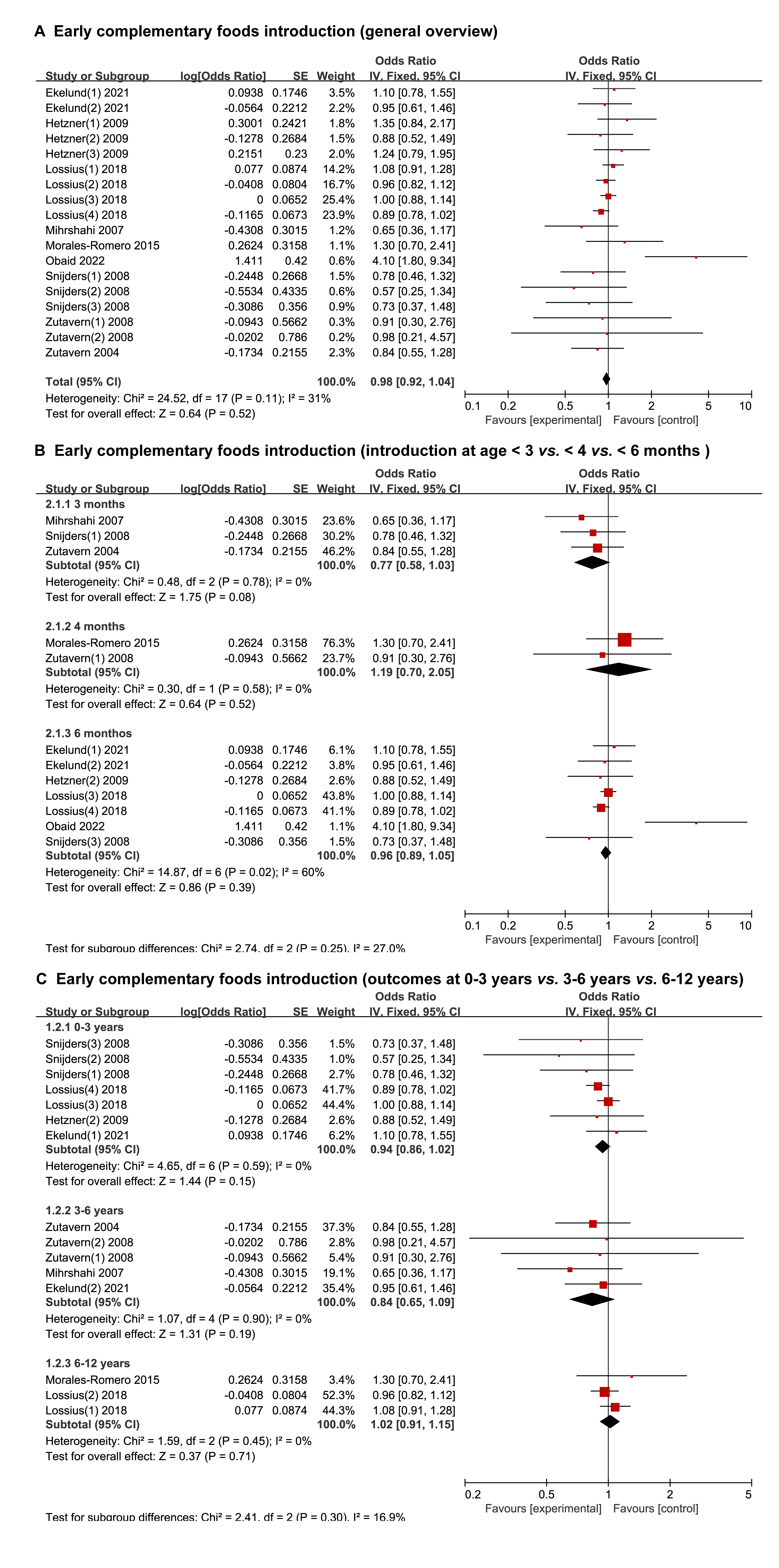


**Supplemental Figure 8. Effect of early *vs.* late intake of complementary foods on risk of asthma.** Effects on all participants (A), specific period of intake (B), and period of outcome assessment (C).


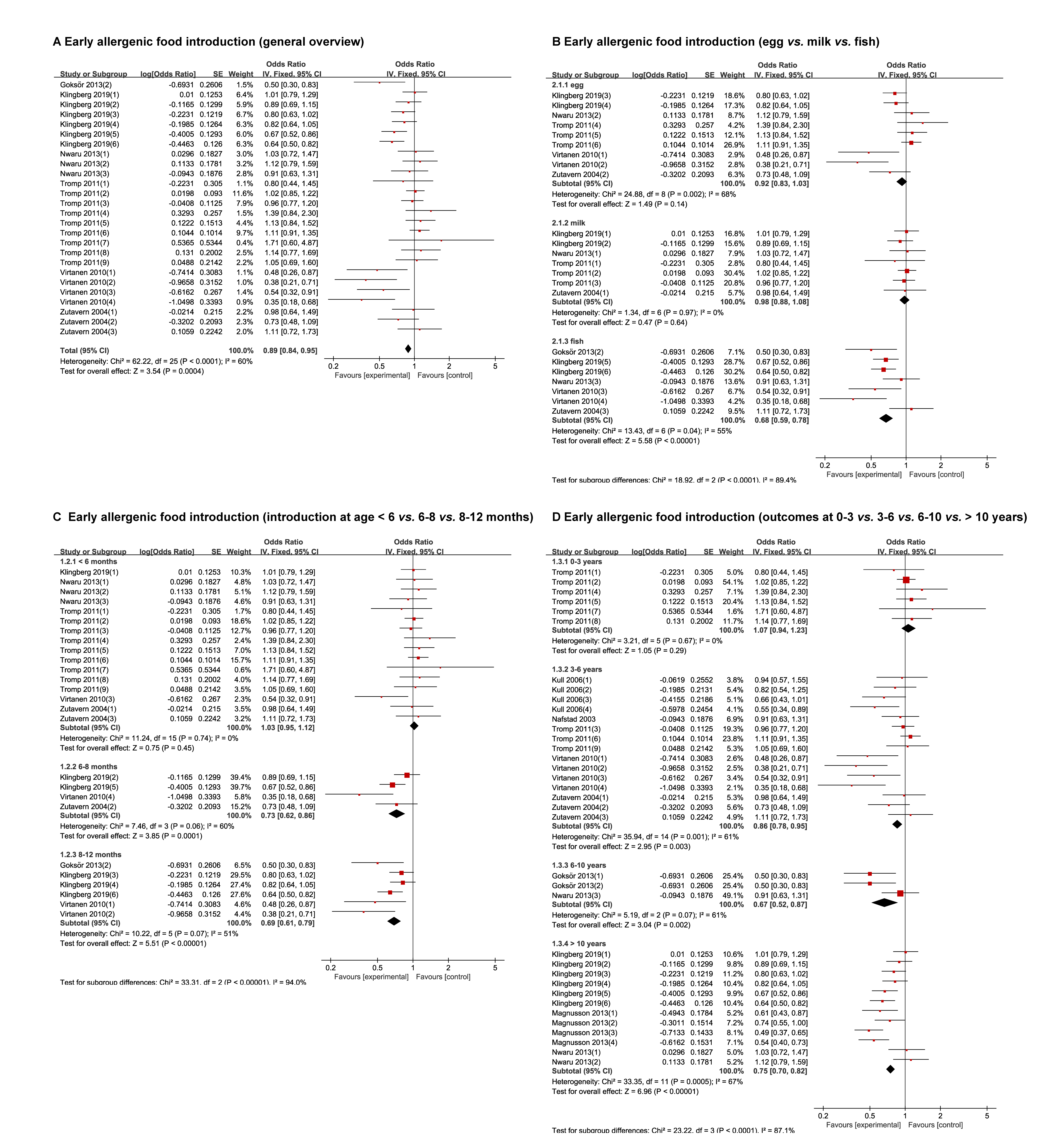


**Supplemental Figure 9. Effect of early *vs.* late intake of allergenic food on risk of asthma.** Effects on all participants (A), effect of specific allergenic food (B), effect of specific timing of introduction (C), and effect of time for outcome assessment (D).


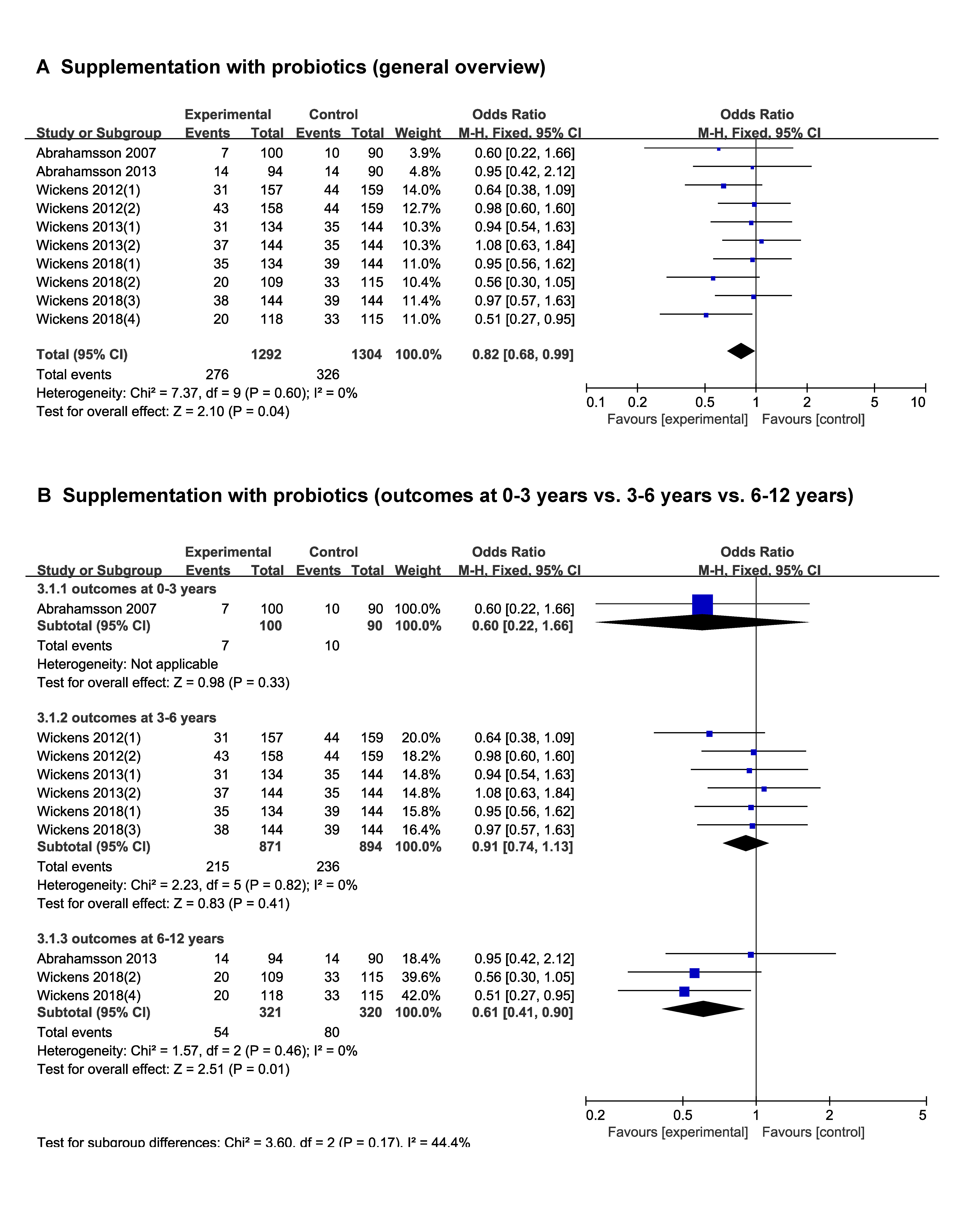


**Supplemental Figure 10. Effect of probiotic supplementation during the complementary food period on risk of asthma.** Effects on all participants (A) and effect of time for outcome assessment (B).


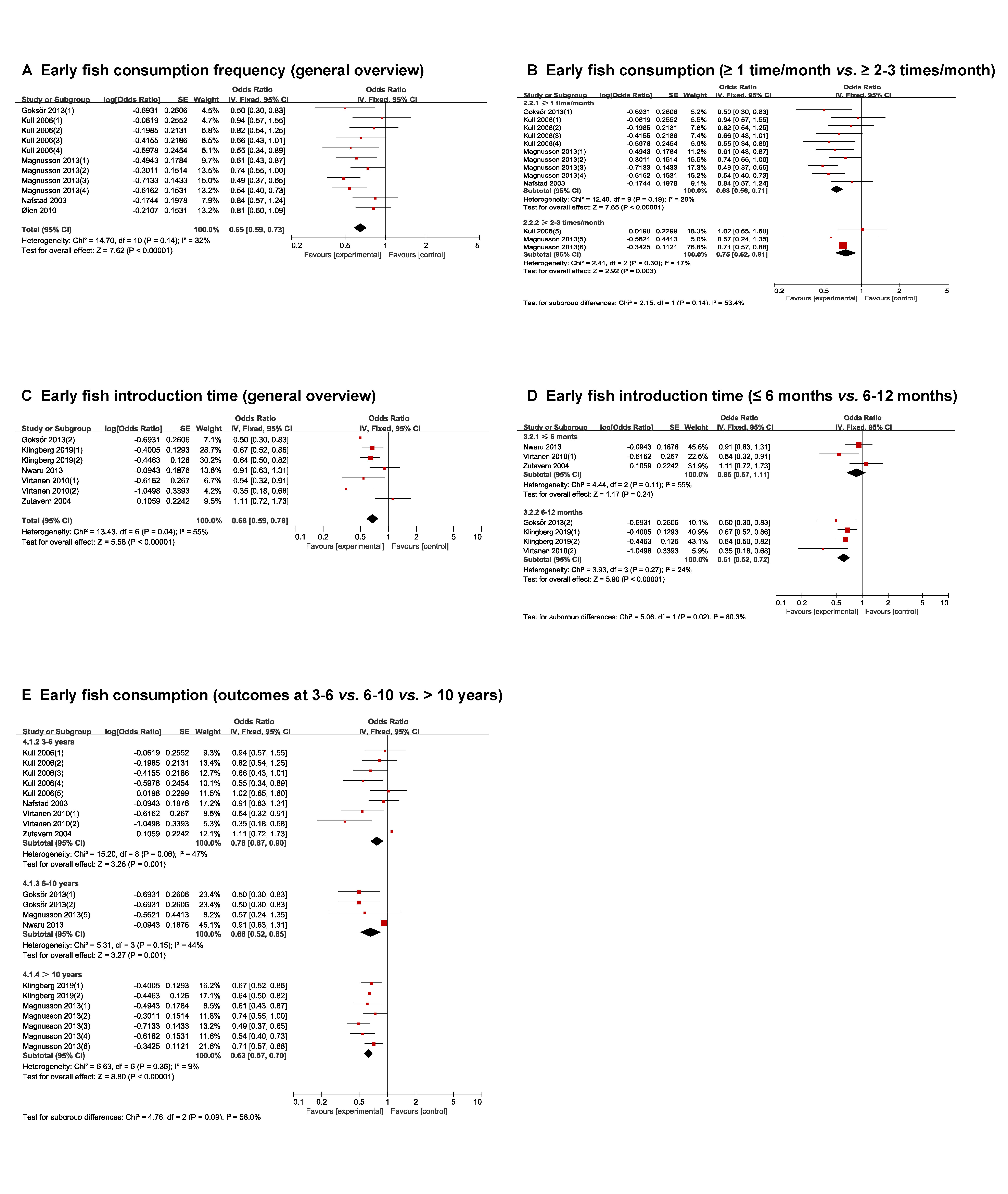
**Supplemental Figure 11. Effect of** **fish consumption on risk of asthma.** Effects of early fish intake frequency (A), specific frequency of fish consumption (B), period of early fish intake (C), specific timing of fish introduction (D), and time for outcome assessment (E).


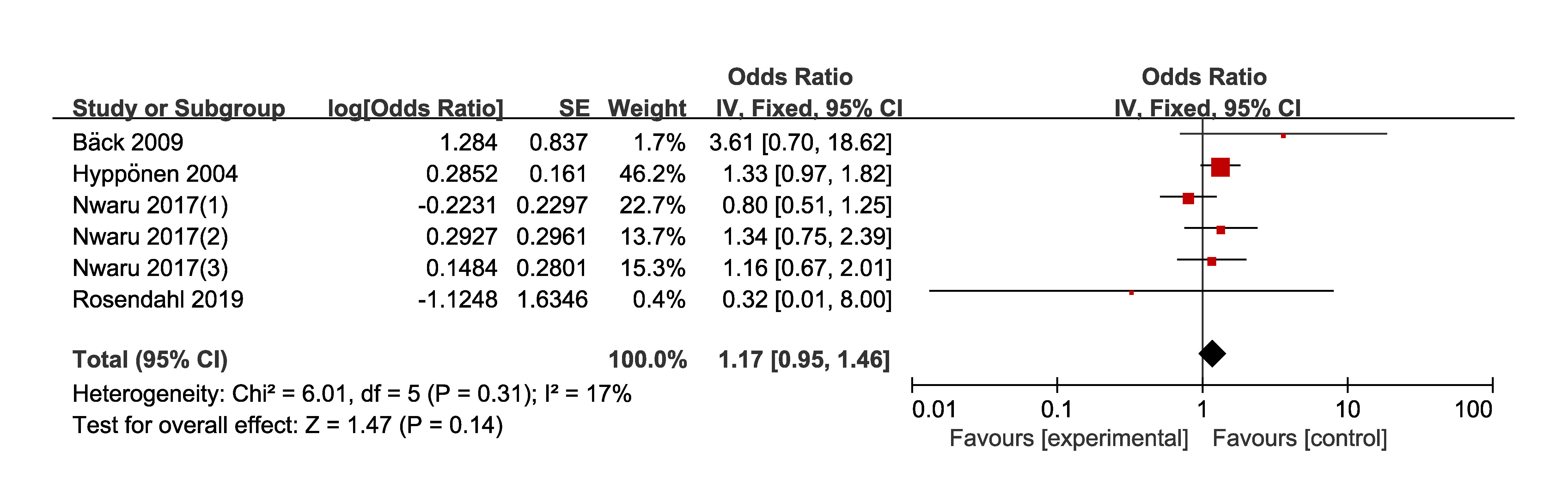
**Supplemental Figure 12. Effect of high-dose vitamin D supplementation on risk of asthma.**


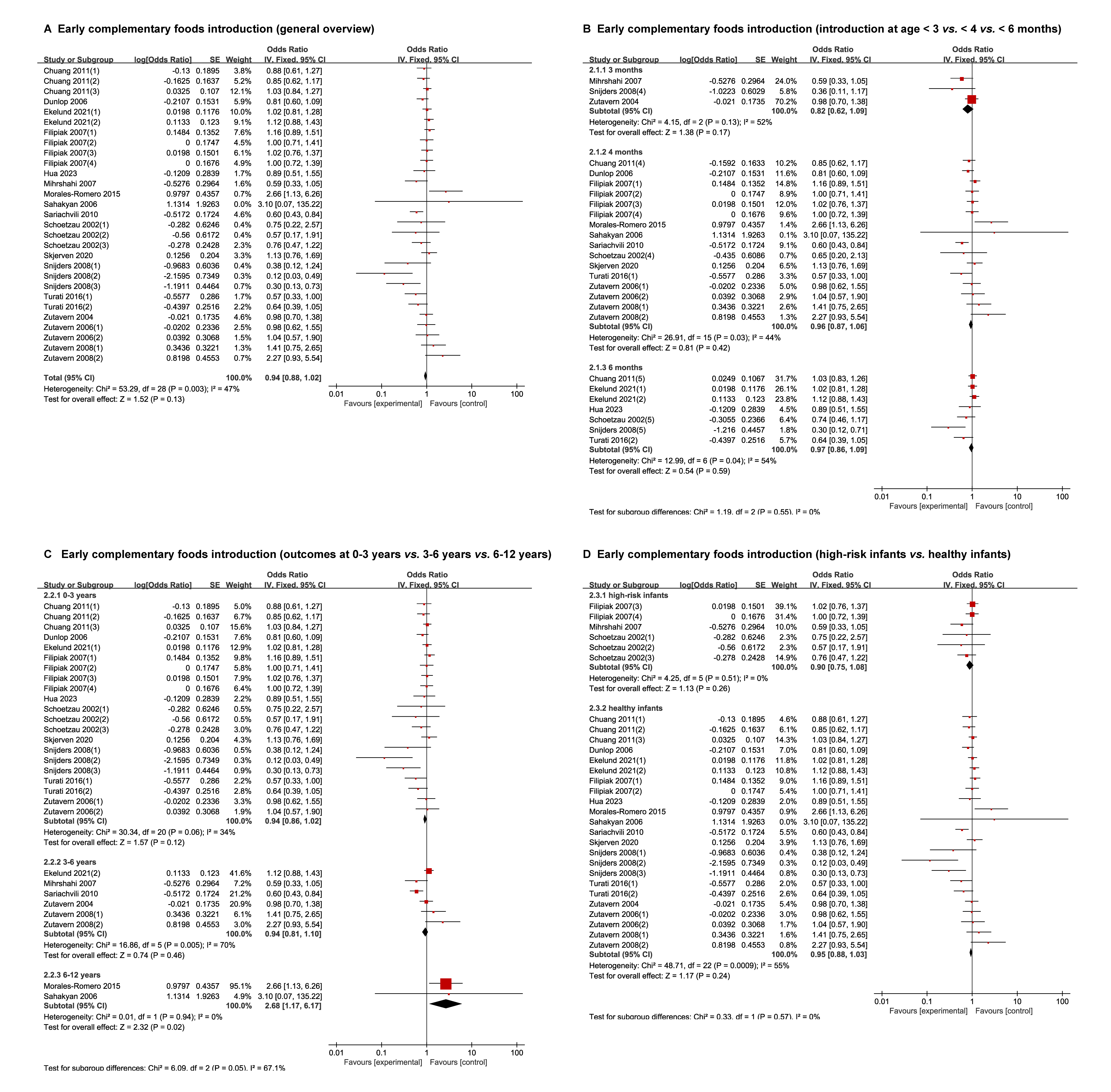
 **Supplemental Figure 13. Effect of early *vs.* late intake of complementary foods on risk of atopic dermatitis.** Effects on all participants (A). Effect of specific timing of intake (B) and time for outcome assessment (C). Effects on infants at high/normal risk of allergy (D).


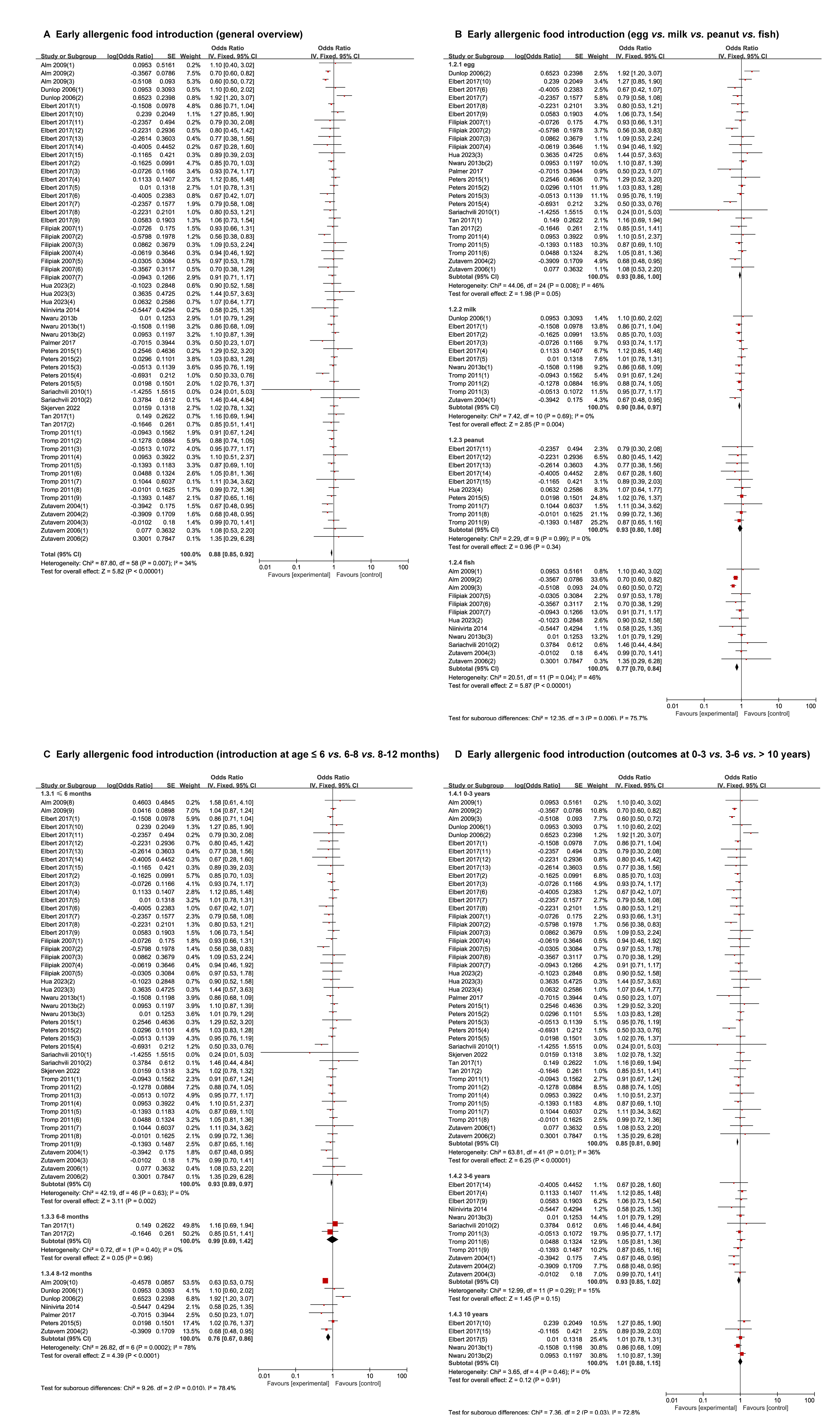


**Supplemental Figure 14. Effect of early *vs.* late intake of allergenic food on risk of atopic dermatitis.** Effects on all participants (A). Effect of specific allergenic food (B), specific timing of introduction (C), and time for outcome assessment (D).


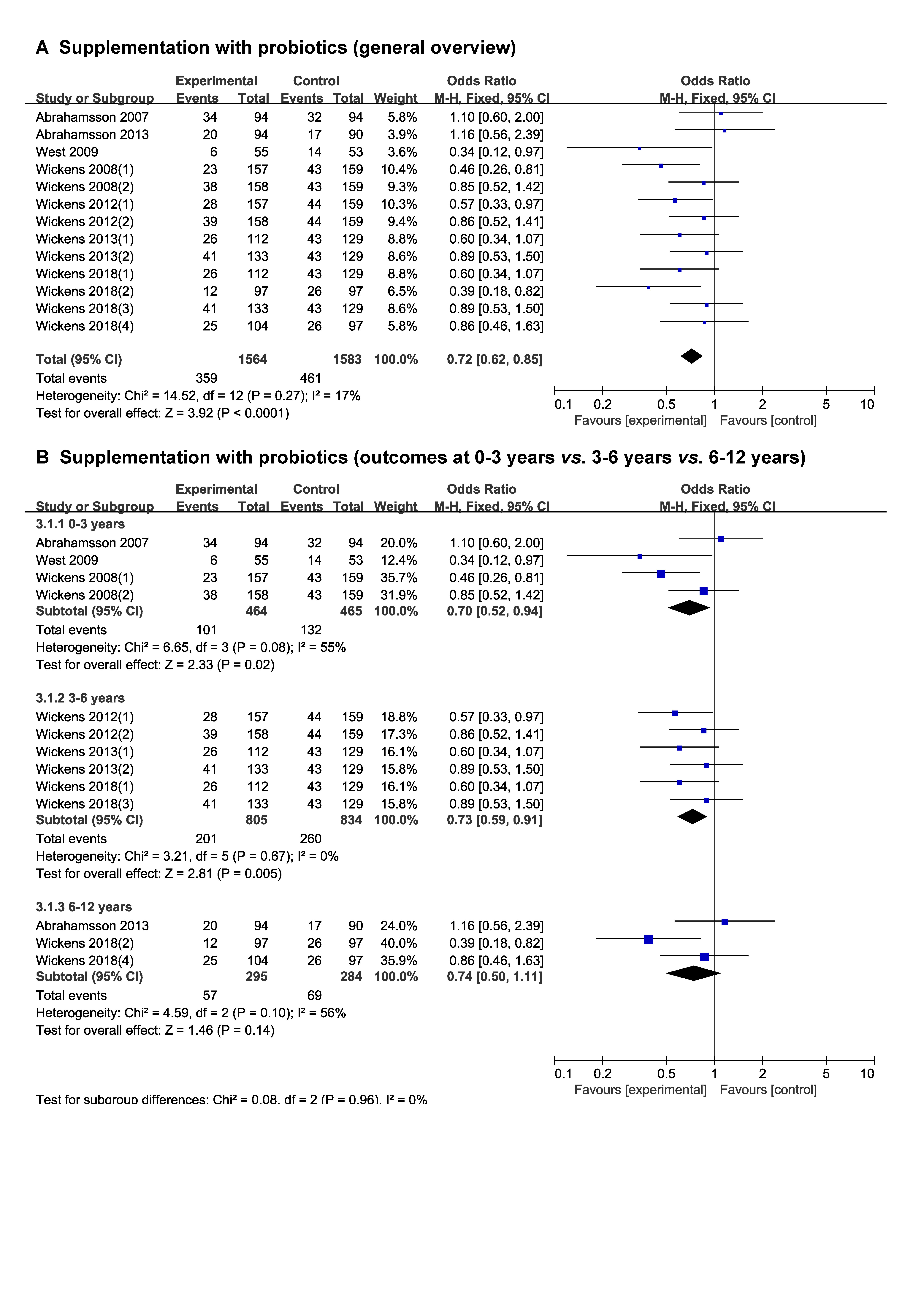


**Supplemental Figure 15. Effect of probiotic supplementation during the complementary food period on risk of atopic dermatitis.** Effects on all participants (A) and time for outcome assessment (B).


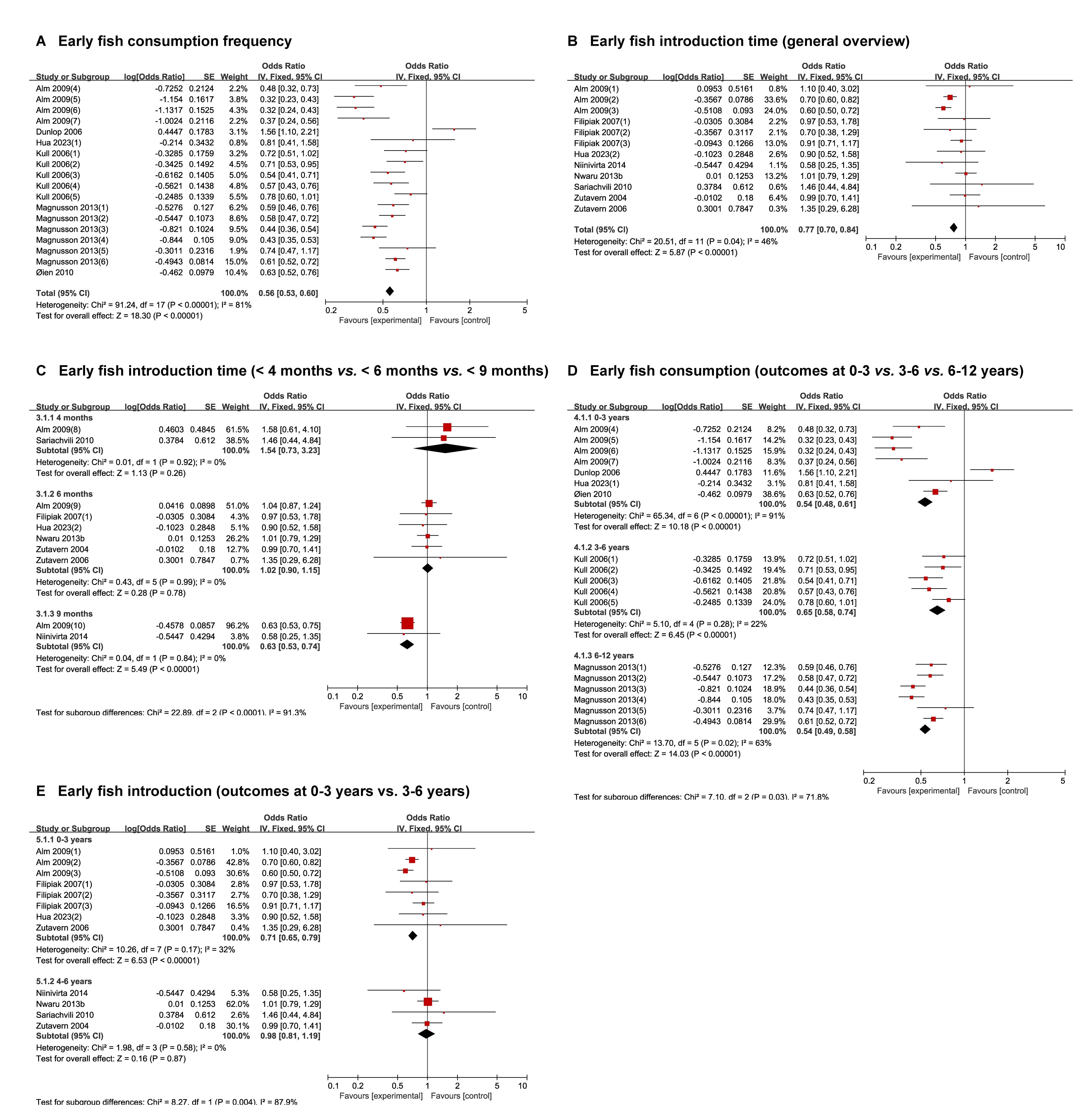


**Supplemental Figure 16. Effect of** **fish consumption on risk of atopic dermatitis.** Effects of early fish intake (A), early fish intake time (B), specific timing of fish consumption (C), time for outcome assessment of early fish consumption (D), and time for outcome assessment of early fish introduction (E).

**
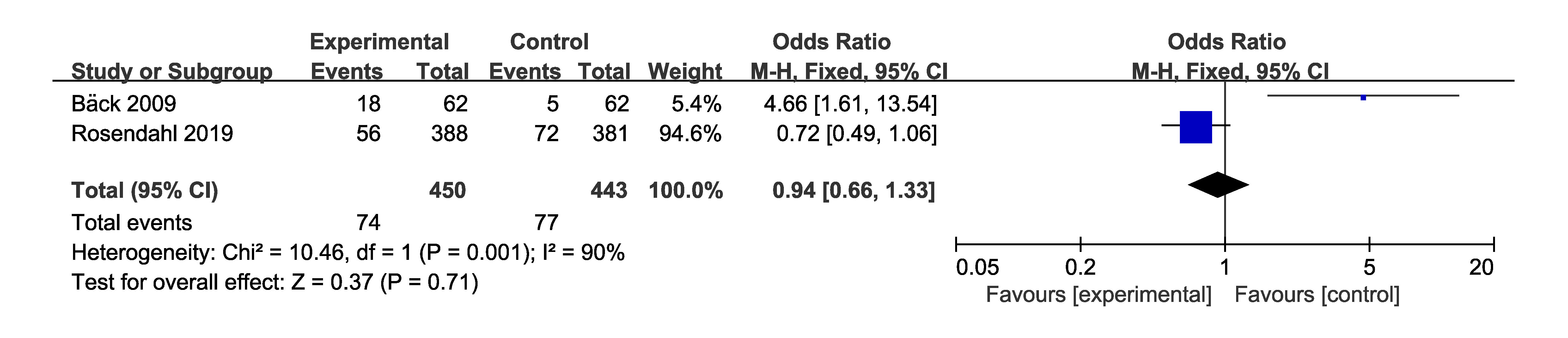
**

**Supplemental Figure 17. Effect of high-dose vitamin D supplementation on risk of atopic dermatitis.**

**Supplemental References**

1. Julia V, Macia L, Dombrowicz D. The impact of diet on asthma and allergic diseases. *Nat Immunol.* 2015;15(5):308-322.
2. Yamamoto T, Endo Y, Onodera A, et al. DUSP10 constrains innate IL-33-mediated cytokine production in ST2(hi) memory-type pathogenic Th2 cells. *Nat Commun*. 2018;9(1):4231.
3. Vos T, Lim SS, Abbafati C, et al. Global burden of 369 diseases and injuries in 204 countries and territories, 1990–2019: A systematic analysis for the Global Burden of Disease Study 2019. *Lancet* 2020;396(10258):1204-1222.
4. Fuchs O, Bahmer T, Rabe KF, von Mutius E. Asthma transition from childhood into adulthood. *Lancet Resp Med*. 2017;5(3):224-234.
5. Dowling DJ, Levy O. Ontogeny of early life immunity. *Trends Immunol.* 2014;35(7):299-310.
6. Perkin MR. Early introduction of allergenic food for all infants. *Lancet* 2022;399(10344):2329-2331.
7. Perkin MR, Bahnson HT, Logan K, et al. Factors influencing adherence in a trial of early introduction of allergenic food. *J Allergy Clin Immun*. 2019;144(6):1595-1605.
8. Wei-Liang Tan J, Valerio C, Barnes EH, et al. A randomized trial of egg introduction from 4 months of age in infants at risk for egg allergy. *J Allergy Clin Immun*. 2017;139(5):1621-1628.e8.
9. Perkin MR, Logan K, Tseng A, et al. Randomized trial of introduction of allergenic foods in breast-fed infants. *N Engl J Med.* 2016;374(18):1733-1743.
10. Clausen M, Jonasson K, Keil T, Beyer K, Sigurdardottir ST. Fish oil in infancy protects against food allergy in Iceland-Results from a birth cohort study. *Allergy* 2018;73(6):1305-1312.
11. Magnusson J, Kull I, Rosenlund H, et al. Fish consumption in infancy and development of allergic disease up to age 12 y. *Am J Clin Nutr.* 2013;97(6):1324-1330.
12. Vasileiadou S, Wennergren G, Strömberg Celind F, et al. Eating fish and farm life reduce allergic rhinitis at the age of twelve. *Pediat Allerg Imm.* 2018;29(3):283-289.
13. Tham EH, Lee BW, Chan YH, et al. Low food allergy prevalence despite delayed introduction of allergenic foods-data from the GUSTO cohort. *J Allergy Clin Immun*. 2018;6(2):466-475.e1.
14. Palmer DJ, Sullivan TR, Gold MS, Prescott SL, Makrides M. Randomized controlled trial of early regular egg intake to prevent egg allergy. *J Allergy Clin Immun.* 2017;139(5):1600-1607.e2.
15. Zutavern A, Brockow I, Schaaf B, et al. Timing of solid food introduction in relation to atopic dermatitis and atopic sensitization: Results from a prospective birth cohort study. *Pediatrics* 2006;117(2):401-411.
16. Fewtrell M, Bronsky J, Campoy C, et al. Complementary Feeding: A position paper by the European Society for Paediatric Gastroenterology, Hepatology, and Nutrition (ESPGHAN) committee on nutrition. *J Pediatr Gastr Nutr.* 2017;64(1):119-1132.
17. Page MJ, McKenzie JE, Bossuyt PM, et al. The PRISMA 2020 statement: an updated guideline for reporting systematic reviews. *Syst Rev.* 2021;10(1):1-11.
18. Panel N-SE. Guidelines for the diagnosis and management of food allergy in the United States: Report of the NIAID-sponsored expert panel. *J Allergy Clin Immun.* 2010;126(6):S1-58.
19. Ierodiakonou D, Garcia-Larsen V, Logan A, et al. Timing of allergenic food introduction to the infant diet and risk of allergic or autoimmune disease: A systematic review and meta-analysis. *JAMA.* 2016;316(11):1181-1192.
20. Egger M, Smith GD, Schneider M, Minder C. Bias in meta-analysis detected by a simple, graphical test. *BMJ.* 1997;315(7109):629-634.
21. Higgins JP, Altman DG, Gøtzsche PC, et al. The Cochrane Collaboration’s tool for assessing risk of bias in randomised trials. *BMJ.* 2011;343:d5928.
22. Sterne JA, Hernán MA, Reeves BC, et al. ROBINS-I: A tool for assessing risk of bias in non-randomised studies of interventions. *BMJ.* 2016;355:i4919.
23. Higgins JP, Thompson SG. Quantifying heterogeneity in a meta‐analysis. *Stat Med.* 2002;21(11):1539-1558.
24. Balshem H, Helfand M, Schünemann HJ, et al. GRADE guidelines: 3. Rating the quality of evidence. *J Clin Epidemiol.* 2011;64(4):401-406.
25. Skjerven HO, Lie A, Vettukattil R, et al. Early food intervention and skin emollients to prevent food allergy in young children (PreventADALL): A factorial, multicentre, cluster-randomised trial. *Lancet* 2022;399(10344):2398-2411.
26. Skjerven HO, Rehbinder EM, Vettukattil R, et al. Skin emollient and early complementary feeding to prevent infant atopic dermatitis (PreventADALL): A factorial, multicentre, cluster-randomised trial. *Lancet* 2020;395(10228):951-961.
27. Williams H, Jburney P, Pembroke A, Hay R, Party ADDCW. The UK Working Party's diagnostic criteria for atopic dermatitis. III. Independent hospital validation. *Brit J Dermatol.* 1994;131(3):406-416.
28. Hanifin JM, Lobitz WC. Newer concepts of atopic dermatitis. *Arch Dermatol.* 1977;113(5):663-670.
29. Schmidt RM, Pilmann Laursen R, Bruun S, et al. Probiotics in late infancy reduce the incidence of eczema: A randomized controlled trial. *Pediat Allerg Imm.* 2019;30(3):335-340.
30. Rosendahl J, Pelkonen AS, Helve O, et al. High-dose vitamin D supplementation does not prevent allergic sensitization of infants. *J Pediatr.* 2019;209:139-145.e1.
31. Wickens K, Barthow C, Mitchell EA, et al. Effects of *Lactobacillus rhamnosus* HN001 in early life on the cumulative prevalence of allergic disease to 11 years. *Pediat Allerg Imm.* 2018;29(8):808-814.
32. Wickens K, Stanley TV, Mitchell EA, et al. Early supplementation with *Lactobacillus rhamnosus* HN001 reduces eczema prevalence to 6 years: Does it also reduce atopic sensitization? *Clin exp allergy.* 2013;43(9):1048-1057.
33. Wickens K, Black P, Stanley TV, et al. A protective effect of *Lactobacillus rhamnosus* HN001 against eczema in the first 2 years of life persists to age 4 years. *Clin exp allergy*. 2012;42(7):1071-1079.
34. Wickens K, Black PN, Stanley TV, et al. A differential effect of 2 probiotics in the prevention of eczema and atopy: A double-blind, randomized, placebo-controlled trial. *J Allergy Clin Immun.* 2008;122(4):788-794.
35. Palmer DJ, Metcalfe J, Makrides M, et al. Early regular egg exposure in infants with eczema: A randomized controlled trial. *J Allergy Clin Immun.* 2013;132(2):387-392.e1.
36. Bellach J, Schwarz V, Ahrens B, et al. Randomized placebo-controlled trial of hen's egg consumption for primary prevention in infants. *J Allergy Clin Immun.* 2017;139(5):1591.
37. West CE, Hammarstrom ML, Hernell O. Probiotics in primary prevention of allergic disease-follow-up at 8-9 years of age. *Allergy* 2013;68(8):1015-1020.
38. West CE, Hammarstrom M-L, Hernell O. Probiotics during weaning reduce the incidence of eczema. *Pediat Allerg Imm.* 2009;20(5):430-437.
39. Abrahamsson TR, Jakobsson T, Björkstén B, Oldaeus G, Jenmalm MC. No effect of probiotics on respiratory allergies: A seven-year follow-up of a randomized controlled trial in infancy. *Pediat Allerg Imm*. 2013;24(6):556-561.
40. Abrahamsson TR, Jakobsson T, Bottcher MF, et al. Probiotics in prevention of IgE-associated eczema: A double-blind, randomized, placebo-controlled trial. *J Allergy Clin Immun.* 2007;119(5):1174-1180.
41. Chęsy M, Krogulska A. Introduction of complementary foods and the risk of sensitization and allergy in children up to three years of age. *Nutrients*. 2023;15(9):2054.
42. Hua MC, Yao TC, Liao SL, Tsai MH, Lai SH, Chen LC, et al. Introduction of egg white and yolk to infant diets and early childhood atopic dermatitis. *Nutrients*. 2023;15(6):1379.
43. Lu C, Zhang X, Liu Q, Li Q, Norbäck D, Deng Q. Effects of timing of complementary food introduction on childhood food allergy development: A modified role of ambient air pollution exposure. *Build Environ*. 2023;231:110065.
44. Wen X, Martone GM, Lehman HK, Rideout TC, Cameron CE, Dashley S, et al. Frequency of infant egg consumption and risk of maternal-reported egg allergy at 6 years. *J Nutr*. 2023;153(1):364-72.
45. Luccioli S, Zhang Y, Verrill L, Ramos-Valle M, Kwegyir-Afful E. Infant feeding practices and reported food allergies at 6 years of age. *Pediatrics* 2014;134(Suppl 1):S21-S28.
46. Obaid JMAS, Ali WAM, Al-Badani AFAM, et al. Early infant feeding and allergic respiratory diseases in Ibb city, Yemen. *Eur J Med Res.* 2022;27(1):35.
47. Adjibade M, Adjibade M, Davisse-Paturet C, et al. Enrichment of formula in probiotics or prebiotics and risk of infection and allergic diseases up to age 5.5 years in the Nationwide Etude Longitudinale Française depuis l'Enfance (ELFE) cohort. *The Journal of Nutrition*, 2022;152:1138-1148.
48. Yakaboski E, Robinson LB, Arroyo A, et al. Early introduction of food allergens and risk of developing food allergy. *Nutrients* 2021;13(7): 2318.
49. Ekelund L, Gloppen I, Øien T, Simpson MR. Duration of breastfeeding, age at introduction of complementary foods and allergy-related diseases: A prospective cohort study. *Int Breastfeed J.* 2021;16(1):5.
50. Oien T, Storrø O, Johnsen R. Do early intake of fish and fish oil protect against eczema and doctor-diagnosed asthma at 2 years of age? A cohort study. *J Epidemiol Commun H.* 2010;64(2):124-129.
51. Hose AJ, Pagani G, Karvonen AM, et al. Excessive unbalanced meat consumption in the first year of life increases asthma risk in the PASTURE and LUKAS2 birth cohorts. *Front Immunol.* 2021:1242.
52. Venter C, Maslin K, Holloway JW, et al. Different measures of diet diversity during infancy and the association with childhood food allergy in a UK birth cohort study. *J Allergy Clin Immun.* 2020;8(6):2017-2026.
53. Thorisdottir B, Gunnarsdottir I, Vidarsdottir AG, Sigurdardottir S, Birgisdottir BE, Thorsdottir I. Infant feeding, vitamin D and IgE sensitization to food allergens at 6 years in a longitudinal Icelandic cohort. *Nutrients* 2019;11(7):1690.
54. Klingberg S, Brekke HK, Ludvigsson J. Introduction of fish and other foods during infancy and risk of asthma in the All Babies In Southeast Sweden cohort study. *Eur J Pediatr.* 2019;178(3):395-402.
55. Loo EXL, Sim JZT, Toh JY, et al. Relation of infant dietary patterns to allergic outcomes in early childhood. *Pediat Allerg Imm.* 2017;28(5):490-495.
56. Lossius AK, Magnus MC, Lunde J, Stordal K. Prospective cohort study of breastfeeding and the risk of childhood asthma. [*J Pediatr.*](https://www.ablesci.com/journal/detail?id=5dk1Qp) 2018;195:182-189.e2.
57. Nwaru BI, Hadkhale K, Hämäläinen N, et al. Vitamin D intake during the first 4 years and onset of asthma by age 5: A nested case-control study. *Pediat Allerg Imm*. 2017;28(7):641-648.
58. Nwaru BI, Takkinen H-M, Kaila M, et al. Food diversity in infancy and the risk of childhood asthma and allergies. *J Allergy Clin Immun.* 2014;133(4):1084-1091.
59. Nwaru BI, Takkinen HM, Niemela O, et al. Introduction of complementary foods in infancy and atopic sensitization at the age of 5 years: Timing and food diversity in a Finnish birth cohort. *Allergy* 2013;68(4):507-516.
60. Nwaru BI, Erkkola M, Ahonen S, et al. Age at the introduction of solid foods during the first year and allergic sensitization at age 5 years. *Pediatrics* 2010;125(1):50-59.
61. Virtanen SM, Kaila M, Pekkanen J, et al. Early introduction of oats associated with decreased risk of persistent asthma and early introduction of fish with decreased risk of allergic rhinitis. *Br J Nutr.* 2010;103(2):266-273.
62. Elbert NJ, Kiefte-de Jong JC, Voortman T, et al. Allergenic food introduction and risk of childhood atopic diseases. *PloS one* 2017;12(11): e0187999.
63. Turati F, Bertuccio P, Galeone C, et al. Early weaning is beneficial to prevent atopic dermatitis occurrence in young children. *Allergy* 2016;71(6):878-888.
64. Gabet S, Just J, Couderc R, Seta N, Momas I. Allergic sensitisation in early childhood: Patterns and related factors in PARIS birth cohort. *Int J Hyg Environ Health.* 2016;219(8):792-800.
65. Peters RL, Allen KJ, Dharmage SC, et al. Differential factors associated with challenge-proven food allergy phenotypes in a population cohort of infants: A latent class analysis. *Clin Exp Allergy.* 2015;45(5):953-963.
66. Morales-Romero J, Bedolla-Barajas M, Lopez-Vargas L, Enrique Romero-Velarde C. Prevalence of allergic diseases and their association with breastfeeding and initiation of complementary feeding in school-age children of Ciudad Guzman, *Mexico. Arch Argent Pediatr.* 2015;113(4):324-330.
67. Roduit C, Frei R, Depner M, et al. Increased food diversity in the first year of life is inversely associated with allergic diseases. *J Allergy Clin Immun.* 2014;133(4):1056-1064.
68. Niinivirta K, Isolauri E, Nermes M, Laitinen K. Timing of complementary feeding and the risk of atopic eczema. *Acta Paediatrica* 2014;103(2):168-173.
69. Grimshaw KE, Maskell J, Oliver EM, et al. Diet and food allergy development during infancy: birth cohort study findings using prospective food diary data. *J Allergy Clin Immun.* 2014;133(2):511-519.
70. Nwaru BI, Craig LC, Allan K, et al. Breastfeeding and introduction of complementary foods during infancy in relation to the risk of asthma and atopic diseases up to 10 years. *Clin Exp Allergy.* 2013;43(11):1263-1273.
71. Goksör E, Alm B, Pettersson R, et al. Early fish introduction and neonatal antibiotics affect the risk of asthma into school age. *Pediat Allerg Imm*. 2013;24(4):339-344.
72. Alm B, Goksör E, Thengilsdottir H, et al. Early protective and risk factors for allergic rhinitis at age 4½ yr. *Pediat Allerg Imm*. 2011;22(4):398-404.
73. Alm B, Aberg N, Erdes L, et al. Early introduction of fish decreases the risk of eczema in infants. *Arch Dis Child.* 2009;94(1):11-15.
74. Tromp, II, Kiefte-de Jong JC, de Vries JH, et al. Dietary patterns and respiratory symptoms in pre-school children: the Generation R Study. *Eur Respir J.* 2012;40(3):681-689.
75. Tromp IIM, Kiefte-de Jong JC, Lebon A, et al. The introduction of allergenic foods and the development of reported wheezing and eczema in childhood: The generation R study. *Arch Pediat Adol Med.* 2011;165(10):933-938.
76. GINIplus, Sibylle LSGSSssh-mdHJK. Early diet and the risk of allergy: What can we learn from the prospective birth cohort studies GINIplus and LISAplus? *Am J Clin Nutr.* 2011;94(Suppl 6):2012S-2017S.
77. Chuang C-H, Hsieh W-S, Chen Y-C, et al. Infant feeding practices and physician diagnosed atopic dermatitis: A prospective cohort study in Taiwan. *Pediat Allerg Imm*. 2011;22(1):43-49.
78. Sariachvili M, Droste J, Dom S, et al. Early exposure to solid foods and the development of eczema in children up to 4 years of age. *Pediat Allerg Imm*. 2010;21(1):74-81.
79. Hetzner NM, Razza RA, Malone LM, Brooks-Gunn J. Associations among feeding behaviors during infancy and child illness at two years. *Maternal and child health journal* 2009;13(6):795-805.
80. Back O, Bloomquist HKS, Hernell O, Stenberg B. Does vitamin D Intake during infancy promote the development of atopic allergy? *Acta dermato-venereol.* 2009;89(1):28-32.
81. Zutavern A, Brockow I, Schaaf B, et al. Timing of solid food introduction in relation to eczema, asthma, allergic rhinitis, and food and inhalant sensitization at the age of 6 years: Results from the prospective birth cohort study LISA. *Pediatrics* 2008;121(1):E44-52.
82. Snijders BE, Thijs C, van Ree R, van den Brandt PA. Age at first introduction of cow milk products and other food products in relation to infant atopic manifestations in the first 2 years of life: The KOALA Birth Cohort Study. *Pediatrics* 2008;122(1):e115-122.
83. Mihrshahi S, Ampon R, Webb K, et al. The association between infant feeding practices and subsequent atopy among children with a family history of asthma. *Clin Exp Allergy.* 2007;37(5):671-679.
84. Filipiak B, Zutavern A, Koletzko S, et al. Solid food introduction in relation to eczema: results from a four-year prospective birth cohort study. *J Pediatr.* 2007;151(4):352-358.
85. Kull I, Bergstrom A, Lilja G, Pershagen G, Wickman M. Fish consumption during the first year of life and development of allergic diseases during childhood. *Allergy* 2006;61(8):1009-1015.
86. Sahakyan A, Armenian HK, Breitscheidel L, Thompson ME, Enokyan G. Feeding practices of babies and the development of atopic dermatitis in children after 12 months of age in Armenia: Is there a signal? *Eur J Epidemiol.* 2006;21(9):723-725.
87. Dunlop AL, Reichrtova E, Palcovicova L, et al. Environmental and dietary risk factors for infantile atopic eczema among a Slovak birth cohort. *Pediat Allerg Imm*. 2006;17(2):103-111.
88. Hyppönen E, Sovio U, Wjst M, et al. Infant vitamin d supplementation and allergic conditions in adulthood: Northern Finland birth cohort 1966. *Ann N Y Acad Sci.* 2004;1037(1):84-95.
89. Zutavern A, von Mutius E, Harris J, et al. The introduction of solids in relation to asthma and eczema. *Arch Dis Child*. 2004;89(4):303-308.
90. Nafstad P, Nystad W, Magnus P, Jaakkola JJ. Asthma and allergic rhinitis at 4 years of age in relation to fish consumption in infancy. *J Asthma.* 2003;40(4):343-348.
91. Schoetzau A, Filipiak-Pittroff B, Franke K, et al. Effect of exclusive breast-feeding and early solid food avoidance on the incidence of atopic dermatitis in high-risk infants at 1 year of age. *Pediat Allerg Imm*. 2002;13(4):234-242.
92. Obbagy JE, English LK, Wong YP, et al. Complementary feeding and food allergy, atopic dermatitis/eczema, asthma, and allergic rhinitis: a systematic review. *Am J Clin Nutr.* 2019;109(Suppl 1):890S-934S.
93. Venter C, Greenhawt M, Meyer RW, et al. EAACI position paper on diet diversity in pregnancy, infancy and childhood: Novel concepts and implications for studies in allergy and asthma. *Allergy* 2020;75(3):497-523.
94. Miles EA, Calder PC. Omega-6 and omega-3 polyunsaturated fatty acids and allergic diseases in infancy and childhood. *Curr Pharm Des*. 2014;20(6):946-953.
95. Strachan DP. Family size, infection and atopy: The first decade of the 'hygiene hypothesis'. Thorax 2000;55(Suppl 1):S2-10.
96. Zimmermann P, Messina N, Mohn WW, Finlay BB, Curtis N. Association between the intestinal microbiota and allergic sensitization, eczema, and asthma: A systematic review. *J Allergy Clin Immun.* 2019;143(2):467-485.
97. Allen KJ, Koplin JJ, Ponsonby AL, et al. Vitamin D insufficiency is associated with challenge-proven food allergy in infants. *J Allergy Clin Immun.* 2013;131(4):1109-1116.
98. Brehm JM, Schuemann B, Fuhlbrigge AL, et al. Serum vitamin D levels and severe asthma exacerbations in the Childhood Asthma Management Program study. *J Allergy Clin Immun.* 2010;126(1):52-58.e5.
99. Wang SS, Hon KL, Kong AP, Pong HN, Wong GW, Leung TF. Vitamin D deficiency is associated with diagnosis and severity of childhood atopic dermatitis. *Pediat Allerg Imm*. 2014;25(1):30-35.

**Supplemental Appendix 1. Search Strategies**

The search strategies included both text terms and subject heading terms where appropriate. Since ‘Hypersensitivity’ may include ‘Asthma’ and ‘Atopic dermatitis’, and the evaluation outcomes of a cohort study may include food allergy, asthma, and atopic dermatitis, therefore, the other two topics were considered in the selection of publications retrieved under each topic. We searched the following databases:

**1.1 The Cochrane Library**

**Complementary feeding & Hypersensitivity**

#1 (complementary feeding):ti,ab,kw (Word variations have been searched)

#2 (diet):ti,ab,kw OR (foods):ti,ab,kw OR (food):ti,ab,kw OR (Infant Nutritional Physiology):ti,ab,kw OR (Nutritional Physiology, Infant):ti,ab,kw OR (Physiology, Infant Nutritional):ti,ab,kw OR (Infant Nutritional Physiological Phenomenon):ti,ab,kw OR (Infant Nutrition Physiology):ti,ab,kw OR (Physiology, Infant Nutrition):ti,ab,kw OR (Nutrition Physiology, Infant):ti,ab,kw OR (Supplementary Feeding):ti,ab,kw OR (Feeding, Supplementary):ti,ab,kw OR (Feedings, Supplementary):ti,ab,kw OR (Supplementary Feedings):ti,ab,kw OR (Complementary Feeding):ti,ab,kw OR (Complementary Feedings):ti,ab,kw OR (Feeding, Complementary):ti,ab,kw OR (Feedings, Complementary):ti,ab,kw (Word variations have been searched)

#3 #1 or #2

#4 MeSH descriptor: [Infant Nutritional Physiological Phenomena] explode all trees

#5 #3 or #4

#6 (Hypersensitivity):ti,ab,kw OR (Drug Hypersensitivity):ti,ab,kw OR (Nut Hypersensitivity):ti,ab,kw OR (Peanut Hypersensitivity):ti,ab,kw OR (Wheat Hypersensitivity):ti,ab,kw OR (Egg Hypersensitivity):ti,ab,kw OR (Latex Hypersensitivity):ti,ab,kw OR (Milk Hypersensitivity):ti,ab,kw OR (Respiratory Hypersensitivity):ti,ab,kw OR (Food Hypersensitivity):ti,ab,kw OR (Shellfish Hypersensitivity):ti,ab,kw OR (Cold Hypersensitivity):ti,ab,kw (Word variations have been searched)

#7 MeSH descriptor: [Hypersensitivity] explode all trees

#8 #6 or #7

#9 (Randomized controlled trial):ti,ab,kw OR (Non–randomized controlled trial):ti,ab,kw OR (Prospective cohort study):ti,ab,kw OR (Retrospective cohort study):ti,ab,kw OR (Case-control study):ti,ab,kw OR (Pre study control):ti,ab,kw OR (post study control):ti,ab,kw (Word variations have been searched)

#10 MeSH descriptor: [Randomized Controlled Trial] explode all trees

#11 #9 or #10

#12 #5 and #8 and #11

**Complementary feeding & Asthma**

#1 (Asthma):ti,ab,kw (Word variations have been searched)

#2 (Bronchial):ti,ab,kw (Word variations have been searched)

#3 (Bronchial asthma):ti,ab,kw (Word variations have been searched)

#4 #1 or #2 or #3

#5 MeSH descriptor: [Asthma] explode all trees

#6 #4 or #5

#7 (Complementary feeding):ti,ab,kw OR (Infant Nutritional Physiology):ti,ab,kw OR (Nutritional Physiology, Infant):ti,ab,kw OR (Physiology, Infant Nutritional):ti,ab,kw OR (Infant Nutritional Physiological Phenomenon):ti,ab,kw (Word variations have been searched)

#8 (Infant Nutrition Physiology):ti,ab,kw OR (Physiology, Infant Nutrition):ti,ab,kw OR (Nutrition Physiology, Infant):ti,ab,kw OR (Supplementary Feeding):ti,ab,kw OR (Feeding, Supplementary):ti,ab,kw (Word variations have been searched)

#9 (Feedings, Supplementary):ti,ab,kw OR (Supplementary Feedings):ti,ab,kw OR (Complementary Feeding):ti,ab,kw OR (Complementary Feedings):ti,ab,kw OR (Feeding, Complementary):ti,ab,kw (Word variations have been searched)

#10 (Feedings, Complementary):ti,ab,kw OR (Diet):ti,ab,kw OR (foods):ti,ab,kw (Word variations have been searched)

#11 MeSH descriptor: [Infant Nutritional Physiological Phenomena] explode all trees

#12 #7 or #8 or #9 or #10 or #11

#13 (Randomized controlled trial):ti,ab,kw OR (Non–randomized controlled trial):ti,ab,kw OR (Prospective cohort study):ti,ab,kw (Word variations have been searched)

#14 (Retrospective cohort study):ti,ab,kw OR (Case-control study):ti,ab,kw OR (Pre study control):ti,ab,kw (Word variations have been searched)

#15 (post study control):ti,ab,kw (Word variations have been searched)

#16 #13 or #14 or #15

#17 MeSH descriptor: [Randomized Controlled Trial] explode all trees

#18 #16 or #17

#19 #6 and #12 and #18

**Complementary feeding & Atopic dermatitis**

#1 (Complementary feeding):ti,ab,kw OR (Infant Nutritional Physiology):ti,ab,kw OR (Nutritional Physiology, Infant):ti,ab,kw OR (Physiology, Infant Nutritional):ti,ab,kw OR (Infant Nutritional Physiological Phenomenon):ti,ab,kw (Word variations have been searched)

#2 (Infant Nutrition Physiology):ti,ab,kw OR (Physiology, Infant Nutrition):ti,ab,kw OR (Nutrition Physiology, Infant):ti,ab,kw OR (Supplementary Feeding):ti,ab,kw OR (Feeding, Supplementary):ti,ab,kw (Word variations have been searched)

#3 (Feedings, Supplementary):ti,ab,kw OR (Supplementary Feedings):ti,ab,kw OR (Complementary Feeding):ti,ab,kw OR (Complementary Feedings):ti,ab,kw OR (Feeding, Complementary):ti,ab,kw (Word variations have been searched)

#4 (Feedings, Complementary):ti,ab,kw OR (Diet):ti,ab,kw OR (Food):ti,ab,kw (Word variations have been searched)

#5 MeSH descriptor: [Infant Nutritional Physiological Phenomena] explode all trees

#6 #1 OR #2 OR #3 OR #4 OR #5

#7 (Atopic Dermatitides):ti,ab,kw OR (Atopic Dermatitis):ti,ab,kw OR (Dermatitides, Atopic):ti,ab,kw OR (Neurodermatitis, Atopic):ti,ab,kw OR (Atopic Neurodermatitides):ti,ab,kw OR (Atopic Neurodermatitis):ti,ab,kw OR (Neurodermatitides, Atopic):ti,ab,kw OR (Neurodermatitis, Disseminated):ti,ab,kw OR (Disseminated Neurodermatitides):ti,ab,kw (Word variations have been searched)

#8 (Disseminated Neurodermatitis):ti,ab,kw OR (Neurodermatitides, Disseminated):ti,ab,kw OR (Eczema, Atopic):ti,ab,kw OR (Atopic Eczema):ti,ab,kw OR (Eczema, Infantile):ti,ab,kw (Word variations have been searched)

#9 (Infantile Eczema):ti,ab,kw OR (Eczema):ti,ab,kw (Word variations have been searched)

#10 MeSH descriptor: [Dermatitis, Atopic] explode all trees

#11 #7 OR #8 OR #9 OR #10

#12 (Randomized controlled trial):ti,ab,kw OR (Non–randomized controlled trial):ti,ab,kw OR (Prospective cohort study):ti,ab,kw OR (Retrospective cohort study):ti,ab,kw OR (Case-control study):ti,ab,kw (Word variations have been searched)

#13 (Pre study control):ti,ab,kw OR (post study control):ti,ab,kw (Word variations have been searched)

#14 MeSH descriptor: [Randomized Controlled Trial] explode all trees

#15 #12 OR #13 OR #14

#16 #6 and #11 and #15

**1.2 EMBASE**

**Complementary feeding & Hypersensitivity**

#1 ‘complementary feeding’:ti,ab,kw

#2 ‘diet’:ti,ab,kw OR ‘foods’:ti,ab,kw OR ‘food’:ti,ab,kw OR ‘Infant Nutritional Physiology’:ti,ab,kw OR ‘Nutritional Physiology, Infant’:ti,ab,kw OR ‘Physiology, Infant Nutritional’:ti,ab,kw OR ‘Infant Nutritional Physiological Phenomenon’:ti,ab,kw OR ‘Infant Nutrition Physiology’:ti,ab,kw OR ‘Physiology, Infant Nutrition’:ti,ab,kw OR ‘Nutrition Physiology, Infant’:ti,ab,kw OR ‘Supplementary Feeding’:ti,ab,kw OR ‘Feeding, Supplementary’:ti,ab,kw OR ‘Feedings, Supplementary’:ti,ab,kw OR ‘Supplementary Feedings’:ti,ab,kw OR ‘Complementary Feeding’:ti,ab,kw OR ‘Complementary Feedings’:ti,ab,kw OR ‘Feeding, Complementary’:ti,ab,kw OR ‘Feedings, Complementary’:ti,ab,kw

#3 #1 OR #2

#4 ‘Infant Nutritional Physiological Phenomena’/exp

#5 #3 OR #4

#6 ‘Hypersensitivity’:ti,ab,kw OR ‘Drug Hypersensitivity’:ti,ab,kw OR ‘Nut Hypersensitivity’:ti,ab,kw OR ‘Peanut Hypersensitivity’:ti,ab,kw OR ‘Wheat Hypersensitivity’:ti,ab,kw OR ‘Egg Hypersensitivity’:ti,ab,kw OR ‘Latex Hypersensitivity’:ti,ab,kw OR ‘Milk Hypersensitivity’:ti,ab,kw OR ‘Respiratory Hypersensitivity’:ti,ab,kw OR ‘Food Hypersensitivity’:ti,ab,kw OR ‘Shellfish Hypersensitivity’:ti,ab,kw OR ‘Cold Hypersensitivity’:ti,ab,kw

#7 ‘Hypersensitivity’/exp

#8 #6 OR #7

#9 ‘Randomized controlled trial’:ti,ab,kw OR ‘Non–randomized controlled trial’:ti,ab,kw OR ‘Prospective cohort study’:ti,ab,kw OR ‘Retrospective cohort study’:ti,ab,kw OR ‘Case-control study’:ti,ab,kw OR ‘Pre study control’:ti,ab,kw OR ‘post study control’:ti,ab,kw

#10 ‘Randomized Controlled Trial’/exp

#11 #9 OR #10

#12 #5 AND #8 AND #11

**Complementary feeding & Asthma**

#1‘Complementary feeding’:ti,ab,kw OR ‘Infant Nutritional Physiology’:ti,ab,kw OR ‘Nutritional Physiology, Infant’:ti,ab,kw OR ‘Physiology, Infant Nutritional’:ti,ab,kw OR ‘Infant Nutritional Physiological Phenomenon’:ti,ab,kw OR ‘Infant Nutrition Physiology’:ti,ab,kw OR ‘Physiology, Infant Nutrition’:ti,ab,kw OR ‘Nutrition Physiology, Infant’:ti,ab,kw OR ‘Supplementary Feeding’:ti,ab,kw OR ‘Feeding, Supplementary’:ti,ab,kw OR ‘Feedings, Supplementary’:ti,ab,kw OR ‘Supplementary Feedings’:ti,ab,kw OR ‘Complementary Feeding’:ti,ab,kw OR ‘Complementary Feedings’:ti,ab,kw OR ‘Feeding, Complementary’:ti,ab,kw OR ‘Feedings, Complementary’:ti,ab,kw OR ‘Diet’:ti,ab,kw OR ‘Food’:ti,ab,kw

#2 ‘Infant Nutritional Physiological Phenomena’/exp

#3 #1 OR #2

#4 ‘Asthma’:ti,ab,kw OR ‘Bronchial’:ti,ab,kw OR ‘Bronchial asthma’:ti,ab,kw’

#5 ‘Asthma’/exp

#6 #4 OR #5

#7 ‘Randomized controlled trial’:ti,ab,kw OR ‘Non–randomized controlled trial’:ti,ab,kw OR ‘Prospective cohort study’:ti,ab,kw

#8 ‘Retrospective cohort study’:ti,ab,kw OR ‘Case-control study’:ti,ab,kw OR‘Pre study control’:ti,ab,kw

#9 ‘post study control’:ti,ab,kw

#10 #7 OR #8 OR #9

#11 ‘Randomized Controlled Trial’/exp

#12 #10 OR #11

#13 #3 AND #6 AND #10 AND #12

**Complementary feeding & Atopic dermatitis**

#1 ‘Complementary feeding’:ti,ab,kw OR ‘Infant Nutritional Physiology’:ti,ab,kw OR ‘Nutritional Physiology, Infant’:ti,ab,kw OR ‘Physiology, Infant Nutritional’:ti,ab,kw OR ‘Infant Nutritional Physiological Phenomenon’:ti,ab,kw

#2 ‘Infant Nutrition Physiology’:ti,ab,kw OR ‘Physiology, Infant Nutrition’:ti,ab,kw OR ‘Nutrition Physiology, Infant’:ti,ab,kw OR ‘Supplementary Feeding’:ti,ab,kw OR ‘Feeding, Supplementary’:ti,ab,kw

#3 ‘Feedings, Supplementary’:ti,ab,kw OR ‘Supplementary Feedings’:ti,ab,kw OR ‘Complementary Feeding’:ti,ab,kw OR ‘Complementary Feedings’:ti,ab,kw OR ‘Feeding, Complementary’:ti,ab,kw

#4 ‘Feedings, Complementary’:ti,ab,kw OR ‘Diet’:ti,ab,kw OR ‘Food’:ti,ab,kw

#5 ‘Infant Nutritional Physiological Phenomena’/exp

#6 #1 OR #2 OR #3 OR #4 OR #5

#7 ‘Atopic Dermatitides’:ti,ab,kw OR ‘Atopic Dermatitis’:ti,ab,kw OR ‘Dermatitides, Atopic’:ti,ab,kw OR ‘Neurodermatitis, Atopic’:ti,ab,kw OR ‘Atopic Neurodermatitides’:ti,ab,kw OR ‘Atopic Neurodermatitis’:ti,ab,kw OR ‘Neurodermatitides, Atopic’:ti,ab,kw OR ‘Neurodermatitis, Disseminated’:ti,ab,kw OR ‘Disseminated Neurodermatitides’:ti,ab,kw

#8 ‘Disseminated Neurodermatitis’:ti,ab,kw OR ‘Neurodermatitides, Disseminated’:ti,ab,kw OR ‘Eczema, Atopic’:ti,ab,kw OR ‘Atopic Eczema’:ti,ab,kw OR ‘Eczema, Infantile’:ti,ab,kw

#9 ‘Infantile Eczema’:ti,ab,kw OR ‘Eczema’:ti,ab,kw

#10 ‘Dermatitis, Atopic’/exp

#11 #7 OR #8 OR #9 OR #10

#12 ‘Randomized controlled trial’:ti,ab,kw OR ‘Non–randomized controlled trial’:ti,ab,kw OR ‘Prospective cohort study’:ti,ab,kw OR ‘Retrospective cohort study’:ti,ab,kw OR ‘Case-control study’:ti,ab,kw ‘Word variations have been searched’

#13 ‘Pre study control’:ti,ab,kw OR ‘post study control’:ti,ab,kw

#14 ‘Randomized Controlled Trial’/exp

#15 #12 OR #13 OR #14

#16 #6 AND #11 AND #15

**1.3 Web of Science**

**Complementary feeding & Hypersensitivity**

#1 complementary feeding (Topic) or diet (Topic) or foods (Topic) or food (Topic) or Infant Nutritional Physiology (Topic) or Nutritional Physiology, Infant (Topic) or Physiology, Infant Nutritional (Topic) or Infant Nutritional Physiological Phenomenon (Topic) or Infant Nutrition Physiology (Topic) or Physiology, Infant Nutrition (Topic) or Nutrition Physiology, Infant (Topic) or Supplementary Feeding (Topic) or Feeding, Supplementary (Topic) or Feedings, Supplementary (Topic) or Supplementary Feedings (Topic) or Complementary Feeding (Topic) or Complementary Feedings (Topic) or Feeding, Complementary (Topic) or Feedings, Complementary (Topic)

#2 Hypersensitivity (Topic) or Drug Hypersensitivity (Topic) or Nut Hypersensitivity (Topic) or Peanut Hypersensitivity (Topic) or Wheat Hypersensitivity (Topic) or Egg Hypersensitivity (Topic) or Latex Hypersensitivity (Topic) or Milk Hypersensitivity (Topic) or Respiratory Hypersensitivity (Topic) or Food Hypersensitivity (Topic) or Shellfish Hypersensitivity (Topic) or Cold Hypersensitivity (Topic)

#3 Randomized controlled trial (Topic) or Non–randomized controlled trial (Topic) or Prospective cohort study (Topic) or Retrospective cohort study (Topic) or Case-control study (Topic) or Pre study control (Topic) or post study control (Topic)

#4 #1 AND#2 AND #3

**Complementary feeding & Asthma**

#1 complementary feeding (Topic) or diet (Topic) or foods (Topic) or food (Topic) or Infant Nutritional Physiology (Topic) or Nutritional Physiology, Infant (Topic) or Physiology, Infant Nutritional (Topic) or Infant Nutritional Physiological Phenomenon (Topic) or Infant Nutrition Physiology (Topic) or Physiology, Infant Nutrition (Topic) or Nutrition Physiology, Infant (Topic) or Supplementary Feeding (Topic) or Feeding, Supplementary (Topic) or Feedings, Supplementary (Topic) or Supplementary Feedings (Topic) or Complementary Feeding (Topic) or Complementary Feedings (Topic) or Feeding, Complementary (Topic) or Feedings, Complementary (Topic)

#2 Asthma (Topic) or Bronchial (Topic) or Bronchial asthma (Topic)

#3 Randomized controlled trial (Topic) or Non–randomized controlled trial (Topic) or Prospective cohort study (Topic) or Retrospective cohort study (Topic) or Case-control study (Topic) or Pre study control (Topic) or post study control (Topic)

#4 #1 AND#2 AND #3

**Complementary feeding & Atopic dermatitis**

#1 complementary feeding (Topic) or diet (Topic) or foods (Topic) or food (Topic) or Infant Nutritional Physiology (Topic) or Nutritional Physiology, Infant (Topic) or Physiology, Infant Nutritional (Topic) or Infant Nutritional Physiological Phenomenon (Topic) or Infant Nutrition Physiology (Topic) or Physiology, Infant Nutrition (Topic) or Nutrition Physiology, Infant (Topic) or Supplementary Feeding (Topic) or Feeding, Supplementary (Topic) or Feedings, Supplementary (Topic) or Supplementary Feedings (Topic) or Complementary Feeding (Topic) or Complementary Feedings (Topic) or Feeding, Complementary (Topic) or Feedings, Complementary (Topic)

#2 Atopic Dermatitides (Topic) or Atopic Dermatitis (Topic) or Dermatitides, Atopic (Topic) or Neurodermatitis, Atopic (Topic) or Atopic Neurodermatitides (Topic) or Atopic Neurodermatitis (Topic) or Neurodermatitides, Atopic (Topic) or Neurodermatitis, Disseminated (Topic) or Disseminated Neurodermatitides (Topic) or Disseminated Neurodermatitis (Topic) or Neurodermatitides, Disseminated (Topic) or Eczema, Atopic (Topic) or Atopic Eczema (Topic) or Eczema, Infantile (Topic) or Infantile Eczema (Topic) or Eczema

#3 Randomized controlled trial (Topic) or Non–randomized controlled trial (Topic) or Prospective cohort study (Topic) or Retrospective cohort study (Topic) or Case-control study (Topic) or Pre study control (Topic) or post study control (Topic)

#4 #1 AND#2 AND #3

**1.4 PubMed**

**Complementary feeding & Hypersensitivity**

(((("complementary feeding"[Title/Abstract] OR "infant nutritional physiological phenomena"[MeSH Terms] OR "infant nutritional physiological phenomena"[MeSH Terms] OR (("Infant"[MeSH Terms] OR "Infant"[All Fields] OR "infants"[All Fields] OR "infant s"[All Fields]) AND "nutritional physiology"[Title/Abstract]) OR "infant nutritional physiological phenomena"[MeSH Terms] OR (("nutrition s"[All Fields] OR "nutritional status"[MeSH Terms] OR ("Nutritional"[All Fields] AND "status"[All Fields]) OR "nutritional status"[All Fields] OR "Nutrition"[All Fields] OR "nutritional sciences"[MeSH Terms] OR ("Nutritional"[All Fields] AND "sciences"[All Fields]) OR "nutritional sciences"[All Fields] OR "Nutritional"[All Fields] OR "nutritionals"[All Fields] OR "nutritions"[All Fields] OR "nutritive"[All Fields]) AND "physiology infant"[Title/Abstract]) OR "infant nutritional physiological phenomena"[MeSH Terms] OR (("physiologies"[All Fields] OR "Physiology"[MeSH Subheading] OR "Physiology"[All Fields] OR "Physiology"[MeSH Terms]) AND "infant nutritional"[Title/Abstract]) OR "infant nutritional physiological phenomena"[MeSH Terms] OR ((("Infant"[MeSH Terms] OR "Infant"[All Fields] OR "infants"[All Fields] OR "infant s"[All Fields]) AND ("nutrition s"[All Fields] OR "nutritional status"[MeSH Terms] OR ("Nutritional"[All Fields] AND "status"[All Fields]) OR "nutritional status"[All Fields] OR "Nutrition"[All Fields] OR "nutritional sciences"[MeSH Terms] OR ("Nutritional"[All Fields] AND "sciences"[All Fields]) OR "nutritional sciences"[All Fields] OR "Nutritional"[All Fields] OR "nutritionals"[All Fields] OR "nutritions"[All Fields] OR "nutritive"[All Fields])) AND "physiological phenomenon"[Title/Abstract]) OR "infant nutritional physiological phenomena"[MeSH Terms] OR "infant nutrition physiology"[Title/Abstract] OR "infant nutritional physiological phenomena"[MeSH Terms] OR (("physiologies"[All Fields] OR "Physiology"[MeSH Subheading] OR "Physiology"[All Fields] OR "Physiology"[MeSH Terms]) AND "infant nutrition"[Title/Abstract]) OR "infant nutritional physiological phenomena"[MeSH Terms] OR (("nutrition s"[All Fields] OR "nutritional status"[MeSH Terms] OR ("Nutritional"[All Fields] AND "status"[All Fields]) OR "nutritional status"[All Fields] OR "Nutrition"[All Fields] OR "nutritional sciences"[MeSH Terms] OR ("Nutritional"[All Fields] AND "sciences"[All Fields]) OR "nutritional sciences"[All Fields] OR "Nutritional"[All Fields] OR "nutritionals"[All Fields] OR "nutritions"[All Fields] OR "nutritive"[All Fields]) AND "physiology infant"[Title/Abstract]) OR "infant nutritional physiological phenomena"[MeSH Terms] OR "supplementary feeding"[Title/Abstract] OR "infant nutritional physiological phenomena"[MeSH Terms] OR "feeding supplementary"[Title/Abstract] OR "infant nutritional physiological phenomena"[MeSH Terms] OR "feedings supplementary"[Title/Abstract] OR "infant nutritional physiological phenomena"[MeSH Terms] OR "supplementary feedings"[Title/Abstract] OR "infant nutritional physiological phenomena"[MeSH Terms] OR "complementary feeding"[Title/Abstract] OR "infant nutritional physiological phenomena"[MeSH Terms] OR "complementary feedings"[Title/Abstract] OR "infant nutritional physiological phenomena"[MeSH Terms] OR "feeding complementary"[Title/Abstract] OR "infant nutritional physiological phenomena"[MeSH Terms] OR (("Feeding"[All Fields] OR "Feedings"[All Fields] OR "feeds"[All Fields]) AND "Complementary"[Title/Abstract]) OR ("diet"[MeSH Terms] OR "diet"[All Fields] OR "diet"[Title/Abstract] OR "Food"[MeSH Terms] OR "foods"[Title/Abstract] OR "Food"[MeSH Terms] OR "Food"[Title/Abstract])) AND ("Hypersensitivity"[MeSH Terms] OR "Hypersensitivity"[Title/Abstract] OR "drug hypersensitivity"[MeSH Terms] OR "drug hypersensitivity"[Title/Abstract] OR "nut hypersensitivity"[MeSH Terms] OR "nut hypersensitivity"[Title/Abstract] OR "peanut hypersensitivity"[MeSH Terms] OR "peanut hypersensitivity"[Title/Abstract] OR "wheat hypersensitivity"[MeSH Terms] OR "wheat hypersensitivity"[Title/Abstract] OR "egg hypersensitivity"[MeSH Terms] OR "egg hypersensitivity"[Title/Abstract] OR "latex hypersensitivity"[MeSH Terms] OR "latex hypersensitivity"[Title/Abstract] OR "milk hypersensitivity"[MeSH Terms] OR "milk hypersensitivity"[Title/Abstract] OR "respiratory hypersensitivity"[MeSH Terms] OR "respiratory hypersensitivity"[Title/Abstract] OR "food hypersensitivity"[MeSH Terms] OR "food hypersensitivity"[Title/Abstract] OR "shellfish hypersensitivity"[MeSH Terms] OR "shellfish hypersensitivity"[Title/Abstract] OR (("common cold"[MeSH Terms] OR ("common"[All Fields] AND "Cold"[All Fields]) OR "common cold"[All Fields] OR "Cold"[All Fields] OR "cold temperature"[MeSH Terms] OR ("Cold"[All Fields] AND "temperature"[All Fields]) OR "cold temperature"[All Fields]) AND "Hypersensitivity"[MeSH Terms]) OR "cold hypersensitivity"[Title/Abstract]) AND ("randomized controlled trials as topic"[MeSH Terms] OR "randomized controlled trial"[Title/Abstract] OR (("Non-randomized"[All Fields] AND "controlled"[All Fields]) AND "clinical trials as topic"[MeSH Terms]) OR "non randomized controlled trial"[Title/Abstract] OR (("longitudinal studies"[MeSH Terms] OR ("longitudinal"[All Fields] AND "studies"[All Fields]) OR "longitudinal studies"[All Fields] OR "Prospective"[All Fields] OR "prospectively"[All Fields]) AND "cohort studies"[MeSH Terms]) OR "prospective cohort study"[Title/Abstract] OR (("retrospective studies"[MeSH Terms] OR ("retrospective"[All Fields] AND "studies"[All Fields]) OR "retrospective studies"[All Fields] OR "retrospective"[All Fields] OR "retrospectively"[All Fields] OR "retrospectives"[All Fields]) AND "cohort studies"[MeSH Terms]) OR ((("retrospective studies"[MeSH Terms] OR ("retrospective"[All Fields] AND "studies"[All Fields]) OR "retrospective studies"[All Fields] OR "retrospective"[All Fields] OR "retrospectively"[All Fields] OR "retrospectives"[All Fields]) AND ("cohort studies"[MeSH Terms] OR ("cohort"[All Fields] AND "studies"[All Fields]) OR "cohort studies"[All Fields] OR "cohort"[All Fields] OR "cohort s"[All Fields] OR "cohorte"[All Fields] OR "cohorts"[All Fields])) AND "studie"[Title/Abstract]) OR "case control study"[Title/Abstract] OR "case control study"[Title/Abstract] OR ((("phys rev e"[Journal] OR "phys rev e stat nonlin soft matter phys"[Journal] OR "pre"[All Fields]) AND ("studies"[All Fields] OR "study"[All Fields] OR "study s"[All Fields] OR "studying"[All Fields] OR "studys"[All Fields])) AND "control groups"[MeSH Terms]) OR (("phys rev e"[Journal] OR "phys rev e stat nonlin soft matter phys"[Journal] OR "pre"[All Fields]) AND "study control"[Title/Abstract]) OR (("post"[All Fields] AND ("studies"[All Fields] OR "study"[All Fields] OR "study s"[All Fields] OR "studying"[All Fields] OR "studys"[All Fields])) AND "control groups"[MeSH Terms]) OR ("post"[All Fields] AND "study control"[Title/Abstract]))) NOT ("reviews"[Title] OR "systematic reviews"[Title] OR "Meta-analyses"[Title])) NOT "cross sectional studies"[Title/Abstract]) NOT ("rat"[Title] OR "mice"[Title] OR "animal"[Title] OR "animals"[Title] OR "rabbit"[Title] OR "cat"[Title] OR "dog"[Title])

**Complementary feeding & Asthma**

("Infant Nutritional Physiological Phenomena"[MeSH Terms] OR ((("Infant"[MeSH Terms] OR "Infant"[All Fields] OR "infants"[All Fields] OR "infant s"[All Fields]) AND "nutritional physiology"[Title/Abstract]) OR ((("Infant"[MeSH Terms] OR "Infant"[All Fields] OR "infants"[All Fields] OR "infant s"[All Fields]) AND ("nutrition s"[All Fields] OR "nutritional status"[MeSH Terms] OR ("Nutritional"[All Fields] AND "status"[All Fields]) OR "nutritional status"[All Fields] OR "Nutrition"[All Fields] OR "nutritional sciences"[MeSH Terms] OR ("Nutritional"[All Fields] AND "sciences"[All Fields]) OR "nutritional sciences"[All Fields] OR "Nutritional"[All Fields] OR "nutritionals"[All Fields] OR "nutritions"[All Fields] OR "nutritive"[All Fields])) AND "physiological phenomenon"[Title/Abstract]) OR "infant nutrition physiology"[Title/Abstract] OR "supplementary feeding"[Title/Abstract] OR "feeding supplementary"[Title/Abstract] OR "feedings supplementary"[Title/Abstract] OR "supplementary feedings"[Title/Abstract] OR "complementary feeding"[Title/Abstract] OR "complementary feedings"[Title/Abstract] OR "feeding complementary"[Title/Abstract] OR "Diet"[Title/Abstract] OR "Food"[Title/Abstract]) OR "Food"[MeSH Terms] OR "Diet"[MeSH Terms]) AND ("Asthma"[MeSH Terms] OR ("Asthma"[Title/Abstract] OR "Bronchial"[Title/Abstract] OR "bronchial asthma"[Title/Abstract])) AND ("randomized controlled trial"[Title/Abstract] OR "non randomized controlled trial"[Title/Abstract] OR "prospective cohort study"[Title/Abstract] OR "retrospective cohort study"[Title/Abstract] OR "case control study"[Title/Abstract] OR (("phys rev e"[Journal] OR "phys rev e stat nonlin soft matter phys"[Journal] OR "pre"[All Fields]) AND "study control"[Title/Abstract]) OR ("post"[All Fields] AND "study control"[Title/Abstract]) OR "randomized controlled trials as topic"[MeSH Terms])

**Complementary feeding & Atopic dermatitis**

((("complementary feeding"[Title/Abstract] OR (("Infant"[MeSH Terms] OR "Infant"[All Fields] OR "infants"[All Fields] OR "infant s"[All Fields]) AND "nutritional physiology"[Title/Abstract]) OR (("nutrition s"[All Fields] OR "nutritional status"[MeSH Terms] OR ("Nutritional"[All Fields] AND "status"[All Fields]) OR "nutritional status"[All Fields] OR "Nutrition"[All Fields] OR "nutritional sciences"[MeSH Terms] OR ("Nutritional"[All Fields] AND "sciences"[All Fields]) OR "nutritional sciences"[All Fields] OR "Nutritional"[All Fields] OR "nutritionals"[All Fields] OR "nutritions"[All Fields] OR "nutritive"[All Fields]) AND "physiology infant"[Title/Abstract]) OR (("physiologies"[All Fields] OR "Physiology"[MeSH Subheading] OR "Physiology"[All Fields] OR "Physiology"[MeSH Terms]) AND "infant nutritional"[Title/Abstract]) OR ((("Infant"[MeSH Terms] OR "Infant"[All Fields] OR "infants"[All Fields] OR "infant s"[All Fields]) AND ("nutrition s"[All Fields] OR "nutritional status"[MeSH Terms] OR ("Nutritional"[All Fields] AND "status"[All Fields]) OR "nutritional status"[All Fields] OR "Nutrition"[All Fields] OR "nutritional sciences"[MeSH Terms] OR ("Nutritional"[All Fields] AND "sciences"[All Fields]) OR "nutritional sciences"[All Fields] OR "Nutritional"[All Fields] OR "nutritionals"[All Fields] OR "nutritions"[All Fields] OR "nutritive"[All Fields])) AND "physiological phenomenon"[Title/Abstract]) OR "infant nutrition physiology"[Title/Abstract] OR (("physiologies"[All Fields] OR "Physiology"[MeSH Subheading] OR "Physiology"[All Fields] OR "Physiology"[MeSH Terms]) AND "infant nutrition"[Title/Abstract]) OR (("nutrition s"[All Fields] OR "nutritional status"[MeSH Terms] OR ("Nutritional"[All Fields] AND "status"[All Fields]) OR "nutritional status"[All Fields] OR "Nutrition"[All Fields] OR "nutritional sciences"[MeSH Terms] OR ("Nutritional"[All Fields] AND "sciences"[All Fields]) OR "nutritional sciences"[All Fields] OR "Nutritional"[All Fields] OR "nutritionals"[All Fields] OR "nutritions"[All Fields] OR "nutritive"[All Fields]) AND "physiology infant"[Title/Abstract]) OR "supplementary feeding"[Title/Abstract] OR "feeding supplementary"[Title/Abstract] OR "feedings supplementary"[Title/Abstract] OR "supplementary feedings"[Title/Abstract] OR "complementary feeding"[Title/Abstract] OR "complementary feedings"[Title/Abstract] OR "feeding complementary"[Title/Abstract] OR (("Feeding"[All Fields] OR "Feedings"[All Fields] OR "feeds"[All Fields]) AND "Complementary"[Title/Abstract]) OR "Diet"[Title/Abstract] OR "Food"[Title/Abstract] OR ("infant nutritional physiological phenomena"[MeSH Terms] OR "Diet"[MeSH Terms] OR "Food"[MeSH Terms])) AND ("dermatitis atopic"[Title/Abstract] OR (("Atopic"[All Fields] OR "atopical"[All Fields] OR "atopics"[All Fields]) AND "Dermatitides"[Title/Abstract]) OR "atopic dermatitis"[Title/Abstract] OR (("dermatiti"[All Fields] OR "Dermatitis"[MeSH Terms] OR "Dermatitis"[All Fields] OR "Dermatitides"[All Fields]) AND "Atopic"[Title/Abstract]) OR "neurodermatitis atopic"[Title/Abstract] OR "atopic neurodermatitis"[Title/Abstract] OR "neurodermatitis disseminated"[Title/Abstract] OR "disseminated neurodermatitis"[Title/Abstract] OR "eczema atopic"[Title/Abstract] OR "atopic eczema"[Title/Abstract] OR "eczema infantile"[Title/Abstract] OR "infantile eczema"[Title/Abstract] OR "Eczema"[Title/Abstract] OR "randomized controlled trial"[Title/Abstract]) AND ("randomized controlled trial"[Title/Abstract] OR "non randomized controlled trial"[Title/Abstract] OR "prospective cohort study"[Title/Abstract] OR "retrospective cohort study"[Title/Abstract] OR "case control study"[Title/Abstract] OR (("phys rev e"[Journal] OR "phys rev e stat nonlin soft matter phys"[Journal] OR "pre"[All Fields]) AND "study control"[Title/Abstract]) OR ("post"[All Fields] AND "study control"[Title/Abstract]) OR "randomized controlled trials as topic"[MeSH Terms])) NOT ("dog"[Title] OR "rats"[Title] OR "mice"[Title] OR "mouse"[Title] OR "rabbit"[Title] OR "animal"[Title])) NOT ("systematic review"[Title] OR "Meta-Analysis"[Title])
